# A distinct CAR-T cell phenotype mediates therapeutic response at limited doses

**DOI:** 10.1038/s41467-026-76068-4

**Published:** 2026-07-29

**Authors:** Schayan Yousefian, Maria-Luisa Schubert, Anna Rita Minafra, Patrick Derigs, Sarah Gräßle, Arik Horne, Uta M. Demel, Julian Liebaert, Caroline Röthemeier, Franziska Pupp, Uta E. Höpken, Jan Krönke, Antonia Busse, Ulrich Keller, Anita Schmitt, Daniel Hübschmann, Carsten Müller-Tidow, Peter Dreger, Michael Schmitt, Simon Haas

**Affiliations:** 1 Berlin Institute of Health (BIH) at Charité Universitätsmedizin Berlin, Berlin, Germany; 2 Berlin Institute for Medical Systems Biology (BIMSB), Max-Delbrück-Center for Molecular Medicine in the Helmholtz Association (MDC), Berlin, Germany; 3 Charité – Universitätsmedizin Berlin, corporate member of Freie Universität Berlin and Humboldt-Universität zu Berlin, Department of Hematology, Oncology and Tumor Immunology, Berlin, Germany; 4 Internal Medicine V, Hematology, Oncology and Rheumatology, Heidelberg University Hospital, Heidelberg, Germany; 5 Humboldt-Universität zu Berlin, Institute of Biology, Berlin, Germany; 6 Computational Oncology Group, Molecular Precision Oncology Program, National Center for Tumor Diseases (NCT) Heidelberg and German Cancer Research Center, Heidelberg, Germany; 7 Innovation and Service Unit for Bioinformatics and Precision Medicine (BPM), German Cancer Research Center, Heidelberg, Germany; 8 German Cancer Consortium (DKTK), Partner Site Berlin, Berlin, Germany; 9 Max-Delbrück-Center for Molecular Medicine (MDC), Berlin, Germany; 10 Clinician Scientist Program, Berlin Institute of Health (BIH), Berlin, Germany; 11 Department of Microenvironmental Regulation in Autoimmunity and Cancer, Max Delbrück Center for Molecular Medicine, Berlin, Germany; 12 Internal Medicine C, Hematology, Oncology, Stem Cell Transplantation and Palliative Care, University Medicine Greifswald, Greifswald, Germany; 13 Cluster of Excellence ImmunoPreCept, Charité—Universitätsmedizin Berlin, Berlin, Germany; 14 German Cancer Consortium (DKTK), Partner Site Heidelberg, Heidelberg, Germany; 15 Division of Translational Precision Medicine, Institute of Human Genetics, Heidelberg University, Heidelberg, Germany; 16 National Center for Tumor Diseases (NCT), Heidelberg, Germany; 17 Precision Healthcare University Research Institute, Queen Mary University of London, London, UK

**Keywords:** Immunotherapy, Predictive markers, Haematological cancer

## Abstract

Chimeric antigen receptor (CAR) T-cell therapies are typically administered at high doses to maximize durable clinical responses. However, manufacturing constraints can limit the production of sufficient cells to achieve the intended dose. Although some patients experience durable responses after receiving lower CAR-T cell doses, the mechanisms underlying efficacy at these limited cell numbers remain poorly understood. To address this, we performed deep phenotyping of anti-CD19 CAR-T cell products and their corresponding leukapheresis starting materials from a phase I/II dose-escalation trial. We also report the primary and secondary clinical outcomes of the diffuse large B-cell lymphoma, follicular lymphoma, and mantle cell lymphoma cohorts of the HD-CAR-1 basket trial (NCT03676504; EudraCT 2016-004808-60). Patients responding to low-dose CAR-T therapy showed dose-dependent enrichment of functional effector and effector memory-like CAR-T cells. The absolute number of these cells emerged as a robust biomarker of therapeutic response, valid across dose levels and CAR-T targets. Effective low-dose products were associated with high T-cell numbers and T-cell-supportive myeloid states in leukapheresis materials, whereas regulatory myeloid states promoted dysfunctional CAR-T cells and treatment failure. Our study provides insights into the phenotype, mechanisms, and biomarkers of CAR-T cells that drive therapeutic responses at low doses, with medical and socioeconomic implications.

## Abstract (web summary)

Predictors of response to low-yield or low-dose CAR-T cell therapy remain poorly characterized. The authors here show that the therapeutic efficacy and response are associated with absolute numbers of CD27^-^CD39^-^ effector-like CAR-T cells and show that distinct myeloid programs in the pre-manufacturing leukapheresis material influence their generation.

## Introduction

Chimeric antigen receptor (CAR) T cells have transformed the therapy landscape of hematological malignancies^1–3^, including B cell non-Hodgkin’s lymphoma (NHL)^4–9^, B cell acute lymphoblastic leukemia (ALL)^10–12^ and multiple myeloma^13–15^ and are increasingly being explored for autoimmune diseases^16–23^ and other cancer types^24,25^. Notably, anti-CD19 CAR-T cells have become a standard therapy for relapsed or refractory B-cell malignancies, yielding high response rates and significantly improving patient outcomes. To ensure sustained therapeutic responses and meet regulatory standards, CAR-T cells are typically manufactured and administered in large quantities. While higher doses are generally associated with a better outcome^26^, patients receiving lower doses can also achieve therapeutic responses^27,28^. Although the molecular, cellular and microenvironmental factors associated with response have been extensively studied in patients receiving CAR-T cells in excess^29–37^, these factors remain poorly understood in cases where only low numbers of CAR-T cells are available^38^.

The CAR-T cell manufacturing process must comply with strict regulatory requirements, such as manufacturing sufficient CAR-T cells as defined in the end-product specifications. Deviations will result in so called out-of-specification (OOS) products. Failure rates in CAR-T cell manufacturing, specifically due to the inability to meet the targeted dose, can vary from 1% to 13%^4,39–42^. Depending on the regulatory environment, these products may not be eligible for patient administration or may only be used under specific conditions at the discretion of the treating physician. Safety, efficacy, release and application of OOS anti-CD19 or anti-BCMA infusion products have been explored in several different clinical trials (NCT03601442: tisagenlecleucel, Novartis; NCT05347485: ciltacabtagene autoleucel, Janssen; NCT05776160; axicabtagene ailoleucel, Kite/Gilead; NCT04094311: tisagenlecleucel, Novartis). Notably, commercial CAR-T cell manufacturers are not reimbursed for OOS products, which significantly contributes to the overall high costs of CAR-T cell therapies, comprising expenses associated with failed attempts. This creates a significant financial burden on healthcare systems and limits patient access to life-saving treatments. Biomarkers predicting the efficacy of CAR-T cells that fall short of the required cell numbers have the potential to expand access, reduce costs, and lead to revisions in regulatory standards.

Despite the urgent medical need, most studies on CAR-T cell efficacy have concentrated on patients receiving high doses. Given the exceptional response rates observed with high-dose CAR-T cells, most research has focused on CAR-T cell persistence, expansion and the duration of response. In this context, early lineage CAR-T cell states, particularly those with naïve and (stem cell) memory phenotypes, were linked to enhanced CAR-T cell expansion and sustained therapeutic response^10,36,40,43–50^. However, it remains to be elucidated whether similar or distinct CAR-T cell populations drive therapeutic responses in low-dose patients when compared to those receiving higher doses. In patients receiving limited CAR-T cell doses, achieving an initial response mediated by effector-like therapeutic cells may hold greater importance than ensuring long-term durability mediated by more naïve- or central memory-like CAR-T cells^50,51^. Additionally, microenvironmental factors that may influence the production of effective low-dose CAR-T cells have yet to be determined.

Here, we performed deep profiling of anti-CD19 CAR-T cell products and corresponding leukapheresis starting materials from the entire HD-CAR-1 trial cohort to identify cellular determinants of therapeutic response at limited doses. We show that low-dose response is associated with functional effector and effector memory-like CAR-T cells, identify the absolute number of CD27⁻CD39⁻ CAR-T cells as a biomarker of response across dose levels, and link effective products to T-cell-rich and T-cell-supportive myeloid states in leukapheresis materials. In this context, we additionally report the primary and secondary clinical outcomes of the DLBCL, FL, and MCL arms of the phase I/II HD-CAR-1 basket trial (NCT03676504; EudraCT 2016-004808-60). Adults with relapsed or refractory CD19-positive disease, adequate performance status and organ function, and no uncontrolled infection or major concurrent illness were eligible. The primary endpoints were safety and manufacturing feasibility, whereas secondary endpoints included day-90 response, duration of response, progression-free survival, and overall survival. Long-term responses were observed in 40% of patients with lymphoma. Treatment showed an excellent safety profile, with only grade 1 or 2 cytokine release syndrome and no immune effector cell-associated neurotoxicity syndrome, other neurological toxicities, or dose-limiting toxicities.

## Results

### CAR-T cells of low-dose responders display a distinct phenotype

To characterize CAR-T cells capable of driving clinical responses despite limited input numbers, we first conducted a comprehensive analysis of the CD19-directed CAR-T cell products. These products were administered in the framework of the “Heidelberg CAR-T cell trial 1” (HD-CAR-1, NCT03676504) a phase I/II clinical dose-escalation study evaluating the safety and efficacy of third-generation CAR-T cells in patients with relapsed and/or refractory B-cell malignancies^52–54^. A total of 28 patients were included, receiving CAR-T cell products, administered at doses ranging from 1 × 10⁶ to 200 × 10⁶ CAR-T cells per square meter (m^2^) of body surface area. The inclusion criteria, patient characteristics, and clinical parameters of the HD-CAR-1 trial have been previously reported^53,54^ and are summarized in Supplementary Data 1.

**Fig. 1:**
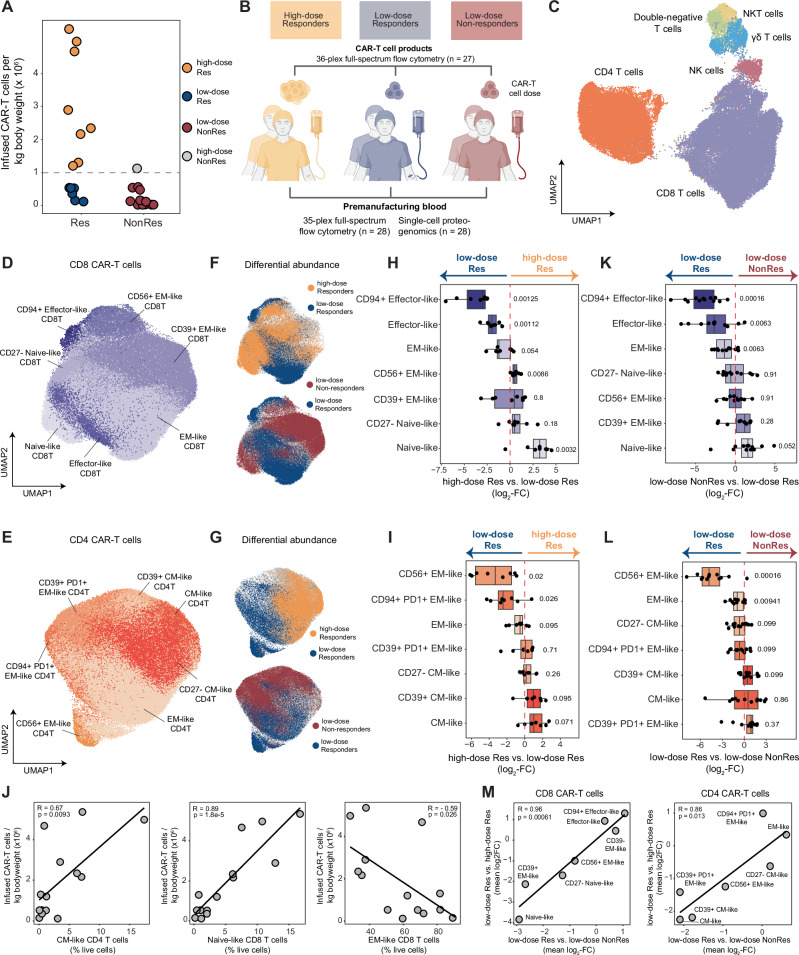
Low-dose responder CAR-T cells exhibit a distinct phenotype. **A** Number of CAR-T cells per kg body weight per patient and their response to CAR-T cell therapy. **B** Overview of patient groups and experimental setup. Created in BioRender. Röthemeier, C. (2026) https://BioRender.com/nssj5kq. **C** UMAP of CAR-T cell infusion products (*n* = 27) obtained by full-spectrum flow cytometry. Out of 10,129,369 cells, 66,121 sketched cells are shown. **D** UMAP of CD8 CAR-T cells of the infusion product (*n* = 27). Out of 3,020,704 cells, 149,398 sketched cells are displayed. **E** UMAP of CD4 CAR-T cells of the infusion product (*n* = 27). Out of 1,836,940 cells, 146,426 sketched cells are displayed. **F** Differential abundance within CD8 CAR-T cells using DA-seq^85^ comparing high-dose to low-dose responders (top) and low-dose non-responders to low-dose responders (bottom). **G** Differential abundance within CD4 CAR-T cells using DA-seq comparing high-dose to low-dose responders (top) and low-dose non-responders to low-dose responders (bottom). **H** Frequencies within CD8 CAR-T cells comparing high-dose to low-dose responders. A one-sample t-test (two-sided) was applied. *P* values were adjusted using Benjamini-Hochberg (*n* = 8 patients). **I** Frequencies within CD4 CAR-T cells comparing high-dose to low-dose responders. A one-sample t-test (two-sided) was applied. *P* values were adjusted using Benjamini-Hochberg (*n* = 8 patients). **J** Pearson correlation of infused number of CAR-T cells within all responders and frequencies of naïve-like CD4 T cells (left panel), naïve-like CD8 T cells (middle panel) and EM-like CD8 T cells (right panel). The correlation coefficient and *p* value were calculated using a two-sided Pearson correlation test. *n* = 14 patients. **K** Comparison of frequencies within CD8 CAR-T cells comparing low-dose non-responders to responders. A one-sample t-test (two-sided) was applied. *P* values were adjusted using Benjamini-Hochberg (*n* = 12 patients). **L** Frequencies within CD4 CAR-T cells comparing low-dose non-responders to responders. A one-sample t-test (two-sided) was applied. *P* values were adjusted using Benjamini-Hochberg (*n* = 12 patients). **M** Pearson correlation of mean log_2_-FC per CD8/CD4 CAR-T cell type (UMAP in **D**/**E**, left/right panel) obtained by comparing high-dose to low-dose responders and comparing low-dose non-responders to low-dose responders. Number of cell types (*n* = 7). The correlation coefficient and *p* value were calculated using a two-sided Pearson correlation test. Abbreviations: UMAP uniform manifold approximation and projection, Res Responder, NonRes Non-responder, NK cells natural killer cells, NKT cells natural killer T cells, EM effector-memory, CM central-memory, act. activated, kg kilogram, FC fold-change. Box plots display the median, first and third quartiles and whiskers are defined as 1.5 times the interquartile range.

As expected, patients receiving CAR-T cell doses within commonly administered ranges from 50 × 10^6^ to 200 × 10^6^ per m^2^ (referred to as high-dose; corresponding to a range from 1.1 × 10^6^ to 5.35 × 10^6^ per kg body weight) demonstrated high response rates (Fig. 1A). Conversely, patients treated with lower CAR-T cell doses from 1 × 10^6^ to 20 × 10^6^ per m^2^ (referred to as low-dose; corresponding to a range from 0.02 × 10^6^ to 0.5 × 10^6^ per kg body weight) were less likely to respond. Based on the administered CAR-T cell doses and the clinical response outcomes, we categorized patients into three groups: (1) high-dose responders (*n *= 8), who achieved clinical response after receiving high CAR-T cell doses; (2) low-dose non-responders (*n* = 12), who failed to respond after receiving low-dose CAR-T cells; and (3) low-dose responders (*n* = 6), a notable subset of patients who achieved robust clinical responses despite receiving low CAR-T cell doses (Fig. 1A, B). Notably, therapy response was not significantly associated with sex, age, previous allogeneic stem cell transplantation or disease indication (Supplementary Table 1, Supplementary Fig. 1A). Within the low-dose group, clinical responses appeared durable with a trend towards longer overall survival (OS) and progression-free survival (PFS) in low-dose responders compared to low-dose non-responders (Supplementary Fig. 1B). Moreover, low-dose responders and low-dose non-responders displayed comparable numbers of CAR+ cells in their infusion products and showed similar CAR-T cell expansion in vivo, as reflected by similar peak levels within the first 28 days post-infusion (Supplementary Fig. 1C-E). In contrast, high-dose responders displayed significantly higher frequencies of CAR+ cells in the infusion product and, consequently, higher absolute CAR-T cell numbers in vivo (Supplementary Fig. 1C-E). Notably, across all groups, we observed significant correlations between the infused CAR-T cell dose, the in vivo expansion of CAR-T cells, and progression-free survival (Supplementary Fig. 1F, G).

To explore potential relationships between administered CAR-T cell dose levels, clinical responses, and the underlying cellular states of CAR-T cell products, we performed full-spectrum flow cytometry using a 36-plex panel custom-designed to evaluate T cell states. Following stringent quality control, clustering analysis, and UMAP visualization (Supplementary Fig. 2A, Methods), we identified diverse subsets of CD4 T cells, CD8 T cells, γδ T cells, and natural killer (NK) cells within the CAR-T cell products comprising a total of 10,129,369 high-quality cells (Fig. 1C; Supplementary Fig. 2B). Within the CD4 and CD8 CAR-T cells, we identified non-activated subsets of cells including naïve and central-memory (CM)-like CD4 and CD8 CAR-T cells as well as activated populations including effector-memory and effector-like CAR-T cells (Fig. 1D, E, Supplementary Fig. 2C–F). Among the CD4 and CD8 effector memory-like populations, a pronounced subpopulation was characterized by elevated expression of exhaustion markers such as CD39 and PD1, indicating their potential dysfunctional activation during manufacturing. In addition, a range of other effector memory-like populations with no phenotypic signs of exhaustion were identified.

To investigate whether CAR-T cells from low-dose responders exhibit a specific phenotype that enables response despite low numbers, we first compared them to their counterparts from responders that received high CAR-T cell doses (Fig. 1D–I). In line with previous reports^36,47,48,55^, CD4 and CD8 CAR-T cells from responders that received high CAR-T cell doses were enriched in naïve and central memory-like states. In contrast, CD4 and CD8 CAR-T cells from low-dose responders displayed a distinct phenotype, highly enriched in non-exhausted effector-memory and effector-like states (Fig. 1F–I). Notably, within patients responding to CAR-T cell therapy, the overall administered CAR-T cell dose was negatively correlated with the relative abundance of effector memory-like CD8 CAR-T cells, while the relative abundance of naïve-like and CM-like CAR-T cells showed a positive correlation with the administered dose (Fig. 1J). These findings suggest that CAR-T cells of patients achieving response to therapy at low dose levels are relatively enriched for CAR-T cells with an effector or effector memory-like phenotype in a dose-dependent manner.

To investigate whether the cellular states enriched in low-dose responders are indeed associated with response to CAR-T cell therapy, we compared their CAR-T cell products with those from low-dose non-responders (Fig. 1K, L). Notably, the CAR-T cell populations enriched in low-dose responders versus non-responders closely overlapped with those enriched in low-dose responders versus those responding with excessive doses (Fig. 1M). Consistent with our prior findings, CAR-T cell products from low-dose responders were significantly enriched in effector- or effector memory-like CD4 and CD8 T cell states, when compared to low-dose non-responders (Fig. 1F, K, G, L). In contrast, CAR-T cells from low-dose non-responders were predominantly enriched in naïve-like states or exhibited features of exhaustion and dysfunction, characterized by elevated co-expression of markers such as CD39 and PD1, indicating either insufficient or dysfunctional activation during production.

Collectively, these observations suggest that CAR-T cells from low-dose responders are characterized by a distinct phenotype, with the presence of functional effector-memory and effector-like CAR-T cells being a crucial factor in achieving therapeutic response in patients receiving limited CAR-T cell doses.

### Absolute number of functional effector-like CAR-T cells determines the response

**Fig. 2:**
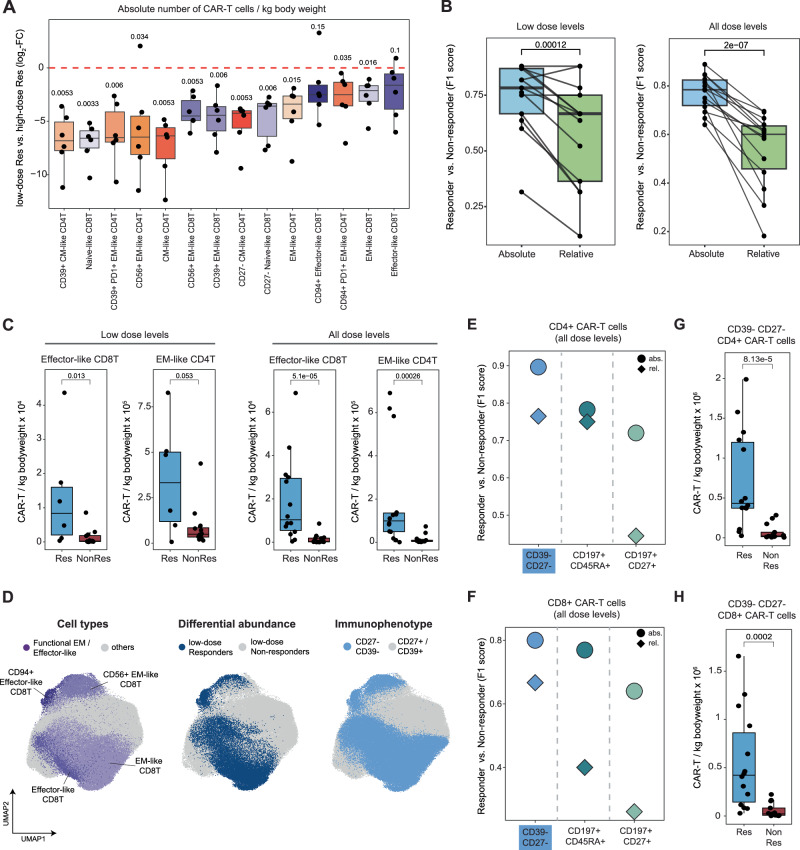
Therapy response is determined by the absolute number of functional effector-like CAR-T cells. **A** Quantification of absolute numbers of infused CD4 and CD8 CAR-T cell subsets per kilogram of body weight comparing low-dose to high-dose responders. A one-sample t-test (two-sided) was applied, and p values were adjusted using Benjamini-Hochberg correction (*n* = 6 patients). **B** F1 score per cell type from A to discriminate responders from non-responders using absolute numbers of infused CAR-T cell subsets or respective relative frequencies. Left panel: low-dose patients, *n* = 13 paired cell types. Right panel: all patients. *n* = 14 paired cell types. A paired t-test (two-sided) was applied. **C** Boxplots comparing the absolute number of infused CAR-T cell subsets per kg of body weight (top features from **B**) between responders and non-responders. Left panel: low-dose patients (*n* = 18 patients). Right panel: all patients (*n* = 27 patients). A two-sided Wilcoxon rank-sum test was applied. **D** UMAPs of the CD8 CAR-T cells of the infusion product (*n* = 27). Left panel: Functional effector or EM-like populations are highlighted in purple. Middle panel: Differential abundance comparing low-dose responders to low-dose non-responders. Right panel: Highlighted immunophenotype based on absence of CD27 and CD39 expression. **E** F1 score of the provided biomarker combination (CD27⁻CD39⁻) and published biomarkers within CD4 CAR-T cells to discriminate responders from non-responders within all dose levels. **F** F1 score of the provided biomarker combination (CD27⁻CD39⁻) and published biomarkers within CD8 CAR-T cells to discriminate responders from non-responders within all dose levels. **G** Boxplots comparing absolute numbers of CD4⁺CD27⁻CD39⁻ infused CAR-T cells per kg of body weight. A two-sided Wilcoxon rank-sum test was applied. *P* values were corrected using the Benjamini-Hochberg method. Res: *n* = 14, NonRes: *n* = 13. **H** Boxplots comparing the absolute number of CD8⁺CD27⁻CD39⁻ infused CAR-T cells per kg of body weight. A two-sided Wilcoxon rank-sum test was applied. *P* values were corrected using the Benjamini-Hochberg method. Res: *n* = 14, NonRes: *n* = 13. Abbreviations: UMAP uniform manifold approximation and projection, Res Responder, NonRes Non-responder, EM effector-memory, CM central-memory, kg kilogram, FC fold-change. Box plots display the median, first and third quartiles and whiskers are defined as 1.5 times interquartile range.

Previous biomarker studies for predicting response to CAR-T cell therapy have primarily focused on patients receiving high numbers of CAR-T cells, typically analyzing relative frequencies of T cell states or marker expression within the infusion product^47,48^. However, in patients receiving low numbers of CAR-T cells, the absolute number of cellular subsets with immediate therapeutic efficacy might be more critical for mediating the initial treatment response. To test this hypothesis, we first compared the absolute numbers of infused CAR-T cell populations per kilogram of body weight of low-dose and high-dose responders. As expected, patients receiving high CAR-T cell doses had significantly higher numbers of most CAR-T cell subpopulations compared to those receiving low doses (Fig. 2A). However, the absolute numbers of effector-like CAR-T cell subsets were comparable between low-dose responders and high-dose responders, despite the markedly lower overall number of administered CAR-T cells in the former group (Fig. 2A). These findings suggest that achieving a therapeutic response may require reaching a threshold number of therapeutically active CAR-T cell populations. Supporting this, the quantification of absolute numbers of CD4 and CD8 CAR-T cell subsets generally outperformed relative frequency analyses in predicting responses, both at low dose levels and across all dose levels (Fig. 2B). Consistent with our previous findings, the absolute numbers of effector-like and effector memory-like CAR-T cells lacking markers of phenotypic exhaustion or dysfunction, i.e., functional cells, proved most effective at discriminating responders from non-responders, both at low dose levels and across all dose levels (Fig. 2C; Supplementary Fig. 3A, B).

Notably, the combined absence of CD27 and CD39 expression in CD4 and CD8 CAR-T cell subsets, proved to be a robust biomarker combination for identifying effector-like states in the absence of phenotypic exhaustion (Fig. 2D, Supplementary Fig. 3 C). This biomarker combination outperformed previously suggested predictor combinations^47,48^, both when quantified in relative or absolute numbers in CD4 and CD8 CAR-T cell subsets (Fig. 2E-H). Biomarker levels of the CD27⁻CD39⁻ subset in CD4 and CD8 T cell populations were tightly correlated and emerged as strong predictors of treatment response, whereas their associations with other covariates were negligible (Supplementary Fig. 4A). Lactate dehydrogenase (LDH) levels, a proxy for tumor burden, displayed a more modest association with response and were largely independent of the biomarker combination (Supplementary Fig. 4A, B). Notably, although LDH levels were associated with overall survival (OS), they were not associated with progression-free survival (PFS) (Supplementary Fig. 1H, E). In contrast, the CD27⁻CD39⁻ biomarker combination was associated with PFS but not OS, further supporting the independence of these two factors. To explore their combined prognostic value, patients were stratified into high versus low frequencies of biomarker-associated CAR-T cells (CD27⁻CD39⁻), and a composite variable integrating LDH status (high/low) with biomarker status (high/low) was generated. Kaplan-Meier analyses based on this composite stratification revealed that patients with low LDH and high biomarker levels had the most favorable OS, whereas those with high LDH and low biomarker levels had the poorest outcomes (Supplementary Fig. 4C). A similar pattern emerged for PFS. Patients with high biomarker levels exhibited the most favorable PFS irrespective of LDH status, consistent with the minimal effect of LDH on PFS when considered independently (Supplementary Fig. 4C).

Collectively, these results emphasize that absolute numbers of functional effector-like CAR-T cells, identified by the lack of CD27 and CD39 expression, are a decisive factor in mediating therapeutic responses in patients receiving CAR-T cells at varying dose levels.

### CD27⁻CD39⁻ CAR-T cells define an effector subset with limited plasticity and high cytotoxic potency

**Fig. 3:**
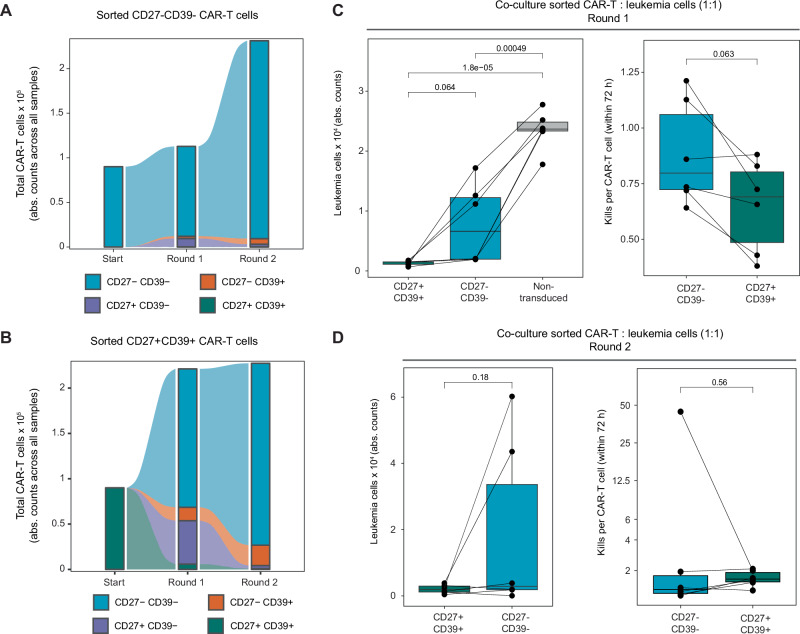
CD27⁻CD39⁻ CAR-T cells represent a terminal effector subset with limited plasticity and high cytotoxic potency. **A** Alluvial plot showing CAR-T cell expansion (absolute cell numbers summed across all six healthy donors) from sorted CD27⁻CD39⁻ CAR-T cells across two sequential co-culture rounds with Nalm6 leukemia cells. **B** Alluvial plot showing CAR-T cell expansion (absolute cell numbers summed across all six healthy donors) from sorted CD27⁺CD39⁺ CAR-T cells across two sequential co-culture rounds with Nalm6 leukemia cells. **C** Left: quantification of residual leukemia cells after the first co-culture round. P values were determined with a paired, two-sided t-test. *n* = 6 healthy donors. Right: per CAR-T cell killing efficiency in round 1, normalized for total CAR-T cell abundance and leukemia cell growth. P values were determined with a paired, two-sided Wilcoxon rank-sum test. *n* = 6 healthy donors. **D** Left: quantification of residual leukemia cells after the second co-culture round. *P* values were determined with a paired, two-sided t-test. *n* = 6 healthy donors. Right: per CAR-T cell killing efficiency in round 2, normalized for total CAR-T cell abundance and leukemia cell growth. *P* values were determined with a paired, two-sided Wilcoxon rank-sum test. *n* = 6 healthy donors. Box plots display the median, first and third quartiles and whiskers are defined as 1.5 times the interquartile range.

To characterize the CD27⁻CD39⁻ CAR-T cell subset functionally, we performed in vitro assays. In co-culture experiments, sorted CD27⁻CD39⁻ CAR-T cells from six healthy donors were incubated with Nalm6 leukemia cells at a 1:1 effector-to-target ratio for two sequential 72-h co-cultures. CD27⁺CD39⁺ CAR-T cells were included as control population. Across both incubation series, CD27⁻CD39⁻ CAR-T cells maintained a stable terminal effector phenotype with minimal phenotypic plasticity, whereas CD27⁺CD39⁺ CAR-T cells generated a broader spectrum of phenotypes, including CD27⁻CD39⁻ progeny (Fig. 3A, B). Notably, CD27⁺CD39⁺ CAR-T cells showed robust expansion during the first co-culture and more than doubled in number (Fig. 3B), while CD27⁻CD39⁻ cells exhibited limited proliferation, consistent with their effector state (Fig. 3A). Both subsets mediated potent leukemia clearance compared with non-transduced controls (Fig. 3C). However, when normalized for CAR-T expansion and leukemia growth, the CD27⁻CD39⁻ CAR-T cells displayed a more efficient killing in the first co-culture round, which converged in the second co-culture round, when both CAR-T cell populations shifted toward a predominantly CD27⁻CD39⁻ effector phenotype (Fig. 3C, D). Multiplex cytokine profiling of co-culture supernatants revealed enrichment of key effector cytokines (including IFN-γ, TNF, and IL-2) for both CAR-T subsets relative to non-transduced controls, without a consistent cytokine signature distinguishing CD27⁻CD39⁻ from CD27⁺CD39⁺ CAR-T cells (Supplementary Fig. 5A). Together, these findings support the interpretation that CD27⁻CD39⁻ CAR-T cells represent a highly functional, terminal effector subset characterized by a stable phenotype, limited plasticity, and high cytotoxic potency.

### Therapeutic efficacy of low-dose CAR-T cells is associated with the pre-manufacturing blood composition

**Fig. 4:**
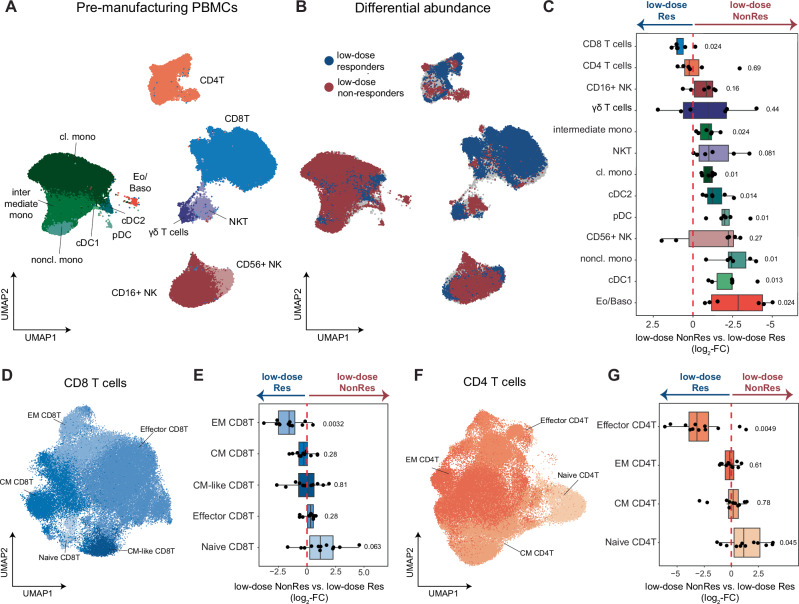
Composition of pre-manufacturing blood is associated with therapeutic efficacy of low-dose CAR-T cells. **A** UMAP of the cellular landscape of pre-manufacturing blood (*n* = 28) obtained by 35-plex full-spectrum flow cytometry. Out of 14,266,855 high-quality cells, 118,601 sketched cells are shown. **B** Differential abundance using DA-seq within pre-manufacturing blood comparing low-dose responders to non-responders. **C** Quantification of relative frequencies within pre-manufacturing blood comparing low-dose responders to non-responders. A one-sample t-test (two-sided) was applied, and p values were adjusted using Benjamini-Hochberg correction (*n* = 6 patients). **D** UMAP display of CD8 T cells of the pre-manufacturing blood (*n *= 28 patients). Out of 3,863,987 high-quality cells, 57,603 sketched cells are displayed. **E** Quantification of relative frequencies within CD8 T cells comparing low-dose non-responders to responders. A one-sample t-test (two-sided) was applied, and p values were adjusted using Benjamini-Hochberg correction (*n* = 12 patients). **F** UMAP display of CD4 T cells of the pre-manufacturing blood (*n* = 28 patients). Out of 1,267,718 high-quality cells, 63,031 sketched cells are displayed. **G** Quantification of relative frequencies within CD4 T cells comparing low-dose non-responders to responders. A one-sample t-test (two-sided) was applied, and *p* values were adjusted using Benjamini-Hochberg correction (*n* = 12 patients). Abbreviations: UMAP uniform manifold approximation and projection, Res Responder, NonRes Non-responder, cDC conventional dendritic cells, pDC plasmacytoid dendritic cells, Eo/baso eosinophil-basophils, cl.mono classical monocytes, noncl. mono non-classical monocytes, NK cells natural killer cells, EM effector-memory, CM central-memory, FC fold-change. Box plots display the median, first and third quartiles and whiskers are defined as 1.5 times the interquartile range.

To investigate the mechanisms underlying therapeutic response at low doses, we performed deep-phenotyping of pre-manufacturing peripheral blood mononuclear cells (PBMCs) of the patient cohort introduced above before apheresis, i.e., prior to CAR-T cell manufacturing (*n* = 28). Following quality control, this analysis captured a diverse cellular landscape comprising 14,266,855 high-quality cells. These cells encompassed various populations, including CD4 and CD8 T cell subsets, γδ T cells, NK-T cells, NK cell subpopulations, classical, intermediate, and non-classical monocytes, conventional and plasmacytoid dendritic cells (DCs), and eosinophil-basophils (Fig. 4A, Supplementary Fig. 6A). A comparison between low-dose responders and non-responders revealed that the pre-manufacturing blood of low-dose responders was significantly enriched in CD8 T cells (Fig. 4B, C, Supplementary Fig. 6D). Within the T cell compartment, low-dose responders displayed a higher abundance of effector and effector memory states (Fig. 4D-G; Supplementary Fig. 6B, C, E). Conversely, the pre-manufacturing blood of low-dose non-responders was highly enriched in various myeloid populations, including classical and non-classical monocytes, conventional DCs, plasmacytoid DCs, and eosinophils/basophils (Fig. 4B, C). Within the myeloid compartment, particularly non-classical monocytes and dendritic cell subsets were associated with non-response at low dose levels (Supplementary Fig. 6F, G). Comparisons of pre-manufacturing blood between disease subtypes revealed no major differences in cellular composition and T cell phenotype (Supplementary Fig. 7A-C).

Collectively, these findings suggest that low-dose responders exhibit a distinct pre-manufacturing blood composition that is not primarily driven by disease subtype, marked by a lower abundant myeloid compartment and elevated effector memory T cell states - mirroring the traits of their post-manufacturing CAR-T cell product.

### Molecular programs of pre-manufacturing myeloid populations underly therapy response

**Fig. 5:**
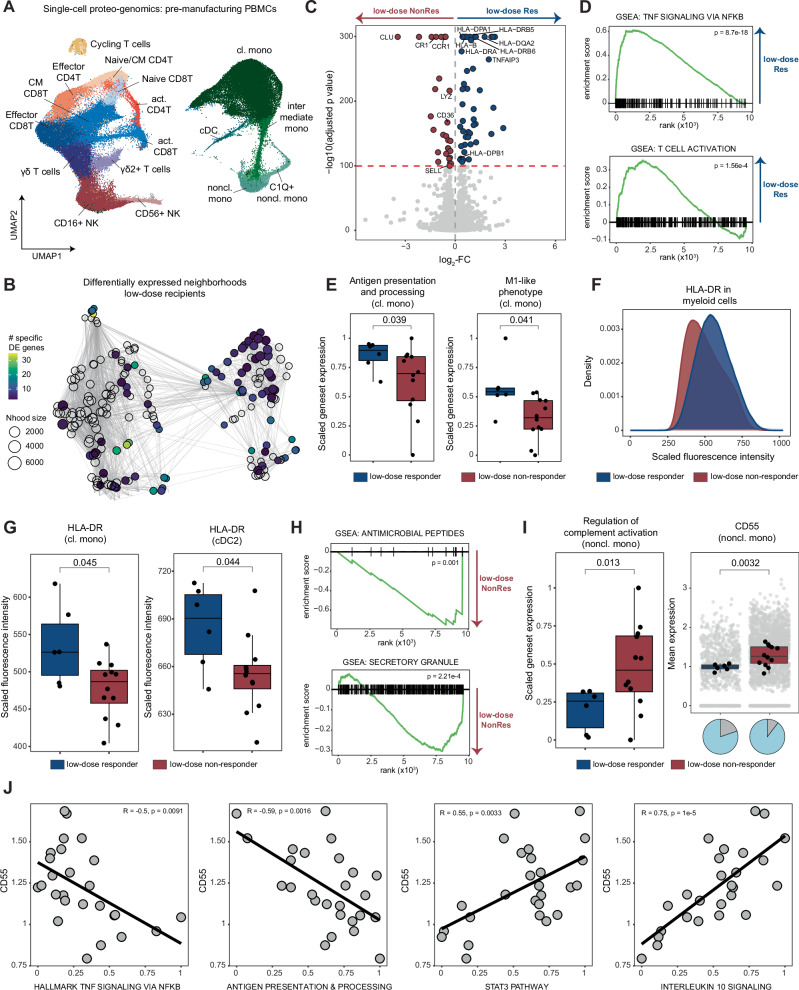
Therapy response is associated with molecular programs of pre-manufacturing myeloid subsets. **A** UMAP of the cellular landscape of pre-manufacturing blood (*n* = 28) obtained by single-cell proteo-genomics. 112,442 high-quality cells are displayed. **B** Cellular neighborhoods with differentially expressed genes within low-dose recipients obtained by miloDE^87^. **C** Volcano plot highlighting differentially expressed genes between low-dose responders and non-responders in all myeloid populations. *P* values were calculated using the Wald test and adjusted for multiple testing using the Benjamini-Hochberg method. **D** Gene set enrichment analysis of gene sets enriched in low-dose responders (*n* = 6 patients). Pathway *p* values were estimated using an adaptive multilevel splitting Monte Carlo approach based on enrichment scores calculated from the ranked gene-level statistic. Benjamini-Hochberg corrected *p* values are displayed. **E** Boxplots of differentially expressed gene sets in classical monocytes of low-dose recipients. Min-max scaled expression values are displayed. A two-sample t-test (two-sided) was applied. *n* = 18 patients. **F** Density histogram of HLA-DR fluorescence intensity in myeloid cells of low-dose recipients (*n *= 18 patients). **G** Boxplots of HLA-DR scaled fluorescence intensity in classical monocytes and cDC2 cells of low-dose recipients (*n* = 18 patients). A two-sample t-test (two-sided) was applied. **H** Gene set enrichment analysis of gene sets enriched in low-dose non-responders (*n* = 12 patients). Pathway *p* values were estimated using an adaptive multilevel splitting Monte Carlo approach based on enrichment scores calculated from the ranked gene-level statistic. Benjamini-Hochberg corrected p values are displayed. **I** Boxplot of a differentially expressed gene set in non-classical monocytes. Min-max scaled expression values are displayed. Boxplot of mean CD55 gene expression per patient in non-classical monocytes. Individual cells are plotted in the background. Pie charts represent the fraction of cells with expression (expression value > 0; blue) or without expression (expression value = 0; gray). A two-sample t-test (two-sided) was applied. *n* = 18 patients. **J** Correlation of mean CD55 gene expression per patient and scaled expression of different gene sets in all patients (*n* = 27 patients). The Pearson correlation coefficient and *p* value were calculated using a two-sided Pearson correlation test. Abbreviations: UMAP uniform manifold approximation and projection, Res Responder, NonRes Non-responder, cDC conventional dendritic cells, pDC plasmacytoid dendritic cells, cl.mono classical monocytes, noncl. mono non-classical monocytes, NK cells natural killer cells, FC fold-change. Box plots display the median, first and third quartiles and whiskers are defined as 1.5 times the interquartile range.

To further delineate molecular programs underlying the generation of functional versus dysfunctional low-dose CAR-T cell products, we performed single-cell proteo-genomics on the pre-manufacturing blood from the cohort described earlier. Following rigorous quality control and data integration, this analysis yielded a total of 112,442 high-quality cells, encompassing various immune cell subsets, including different subsets of CD4 and CD8 T cells, γδ T cell subpopulations, NK cell subsets, classical, intermediate, and non-classical monocytes, and conventional dendritic cells (DCs) (Fig. 5A; Supplementary Fig. 8A, B).

Differential expression testing between cellular neighborhoods of low-dose responders and non-responders revealed various differences, particularly pronounced within the myeloid compartment (Fig. 5B). Gene set enrichment analyses and differential expression analysis indicated a marked pro-inflammatory state in the myeloid populations of low-dose responders, resembling an M1-like polarization state (Fig. 5C-G). Gene expression patterns associated with TNF signaling via NF-κB and antigen presentation machinery were significantly upregulated in the myeloid compartment of low-dose responders, consistent with M1-like polarization and a phenotype supportive of pro-inflammatory T cell activation (Fig. 5D, E; Supplementary Fig. 8C, D). In line with this, major histocompatibility complex (MHC) molecules on monocytes and dendritic cell subsets from low-dose responders were markedly upregulated at both the transcript and protein levels compared to their counterparts from low-dose non-responders (Fig. 5C, F, G).

In contrast, myeloid populations from low-dose non-responders displayed a reduced expression of their antigen presentation machinery and diminished activation of pro-inflammatory signaling pathways (Fig. 5C-G). Moreover, the myeloid compartment from non-responders was enriched in non-classical monocytes characterized by the high expression of regulators of the complement system, including CD55, clusterin (CLU) and the complement receptor CR1 (Fig. 5H, I). CD55 and clusterin expression have been previously associated with chronic inflammatory processes and suppression of tumor immunity^56,57^. Supporting a potentially regulatory role, CD55 expression correlated with pathways linked to M2-like myeloid polarization, including STAT3 and IL-10 signaling, while showing an inverse association with M1-like features, such as TNF signaling and antigen presentation (Fig. 5J). A regulatory role is further supported by elevated expression of CCR1 in non-classical monocytes (Supplementary Fig. 8E), which has been previously linked to M2-polarization^58^. Consistent with this observation, CCR1 demonstrated a similar correlation pattern to CD55, showing positive associations with M2-like polarization gene sets and negative correlations with M1-like characteristics (Supplementary Fig. 8F).

Collectively, these findings reveal that pre-manufacturing myeloid populations from low-dose responders exhibit a pro-inflammatory phenotype supportive of T cell activation, whereas myeloid cells from low-dose non-responders display a regulatory phenotype, which may negatively impact T cell activation during CAR-T cell production.

### Myeloid-derived regulatory milieu contributes to CAR-T cell dysfunction

**Fig. 6:**
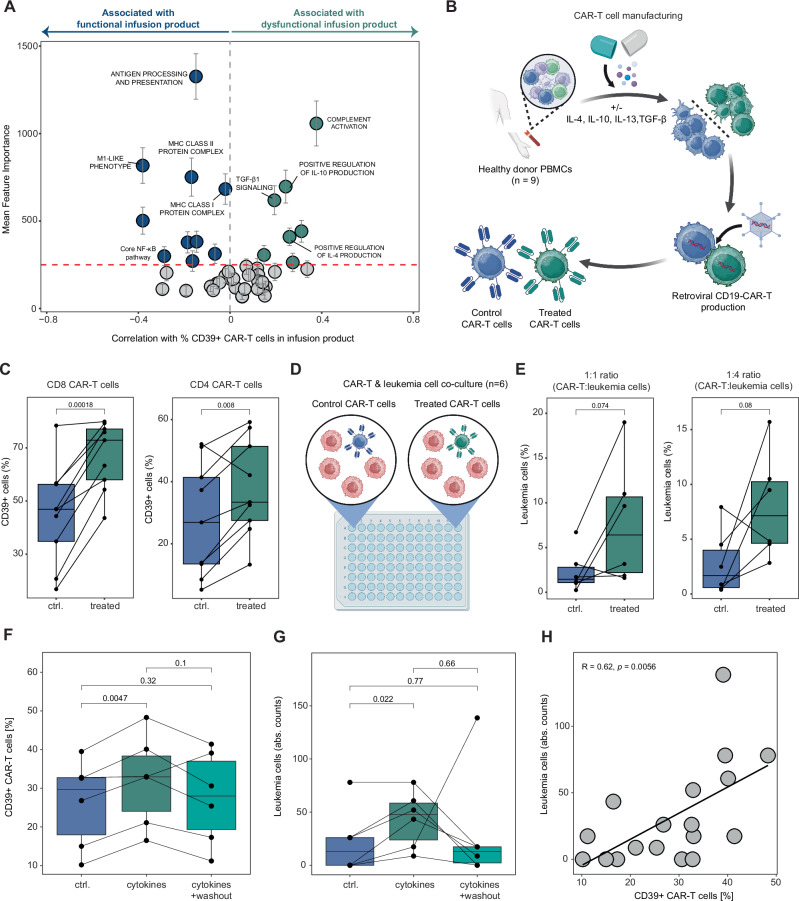
Myeloid-derived regulatory milieu contributes to CAR-T cell dysfunction. **A** Myeloid gene sets associated with a functional or CD39⁺ dysfunctional CAR-T cell product. Mean feature importance values were derived from a Random Forest analysis, averaged over 50 iterations. Error bars indicate the standard deviation (SD) across 50 iterations. **B** Schematic overview of the experimental setup for CAR-T cell manufacturing starting from healthy donor PBMCs. Created in BioRender. Röthemeier, C. (2026) https://BioRender.com/nssj5kq. **C** Frequency of CD39⁺ CD8 (left) or CD4 (right) CAR-T cells in the absence (ctrl.) or presence (treated) of cytokines. A paired t-test (two-sided) was applied. *n* = 9 healthy donors. **D** Schematic overview of the experimental setup for CAR-T cell and leukemia cell co-cultures. Created in BioRender. Röthemeier, C. (2026) https://BioRender.com/nssj5kq. **E** Frequency of live leukemia cells upon co-culturing at ratios of 1:1 (CAR-T:leukemia cells, left) or 1:4 (CAR-T: leukemia cells, right). A paired t-test (two-sided) was applied. *n* = 6 healthy donors. **F** Percentage of CD39⁺ CAR-T cells in PBMCs following cytokine pre-treatment prior to CAR-T cell manufacturing. *n* = 6 per condition. **G** Absolute number of residual Nalm6 leukemia cells after co-culture with CAR-T cells at a 1:1 effector-to-target ratio. *n* = 6 per condition. **H** Spearman correlation between the percentage of CD39⁺ CAR-T cells after cytokine pre-treatment and the absolute number of residual leukemia cells in the co-culture assay. The Spearman correlation coefficient and *p* value were calculated using a two-sided Spearman correlation test. *n* = 18. Box plots display the median, first and third quartiles and whiskers are defined as 1.5 times the interquartile range.

To evaluate whether the identified pre-manufacturing myeloid programs influence T cell states during CAR-T cell production, we analyzed potential associations between myeloid traits in pre-manufacturing blood samples and the presence of functional or CD39⁺ dysfunctional T cell states in the corresponding post-manufacturing CAR-T cell infusion products (Fig. 6A). Notably, high antigen presentation capacities and M1-polarization signatures in pre-manufacturing monocytes were positively correlated with the production of functional CAR-T cells and inversely correlated with dysfunctional CAR-T cell production. In contrast, signatures associated with TGF-β signaling and IL-4 or IL-10 production in pre-manufacturing myeloid cells, factors associated with regulatory and M2-like polarization, were linked to the production of dysfunctional CD39⁺ CAR-T cells. These data suggest that the cytokine milieu established by myeloid cells may contribute to shaping CAR-T cell states during the manufacturing phase.

To specifically assess whether regulatory polarization of myeloid cells contributes to the generation of dysfunctional CAR-T cells, we co-cultured PBMCs in the presence or absence of cytokines described to induce M2-like polarization, including IL-4, IL-10, IL-13 and TGF-β^59^. The pretreated co-cultures were then directly used as the starting material for CAR-T cell production, with no further media exchange before initiation of the manufacturing process (Fig. 6B). Consistent with our findings, supplementation with these cytokines significantly increased the generation of CD39⁺ CAR-T cells with a dysfunctional phenotype (Fig. 6C). Notably, CAR-T cells manufactured from PBMCs exposed to a milieu resembling regulatory myeloid cells, exhibited a consistent trend toward reduced targeted cell killing (Fig. 6D, E). To investigate whether these effects are transient or long lasting, we performed a washout of cytokines prior to CAR-T cell manufacturing. When cytokines were removed before manufacturing, the frequency of CD39⁺ CAR-T cells remained comparable to the untreated control, indicating that cytokine-driven upregulation of CD39 is largely transient and can be prevented by removing the myeloid-like cytokine milieu prior to CAR-T cell generation (Fig. 6F). In line with this interpretation, transient exposure to M2-like macrophages in the absence of sustained suppressive cytokine signaling did not result in a lasting impairment of CAR-T cell quality (Supplementary Fig. 9A). Consistently, CAR-T cells generated after cytokine washout displayed cytotoxic killing capacities comparable to the control (Fig. 6G). As expected, cytotoxic activity was significantly associated with the abundance of CD39⁺ CAR-T cells (Fig. 6H). Collectively, these data suggest that the cytokine milieu released by myeloid cells before and during CAR-T cell production plays a crucial yet reversible role in determining CAR-T cell functionality and cytotoxicity.

### Biomarker validation in an independent patient cohort

**Fig. 7:**
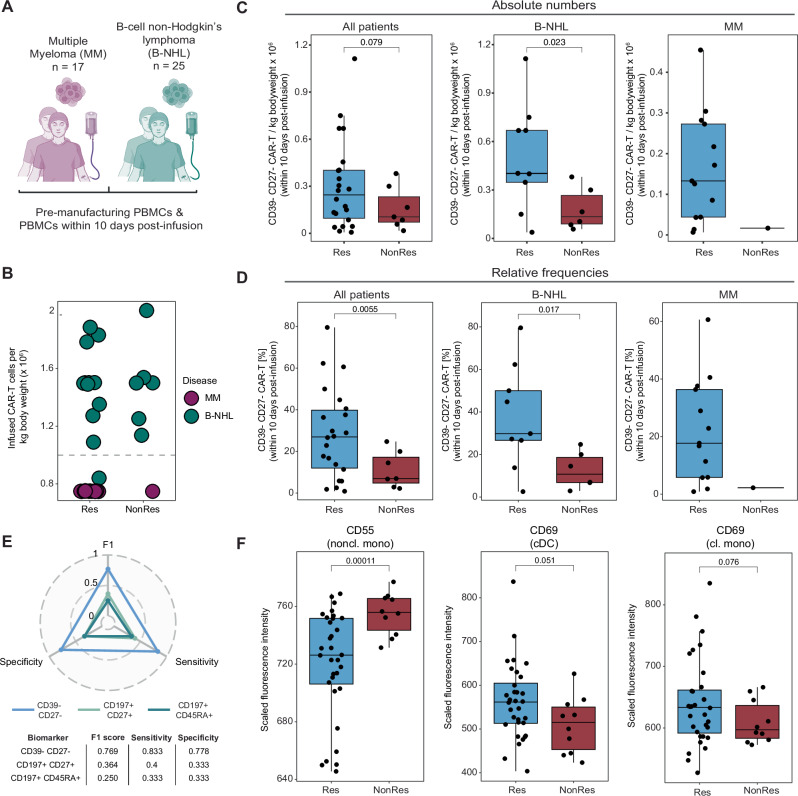
Validation of the CD27⁻CD39⁻ biomarker across an independent patient cohort. **A** Overview of patients in the validation cohort. PBMCs at the time of apheresis and CAR-T cells at a single post-infusion time point between day 1 and day 10 were analyzed. Created in BioRender. Röthemeier, C. (2026) https://BioRender.com/nssj5kq. **B** Infused number of CAR-T cells per kg body weight. The gray dashed line indicates the threshold used to define low-dose patients in the original cohort. **C** Absolute number of CD27⁻CD39⁻ CAR-T cells in the first post-infusion PBMC sample across all patients (left, *n* = 29), B-NHL (middle, *n* = 15), and MM (right, *n *= 14). *P* values were determined with a two-sided Welch’s t-test. **D** Relative frequency of CD27⁻CD39⁻ CAR-T cells in the first post-infusion PBMC sample across all patients (left, *n* = 29), B-NHL (middle, *n* = 15), and MM (right, *n* = 14). P values were determined with a two-sided Welch’s t-test. **E** Comparative evaluation of different biomarker signatures using F1-score, specificity, and sensitivity. **F** Mean fluorescence intensity of CD55 and CD69 in non-classical monocytes (noncl. mono), conventional dendritic cells (cDC), and classical monocytes (cl. mono). *P* values were determined with a two-sided Welch’s t-test (*n* = 42). Abbreviations: kg kilogram, B-NHL B-cell non-Hodgkin’s lymphoma, MM multiple myeloma, noncl. mono non-classical monocytes, cl. mono classical monocytes, cDC conventional dendritic cell. Box plots display the median, first and third quartiles and whiskers are defined as 1.5 times the interquartile range.

To assess the robustness and generalizability of our proposed biomarker combination beyond our initial cohort and CAR design, we next evaluated its association with clinical response in an independent patient cohort (*n* = 42) spanning multiple diseases (B-cell non-Hodgkin’s lymphoma [B-NHL; *n* = 25] and multiple myeloma [MM; *n* = 17]), CAR constructs (second-generation CARs with either CD28 or 4-1BB co-stimulatory domains), and targets (anti-CD19, anti-BCMA) (Fig. 7A). Patients in this validation cohort were treated with four different commercial CAR-T cell products: Breyanzi (anti-CD19, Bristol Myers Squibb), Yescarta (anti-CD19, Kite/Gilead Sciences), Kymriah (anti-CD19, Novartis), and Carvykti (anti-BCMA, Janssen Biotech/Legend Biotech).

Notably, unlike our dose-escalation study using in-house generated CAR-T cells, commercially approved anti-CD19 CAR-T cell products were infused at comparably high doses (Fig. 7B). In contrast, MM patients treated with anti-BCMA CAR-T cells received uniformly low input doses, as dictated by product specifications (Fig. 7B). For regulatory reasons, biomarker profiling of commercial products was performed on the earliest available post-infusion peripheral blood sample (day 1–10), which served as a proxy for the infused product. Biomarkers in pre-manufacturing PBMCs were assessed as in the discovery cohort.

Consistent with the findings from the discovery cohort, the CD27⁻CD39⁻ biomarker combination stratified responders from non-responders in B-NHL when quantified as absolute CD27⁻CD39⁻ CAR-T cell numbers (Fig. 7C). A similar degree of stratification was observed using relative frequencies, reflecting the comparable range of infused CAR-T cell doses in the investigated setting (Fig. 7D). In multiple myeloma, all but one patient responded to anti-BCMA CAR-T therapy. Notably, the single non-responder ranked among the lowest in both absolute counts and relative frequencies of CD27⁻CD39⁻ CAR-T cells, suggesting the biomarker’s potential applicability in this setting, although higher numbers of non-responders will need to be investigated for confirmation (Fig. 7C, D). As described in the discovery cohort, CD27⁻CD39⁻ CAR-T cells outperformed previously described biomarkers in the context of CD19-direct CAR-T therapies, in both sensitivity and specificity, when using absolute numbers (Fig. 7E). Finally, we validated the myeloid-associated biomarkers CD55 and CD69 in leukapheresis samples (Fig. 7F). As in the discovery cohort, CD55 was significantly elevated on non-classical monocytes in non-responders, whereas CD69 was enriched on cDCs and classical monocytes in responders at the leukapheresis time point (prior to CAR-T manufacturing).

Taken together, these validation data support that the CD27⁻CD39⁻ biomarker combination is reproducibly associated with clinical response across different diseases, CAR targets, and CAR constructs, suggesting that it captures a core biological property of highly potent and therapeutically effective CAR-T cells.

## Discussion

To ensure optimal therapeutic outcomes in patients, CAR-T cells are typically administered in large quantities. Accordingly, the vast majority of research has so far focused on molecular programs in high-dose settings^55,60–68^. In this study, we present the first comprehensive molecular and cellular characterization of CAR-T cell responses across a range of dose levels. Consistent with previous studies on high-dose recipients^36,43–45,47–49,55^, we observed that high-dose responders are enriched in early-lineage CAR-T cells, including naïve and central memory (CM)-like CD4 and CD8 subsets. In contrast, low-dose responders exhibited an enrichment in functional effector-memory and effector-like CAR-T cell populations. Previous studies suggest that differentiated effector-like CAR-T cells mediate initial tumor killing, while long-term responses depend on the expansion of less-differentiated subsets^43–45,50,51^. Based on these findings, we hypothesize that in most patients receiving CAR-T cells at high doses, a sufficient pool of effector-like CAR-T cells mediates initial responses, while the number of immature CAR-T cells plays a crucial role in determining sustained remission (Hoffmann et al.^51^, in vitro data). In contrast, for patients receiving low CAR-T cell doses, the number of functional effector-memory and effector-like CAR-T cells mediating immediate and potent target cell killing represents the critical factor. This hypothesis is supported by recent studies showing enhanced serial killing capacity in functional CAR-T cells from responders^69^, an enrichment of CD8 effector-memory or effector-like CAR-T cells in responders^37,65^ and findings that infusion products from non-responders are often enriched for dysfunctional, exhausted CAR-T cells^45,48,53–55^. The functional experiments performed with sorted subsets further support that CD27⁻CD39⁻ CAR-T cells represent a stable effector-like state with high per-cell cytotoxic potency. However, CD27⁺CD39⁺ CAR-T cells also showed proliferative capacity and generated CD27⁻CD39⁻ progeny, indicating that more plastic CAR-T cell states may also contribute to tumor clearance. Thus, we interpret the CD27⁻CD39⁻ phenotype as a response-associated effector state rather than an exclusive driver of therapeutic efficacy. Future in vivo studies will be required to elucidate the underlying mechanistic basis.

Mechanistically, our study suggests that when CAR-T cells are generated in limited quantities, the pre-manufacturing microenvironment during production plays a pivotal role in determining the therapeutic efficacy of the final product. Specifically, molecular programs within the myeloid compartment that contribute to the formation of functional or dysfunctional CAR-T cells are critical for mediating therapeutic outcomes at low doses. Proinflammatory myeloid programs, characterized by an M1-like phenotype and efficient antigen presentation, promote the effective generation of therapeutically active effector CAR-T cells. In contrast, insufficient T cell support, molecular programs associated with complement regulation and an M2-like myeloid phenotype are associated with therapy failure of generated CAR-T cells. These results align with previous reports in high-dose CAR-T cell recipients, where myeloid cells, particularly monocytes or myeloid-derived suppressor cells with reduced MHC-II levels, have been linked to reduced CAR-T cell expansion and lack of durable response^29,31,33,35,37,38,70,71^. Of note, supplementing the cellular starting material of the CAR-T cell manufacturing process with M2-promoting factors such as IL-4, IL-10, IL-13, and TGF-β favored the production of dysfunctional CAR-T cells with reduced killing activity. This finding is consistent with a recent study highlighting the role of IL-4 in CAR-T cell exhaustion^72^. Importantly, removal of these M2-promoting cytokines prior to CAR-T cell generation prevents the development of a dysfunctional CAR-T cell phenotype. These findings suggest that regulatory myeloid-associated signals impair CAR-T cell function through a reversible, manufacturing-dependent mechanism rather than a fixed, patient-intrinsic determinant. Consistently, transient exposure to M2-like macrophages alone did not durably impair CAR-T cell quality. The validation data in a cross-disease and independent patient cohort further supports the role of myeloid M1/M2 programs that contribute to the functionality or dysfunctionality of the CAR-T cell product. Because of legal constraints prohibiting direct evaluation of biomarkers in commercial infusion products, the validation cohort relied on CAR-T cells in peripheral blood at early time points post-infusion as a proxy for the initial CAR-T infusion product. While we acknowledge that this analysis did not directly assess the actual infusion product, these data nevertheless support the association between the CD27⁻CD39⁻ phenotype, early on-treatment CAR-T cell behavior and clinical response. Jointly, these observations could form the basis for strategies to improve CAR-T cell functionality during the manufacturing process and enhance effective therapy at low doses.

Our findings carry significant clinical implications. For a considerable proportion of patients eligible for CAR-T cell therapy, the number of manufactured CAR-T cells may not meet current end-product specifications, thus resulting in an OOS product. As a result, these patients may not receive treatment, or CAR-T cells may only be administered at the discretion of the treating physician, leading to uncertain therapeutic outcomes. This challenge is underscored by recent reports showing that, although CAR-T cell products can be generated with markedly shortened manufacturing processes of approximately 2–3 days^73,74^, these accelerated protocols often fail to produce sufficient cell numbers within this timeframe. This, in particular, can exacerbate the risk of OOS for already challenging patient samples and clinical situations. On the other hand, pharmaceutical enterprises manufacturing OOS products face the challenge of non-reimbursement, which introduces major financial risks. These risks are often offset by inflated costs for functional CAR-T cell products, ultimately contributing to the high overall treatment expenses. In addition, current biomarker strategies for predicting response have been primarily developed for patients receiving CAR-T cells in excess. Importantly, our study suggests that these strategies may not be applicable to low-dose recipients due to two distinct conceptual differences. First, while relative frequencies can be particularly useful when comparing patients with comparable CAR-T cell doses, the absolute counts of CAR-T cells are more predictive of therapeutic response, when comparing patients receiving different dose levels. Second, the number of non-dysfunctional effector- and effector-memory-like CAR-T cells is a more powerful predictor of response in low-dose patients. In light of these findings, we propose a simple biomarker strategy (CD27⁻CD39⁻) to assess a minimum threshold of therapeutically active CAR-T cells necessary to elicit a response at limiting doses, applicable in CD19-directed CAR-T therapies of the second and third generation, and likely transferable to BCMA-directed CAR-T cells. These conceptual advances may offer a basis for predicting therapy response in OOS CAR-T cell products or other settings, such as in accelerated manufacturing protocols, where target cell numbers are not met. This could expand access to CAR-T cell therapy for a broader range of patients, reduce costs associated with therapy failures, and potentially lead to revisions in regulatory standards for CAR-T cell production.

## Methods

### Study design and study identification

The samples analyzed in this study are derived from the HD-CAR-1 trial: NCT03676504 (clinicaltrials.gov), EudraCT-No. 2016-004808-60, federal authority No.: 3148/02, Institutional review board/Ethics Committee approval (Heidelberg University) No.: AF-mu 405/2017. Further details on the HD-CAR-1 clinical trial design and patient inclusion criteria are available in the original study protocol, which includes ALL, CLL, and B-NHL cohorts^52^. Importantly, this original protocol specified three planned dose levels only (DL1: 1 × 10^6^, DL2: 5 × 10^6^, and DL3: 20 × 10^6^ CAR-T cells/m²). The trial was later amended to include three additional dose levels (DL4: 50 × 10^6^, DL5: 100 × 10^6^, and DL6: 200 × 10^6^ CAR-T cells/m²), as reported in Schubert et al.^75^ and described in detail in the clinical trial study record NCT03676504. Clinical data for the ALL and CLL cohorts have been reported separately in Schubert et al.^53^ and Derigs et al.^54^. Characteristics of all patients included in this study are summarized in Supplementary Data 1. The study profile of the HD-CAR-1 cohort, including the exact administered dose levels, is shown in Supplementary Fig. 10. Clinical efficacy of HD-CAR-1 treatment was assessed at end-of-study (EOS), i.e., day 90 (d90) after CAR-T cell treatment, based on the response criteria defined for ALL^76^, chronic lymphocytic leukemia (CLL)^77^ and NHL^78^. For the B-NHL cohort, clinical efficacy and outcome data are presented in Supplementary Fig. 11. Corresponding clinical metadata, including response status at EOS and treatment-related adverse events such as cytokine release syndrome (CRS) and immune effector cell-associated neurotoxicity syndrome (ICANS), as well as dose category and exact administered dose for each patient, are provided in Supplementary Data 1. Dose levels in the study protocol were defined as CAR-T cells/m². For each patient, the assigned dose level was multiplied by the individual body surface area (BSA) to calculate the absolute number of CAR-T cells to be infused. BSA was calculated from body weight (kg) and height (cm) using the formulas of Mosteller^79^ and Du Bois^80^. The average of both calculations was used for each patient, respectively. To allow comparison with previous studies reporting the CAR-T cell dose normalized to kilogram of body weight, the absolute number of infused CAR-T cells was divided by the patient’s body weight (kg) and indicated as CAR-T cells per kilogram of body weight. Patients were classified as responders and non-responders as follows: for ALL, only patients negative for minimal residual disease (MRD) were considered responders, whereas those with molecular relapse after CAR-T cell treatment, i.e., MRD positivity at EOS (detectable MRD > 1 × 10^−4^), were considered non-responders. For the CLL/NHL cohort, responders had achieved a complete response (CR) or partial response (PR) at EOS, while non-responders had stable disease (SD) or experienced disease progression after treatment. Patients receiving >1 × 10^6^ infused CAR-T cells per kilogram of body weight were defined as high-dose recipients, while patients receiving <1 × 10^6^ infused CAR-T cells per kilogram of body weight were defined as low-dose recipients.

### Ethics approval

The HD-CAR-1 clinical trial was conducted in accordance with the principles outlined in the Declaration of Helsinki (2008). All participants provided written informed consent, and their confidentiality and anonymity were ensured in compliance with German general national regulations, specifically the Bundesdatenschutzgesetz (BDSG). Ethical approval, along with approvals from both local and federal competent authorities, was obtained. The trial also received Institutional Review Board/Ethics Committee approval from Heidelberg University (No.: AF-mu 405/2017). PBMC samples from healthy blood donors were obtained as buffy coats from the blood donation center 'Zentrum für Transfusionsmedizin und Zelltherapie Berlin' (ZTB) Berlin. All analyses of blood samples from healthy donors were conducted in accordance with the Declaration of Helsinki and local ethical guidelines. The acquisition and use of healthy donor buffy coats were approved by the Institutional Review Board/Ethics Committee of Charité—Universitätsmedizin Berlin. Approval for the recruitment of voluntary healthy blood donors was obtained from Ethikausschuss 1 am Campus Charité—Mitte under approval numbers EA1/003/17 and EA1/222/13. The use of anonymized healthy donor buffy coats was covered by the ethics approvals listed above, including any applicable consent requirements or waivers.

### Full-spectrum flow cytometry of CAR-T cell infusion products

CAR-T cell infusion products from 27 patients enrolled in the HD-CAR-1 trial were analyzed on the same day with full-spectrum flow cytometry (Cytek Aurora 5 L) using a 36-marker panel. The infusion product of P2-19 was unavailable on the day of measurement. Samples were thawed at 37 °C, washed and cell suspensions resuspended in 2% fetal calf serum (FCS) 0.5 mM EDTA PBS (FACS buffer) for performing staining with the antibody mix. Staining was carried out in three consecutive rounds, each involving a 20-min incubation at 4 °C. Antibodies used are summarized in Supplementary Table 2.

### Full-spectrum flow cytometry of pre-manufacturing PBMCs

Pre-manufacturing PBMCs at time point of leukapheresis from 28 patients enrolled in the HD-CAR-1 trial were analyzed on two consecutive days with full spectrum flow cytometry (Cytek Aurora 5L) using a 35-marker panel. Samples were thawed at 37 °C, washed and cell suspensions resuspended in 2% FCS 0.5 mM EDTA PBS (FACS buffer) for performing staining with the antibody mix. Staining was performed at 4 °C for 30 min. Antibodies used are summarized in Supplementary Table 3.

### Full-spectrum flow cytometry data pre-processing

For full-spectrum flow cytometry data, spectral unmixing was performed using the SpectroFlow software (Cytek Biosciences, v. 3.2.1). To detect and remove anomalies based on common flow cytometry parameters, .fcs files were further processed using the R package flowAI (v. 1.24.0). The function flow_auto_qc was run, and anomalies identified based on the flow rate were removed. Obtained .fcs files were then imported into FlowJo (BD Biosciences, v. 10.8.1) to assess unmixing quality by visualizing markers in *N* × *N* plots. The data was transformed using the in-built logicle transform function of FlowJo. Populations of interest (single live cells) were exported as .csv files using channel values for downstream analysis in R (v. 4.3.0).

### Flow cytometry data downstream analyses

All downstream analyses were performed in R (v. 4.3.0). The Seurat^81^ package (v. 5.0.3) was used for downsampling, clustering, and dimensionality reduction. Instead of using all cells (on the order of 10 million) for dimensionality reduction and clustering, we applied atomic sketching, a non-uniform downsampling strategy that selects a representative subset of cells (“atoms”) based on leverage scores, which is designed to preserve the overall cellular landscape and to enrich for rare populations. These atoms from all patients were then used for joint dimensionality reduction by principal component analysis (PCA), clustering with the Louvain algorithm^82^ and data visualization in a uniform manifold approximation and projection (UMAP) embedding^83^. Manual annotation of clusters was conducted based on marker expression and expert knowledge. Cluster labels of cells not selected by sketching were determined by Linear Discriminant Analysis as implemented in the MASS^84^ R package (v. 7.3-60). Importantly, while atomic sketching was used to obtain a representative embedding for visualization and clustering, all downstream quantitative and statistical analyses were performed on the entire dataset, including all cells.

Differential abundance using DA-seq^85^ (v.1.0.0) was performed according to the DA-seq vignette with default settings. Values for the k vector were adapted and set to 20 and 500. To quantify differential abundance of cell types, the frequency or absolute numbers of each subset was determined per sample. Depending on the comparison, the frequency of each cell type per sample in one group was divided by the corresponding mean frequency of the same cell type in the other group. The obtained fold-changes (FC) were then log_2_-transformed and visualized using ggplot2^86^. To determine significance, a one-sample t-test (two-sided) was applied, and p values were corrected using Benjamini-Hochberg. For the correlation analysis log_2_-FC values were initially determined as described. For each cell type, the mean was calculated within the group of interest and correlated with the respective mean values of the comparison group. The Pearson correlation coefficient was computed for each correlation analysis.

To determine the classification performance of each cell type in the CAR-T cell product, a receiver-operating characteristic (ROC) analysis was performed. For this, the relative frequencies or absolute numbers of each cell type were calculated per sample. Then, for each cell type, a ROC curve was plotted for relative frequencies and absolute numbers, respectively. The optimal cutoff was identified using the “closest to top left” approach. For the subset of CD39⁺ CM-like CD4 CAR-T cells within low-dose patients, the cutoff could not be identified, therefore this subset was excluded from the analysis of low-dose recipients. The samples were then classified into binary categories (responder or non-responder) based on the determined cutoff, and the predicted labels were compared to the true response labels. From this confusion matrix, precision and recall were extracted and the F1 score was calculated. To assess the classification performance of biomarkers, cells were first gated based on the expression levels of the respective surface markers. Then, the relative frequencies or absolute numbers of biomarker associated cells was calculated per sample followed by the same approach as outlined above.

### Statistical analysis of covariates associated with clinical response

Associations between clinical/biological covariates and clinical response were assessed using univariable logistic regression, with clinical response (Responder vs. Non-Responder) as the binary outcome and each covariate as a separate predictor (Supplementary Table S2, Supplementary Fig. 1, Supplementary Fig. 4). Odds ratios (ORs) and 95% confidence intervals (CIs) were estimated for each model. Because of the limited cohort size and sparse data in some categories, we used a stepwise strategy to obtain stable estimates. First, a standard logistic regression model was run. If the model failed due to separation (i.e., one outcome occurring only in one group), Firth’s penalized logistic regression was applied, which is an approach specifically designed for small sample sizes. If this was still not feasible, ORs and 95% CIs were derived from 2 × 2 tables using a Haldane-Anscombe continuity correction (0.5 added to each cell). *P* values were obtained from Fisher’s exact tests and adjusted for multiple testing. To further assess the relationship of numeric covariates, Pearson correlation coefficients were calculated. Associations among categorical covariates were determined by using contingency tables and summarized by Cramér’s V (range 0–1), which yields a matrix of association strengths.

### Single-cell proteo-genomics

Pre-manufacturing PBMCs from 28 patients enrolled in the HD-CAR-1 trial were subjected to single-cell proteogenomics analysis using the BD Rhapsody platform on two consecutive days. In brief, samples were thawed at 37 °C, washed and cell suspensions were resuspended in 2% FSC 0.5 mM EDTA PBS (FACS buffer). Cells were stained with a live-dead mix including DAPI and Caspase-3. After a short incubation with the live-dead mix at 4 °C, 25k T cells and 25k non-T cells were sorted for each sample using a FACSAria III sorter (BD Biosciences). The sorted cells were then pooled for each sample, respectively, prior to labeling with hashing (sample-tag) antibodies according to the manufacturer’s instructions. Incubation with hashing antibodies was performed on ice for 30 minutes. After three rounds of washing, samples were stained with the Abseq master mix (83 oligonucleotide-labeled antibodies; from BD Biosciences) for 30 min on ice. Abseq antibodies used are summarized in Supplementary Table 4. Subsequently, samples were washed three times before continuing with the single-cell capture protocol using the BD Rhapsody scRNA-seq platform (BD Biosciences). Single-cell capture and preparation of hashing (sample-tag), mRNA and Abseq libraries were performed according to the manufacturer’s instructions. Quality control of libraries was conducted using the Qubit dsDNA HS (High Sensitivity) kit and the 4200 TapeStation system (Agilent). Pooled libraries were sequenced using the Novaseq 6000 system (Illumina).

### Pre-processing of single-cell proteo-genomics data

Fastq files were pre-processed using the BD Rhapsody pipeline (v. 2.0) according to the manufacturer’s recommendations and instructions. The obtained unique molecular identifier (UMI) count matrices were imported into R (v. 4.3.0) for further processing and quality control prior to downstream analyses using the Seurat framework (v. 5.0.3). For one pooled library, the inflection point was determined manually due to challenges with the automated detection of the inflection point (true cell barcodes). The manually defined inflection point was comparable to that of the other pooled libraries. High-quality cells used for downstream analyses were determined based on stringent quality control measures, including the total number of UMIs per cell (>1000), the total number of features per cell (>1000) and the frequency of mitochondrial genes (<25%). Furthermore, only features present in at least three cells were retained.

### Downstream analyses of single-cell proteo-genomics data

To account for differences in sequencing depth, UMI counts of the RNA assay were log-normalized while counts of the Abseq assay were normalized using the centered log ratio (CLR). Subsequently, the normalized count matrices from both assays were concatenated and batch integration was performed using Seurat’s CCA integration method. Following dimensional reduction using PCA, Louvain clustering and data visualization in a UMAP embedding, clusters were annotated manually based on expert knowledge using gene and Abseq marker expression. To identify cellular neighborhoods with differentially expressed genes between low-dose responders and non-responders within the pre-manufacturing blood, miloDE^87^ (v. 0.0.09) was applied according to the vignette with default settings. Prior to differential expression testing, neighborhoods without any perturbation were discarded based on an AUC cutoff <0.65. Neighborhoods with >2 differentially expressed genes were highlighted in the cellular neighborhood UMAP embedding. For differential gene expression analysis of myeloid cells within low-dose patients, DESeq2 was applied using the light wrapper from the DElegate R package (v. 1.2.1). Mitochondrial and ribosomal genes were excluded for this analysis. For genes with adjusted rounded p-values of 0, this value was replaced with an extremely small pseudo-value (1 × 10⁻³⁰⁰) to avoid issues with calculating −log₁₀(*p*_adj_). Gene set enrichment analysis (GSEA) was performed for classical and non-classical monocytes of low-dose patients, independently. Initially, differentially expressed genes were determined using Seurat’s FindMarker function and ranked according to their log_2_-FC. Pathways and categories were selected using the Molecular Signatures Database (MSigDB) as implemented in the msigdbr^88,89^ R package (v. 7.5.1). A multi-level GSEA analysis was performed on the ranked genes using the fgseaMultilevel function as implemented in the fgsea^90^ R package (v. 1.26.0). For plotting of pathways, the plotEnrichment function was used. To evaluate whether pre-manufacturing myeloid programs (gene sets) influence T cell states during CAR-T cell production, a Random Forest model was trained iteratively 50 times using the R package randomForest (v. 4.7-4.11). The mean feature importance and standard deviation across all iterations were calculated for each feature. To investigate the relationship between myeloid gene sets and CAR-T cell states, the Pearson correlation coefficients were computed.

### Biomarker analysis in the validation cohort

Patient samples of the validation cohort were obtained after written informed consent in accordance with the Declaration of Helsinki. The study was approved by the institutional ethics committee of the Charité Universitätsmedizin Berlin (Ethics approval number EA2/142/20). Clinical efficacy for this cohort was assessed at day 180 after CAR-T cell treatment. Characteristics of patients included in this cohort are summarized in Supplementary Data 2. CAR-T cell (CD27 and CD39) and myeloid cell (CD55 and CD69) biomarker expression was assessed by flow cytometry in an independent patient cohort (*n* = 42). This cohort included patients with multiple myeloma (*n* = 17) and B-cell non-Hodgkin’s lymphoma (*n* = 25). Leukapheresis samples were available for all patients and were analyzed by flow cytometry for CD55 and CD69 expression on non-classical monocytes, classical monocytes, and conventional dendritic cells. For 29 patients, an early post-infusion PBMC sample (day 1–10) was available and used to assess CD27 and CD39 expression on CAR-T cells in vivo. Antibodies used are summarized in Supplementary Table 5.

### PBMC isolation and cytokine treatment

PBMCs were isolated from the peripheral blood of nine healthy donors (HD) by density gradient centrifugation. Cell concentration was adjusted at 1 × 10^6^/ml in complete medium (45% RPMI-1640 (21875091, Gibco Invitrogen), 45% Click’s medium (9195 FujiFilm; Irvine Scientific, Santa Ana, CA), 2 mM l-glutamine (25030081, ThermoFisher), and 10% fetal bovine serum (A5256801, Gibco) supplemented with 10 ng/mL interleukin-7 (IL-7) (207-IL-025, Peprotech, Rocky Hill, NJ) and 5 ng/mL IL-15 (247-ILB-025, Peprotech, Rocky Hill, NJ)) and plated in a 24-well plate. PBMCs were then treated with cytokine mixtures consisting of IL-4 (200-04, PeproTech, Inc), IL-10 (200-10, PeproTech, Inc), IL-13 (200-13, PeproTech, Inc) and TGF-β (100-21, PeproTech, Inc) at a final concentration of 20 ng/mL each. Plates were incubated at 37 °C with 5% CO₂ for 16 h. For cytokine washout experiments, the same experimental setup was prepared with one additional condition in which cytokines were added, as previously described, but then subjected to extensive washing before CAR-T cell manufacturing.

### Retroviral CD19-CAR-T cell production

Retroviral vector production and T cell transduction were performed as previously described^53,91,92^. Briefly, 293T cells (ACC 635, DSMZ) were transfected with SFG vector carrying CD19-CAR construct, in addition to the packaging plasmids (PeqPam, RD114), with GeneJuice (Merck Millipore, Billerica, MA). The viral supernatant was harvested after 48 and 72 h. Following cytokine treatment, T cells were activated by OKT3 (317326, Biolegend) and CD28 (302934, BioLegend) antibodies and expanded for 48 h in complete medium, before retroviral transduction on day 3 using RetroNectin (T100B, Takara).

### Co-culture assays

After 12 days, CAR-T or nontransduced (NT) T cells were analyzed by flow cytometry and co-cultured with the leukemia cell line Nalm6-zsGreen (ACC 128, DSMZ) in 96-well plates in the absence of exogenous cytokines. Target cells were co-cultured at effector-to-target (E:T) ratios of 1:1 and 1:4 for 72 h, following analysis by flow cytometry.

### Flow cytometry of cytokine and co-culture assays

Flow cytometry was performed on days −1, 12 and 15. Cells were stained using the following anti-human antibody panel: CD3, CD4, CD8, CD39, CD33, and CD16 (summarized in Supplementary Table 6). Antibodies were purchased from BD Biosciences (San Jose, CA), Beckman Coulter (Brea, CA), Thermo Fisher Scientific (Waltham, MA), and Biolegend (San Diego, CA). For the detection of CD19-CAR-T cells, a biotinylated protein L (Thermo Fisher Scientific) followed by fluorochrome-labeled streptavidin (BD Biosciences) was used. Data were acquired using the FACSCanto II and FACSymphony A1 (BD Biosciences).

### Analysis of flow cytometry data from cytokine and co-culture assay

Flow cytometry data were analyzed with FlowJo (v. 10, Tree Star, Ashland, OR). For the cytokine assay and the analysis of CD39-expressing cells, live, single cells were selected. Within CD4 and CD8 T cells, CAR-expressing cells were selected and the fraction of CD39⁺ cells within the respective T cell compartment was determined.

For the co-culture assay, the fraction and absolute number of live Nalm6 leukemia cells were determined based on the expression of Nalm6 and 7AAD.

### Functional validation of CD27⁻CD39⁻ CAR-T cells

For functional validation of the CD27⁻CD39⁻ CAR-T cell phenotype, CAR-T cells were generated from healthy donor PBMCs. After manufacturing, CAR-T cells were sorted into two populations: (1) CD27⁻CD39⁻ and (2) CD27⁺CD39⁺ (as control population). Both populations were co-cultured for two consecutive 72-h rounds with Nalm6 leukemia cells at a 1:1 effector-to-target ratio. After each co-culture round, cells were stained with the following antibody panel and analyzed by flow cytometry: CD3, CD4, CD8, CD27, CD39, and an anti-CAR antibody. Nalm6 cells were GFP-labeled to allow discrimination from CAR-T cells. Counting beads was added to determine absolute cell numbers. To quantify per-CAR-T cell killing capacity at 72 h, both the absolute abundance of CAR-T cells at 72 h and leukemia cell expansion were incorporated into the analysis. For each donor and sorted CAR-T cell subset, the absolute CAR-T cell count was determined to obtain the total number of CAR+ cells present at 72 h. Non-transduced (NT) samples were used as donor-matched leukemia-growth controls. For multiplex cytokine profiling, 50 µl of co-culture supernatant was collected per sample. Cytokine levels were quantified using the Olink Target 48 Cytokine panel according to the manufacturer’s instructions.

### Reporting summary

Further information on research design is available in the Nature Portfolio Reporting Summary linked to this article.

## Supplementary information

Supplementary Information

Description of Additional Supplementary Files

Supplementary Data 1

Supplementary Data 2

Reporting Summary

Transparent Peer Review file

## Source data

Source Data

## Data Availability

High-quality flow cytometry data, single-cell proteo-genomics data and clinical metadata are provided under the following link: 10.5281/zenodo.14711865. The sequencing data generated in this study have been deposited in the NCBI Gene Expression Omnibus (GEO) under accession number GSE314571. Source data are provided with this paper.

## Code availability

R scripts and code are provided under the following link: 10.5281/zenodo.14711865.
