# Supplementary material for: A distinct CAR-T cell phenotype mediates therapeutic response at limited doses
Source: Nat Commun. 2026 Jul 29;17:7589. doi: 10.1038/s41467-026-76068-4 (PMC13421456; doi:10.1038/s41467-026-76068-4)
Supplement: Supplementary file 1 — Supplementary Information [file 41467_2026_76068_MOESM1_ESM.pdf]

A Distinct CAR-T Cell Phenotype Mediates Therapeutic Response at Limited Doses

AUTHORS

Schayan Yousefian[1-3], Maria-Luisa Schubert[4], Anna Rita Minafra[4], Patrick Derigs[4], Sarah Gräßle[1-3,5], Arik Horne[1-3,6-8], Uta M. Demel[3,9,10], Julian Liebaert[3], Caroline Röthemeier[1-3], Franziska Pupp[11], Uta E. Höpken[11], Jan Krönke[3,8,12], Antonia Busse[3,8,9], Ulrich Keller[3,8,9,13], Anita Schmitt[4], Daniel Hübschmann[6,7,14,15], Carsten Müller-Tidow[4,14,16], Peter Dreger[4], Michael Schmitt[4,14,16] §, Simon Haas[1-3,8,13,17] §.

[1] Berlin Institute of Health (BIH) at Charité Universitätsmedizin Berlin, Berlin, Germany
[2] Berlin Institute for Medical Systems Biology, Max Delbrück Center for Molecular Medicine in the Helmholtz Association, Berlin, Germany
[3] Charité – Universitätsmedizin Berlin, corporate member of Freie Universität Berlin and Humboldt-Universität zu Berlin, Department of Hematology, Oncology and Tumor Immunology, Berlin, Germany
[4] Internal Medicine V, Hematology, Oncology and Rheumatology, Heidelberg University Hospital, Heidelberg, Germany
[5] Humboldt-Universität zu Berlin, Institute of Biology, Berlin, Germany
[6] Computational Oncology Group, Molecular Precision Oncology Program, National Center for Tumor Diseases (NCT) Heidelberg and German Cancer Research Center, Heidelberg, Germany
[7] Innovation and Service Unit for Bioinformatics and Precision Medicine (BPM), German Cancer Research Center, Heidelberg, Germany
[8] German Cancer Consortium (DKTK), Partner Site Berlin, Berlin, Germany
[9] Max-Delbrück-Center for Molecular Medicine, Berlin, Germany
[10] Clinician Scientist Program, Berlin Institute of Health (BIH), Berlin, Germany
[11] Max Delbrück Center for Molecular Medicine, Department of Microenvironmental Regulation in Autoimmunity and Cancer, Berlin, Germany
[12] Internal Medicine C, Hematology, Oncology, Stem Cell Transplantation and Palliative Care, University Medicine Greifswald, Greifswald, Germany
[13] Cluster of Excellence ImmunoPreCept, Charité - Universitätsmedizin Berlin, Berlin, Germany
[14] German Cancer Consortium (DKTK), Partner Site Heidelberg, Heidelberg, Germany
[15] Division of Translational Precision Medicine, Institute of Human Genetics, Heidelberg University, Heidelberg, Germany
[16] National Center for Tumor Diseases (NCT), Heidelberg, Germany
[17] Precision Healthcare University Research Institute, Queen Mary University of London, London, UK

§ shared senior authors
Correspondence should be addressed to Michael.Schmitt@med.uni-heidelberg.de or simon.haas@bih-charite.de

SUPPLEMENTARY FIGURES

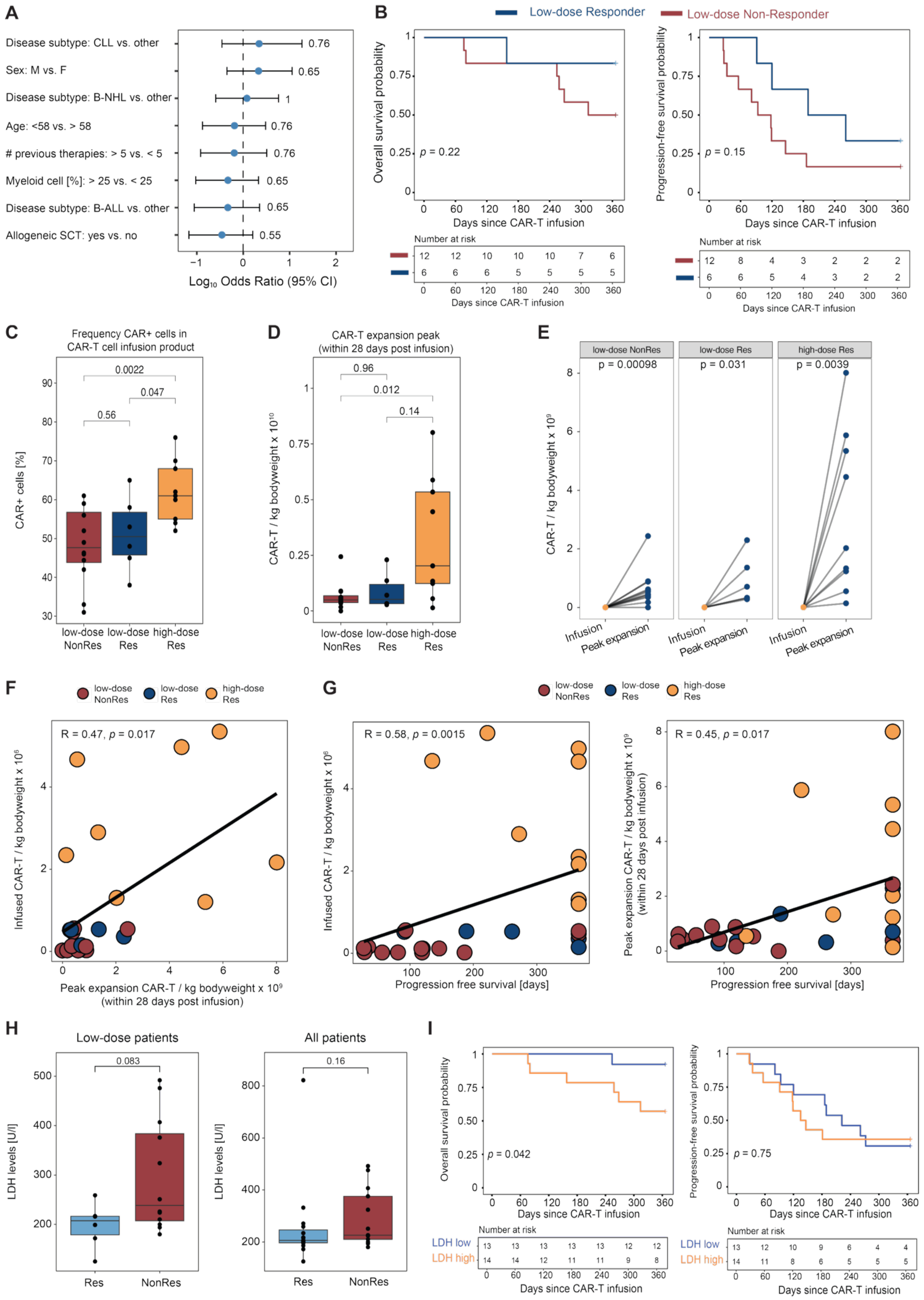

Supplementary Figure 1. Confounding factors, long term outcomes and levels of tumor burden. A. Forest plot with odds ratios for the association between clinical response and clinical or biological covariates. P values were obtained from Fisher's exact tests and adjusted for multiple comparisons using the Benjamini-Hochberg correction. The 95% confidence intervals are displayed. **B.** Kaplan-Meier curves for low-dose patients showing overall survival (OS, top) and progression-free survival (PFS, bottom). P values were computed using the log-rank test. n = 18 patients. **C.** Frequency of CAR+ cells in the infusion product measured by flow cytometry. P values were determined with a two-sided Welch's t-test. n = 27 patients. **D.** Absolute number of CAR-T cells per kg body weight at peak expansion within 28 days post-infusion, quantified by quantitative real-time PCR (qPCR) for the CAR transgene. P values were determined with a two-sided Wilcoxon rank-sum test. n = 27 patients. **E.** Paired comparison of the absolute number of CAR-T cells per kg body weight at infusion versus peak expansion. P values were determined with a paired, two-sided Wilcoxon rank-sum test. n = 27 patients. **F.** Spearman correlation between the absolute number of infused CAR-T cells per kg body weight and the absolute number of CAR-T cells per kg body weight at peak expansion. n = 26 patients. **G.** Spearman correlation between progression-free survival (days) and the absolute number of infused CAR-T cells per kg body weight (left) and the absolute number of CAR-T cells per kg body weight at peak expansion (right). n = 27 patients. **H.** Lactate dehydrogenase (Com) levels as a proxy for tumor burden in low-dose patients (top, n = 18) and in all patients from the original cohort (bottom, n = 28). **I.** Kaplan-Meier curves for all patients stratified by LDH level, showing overall survival (OS, top) and progression-free survival (PFS, bottom). P values were computed using the log-rank test. n = 27 patients. Abbreviations: kg = kilogram, LDH = Lactate dehydrogenase. Box plots display the median, first and third quartiles and whiskers are defined as 1.5 times interquartile range.

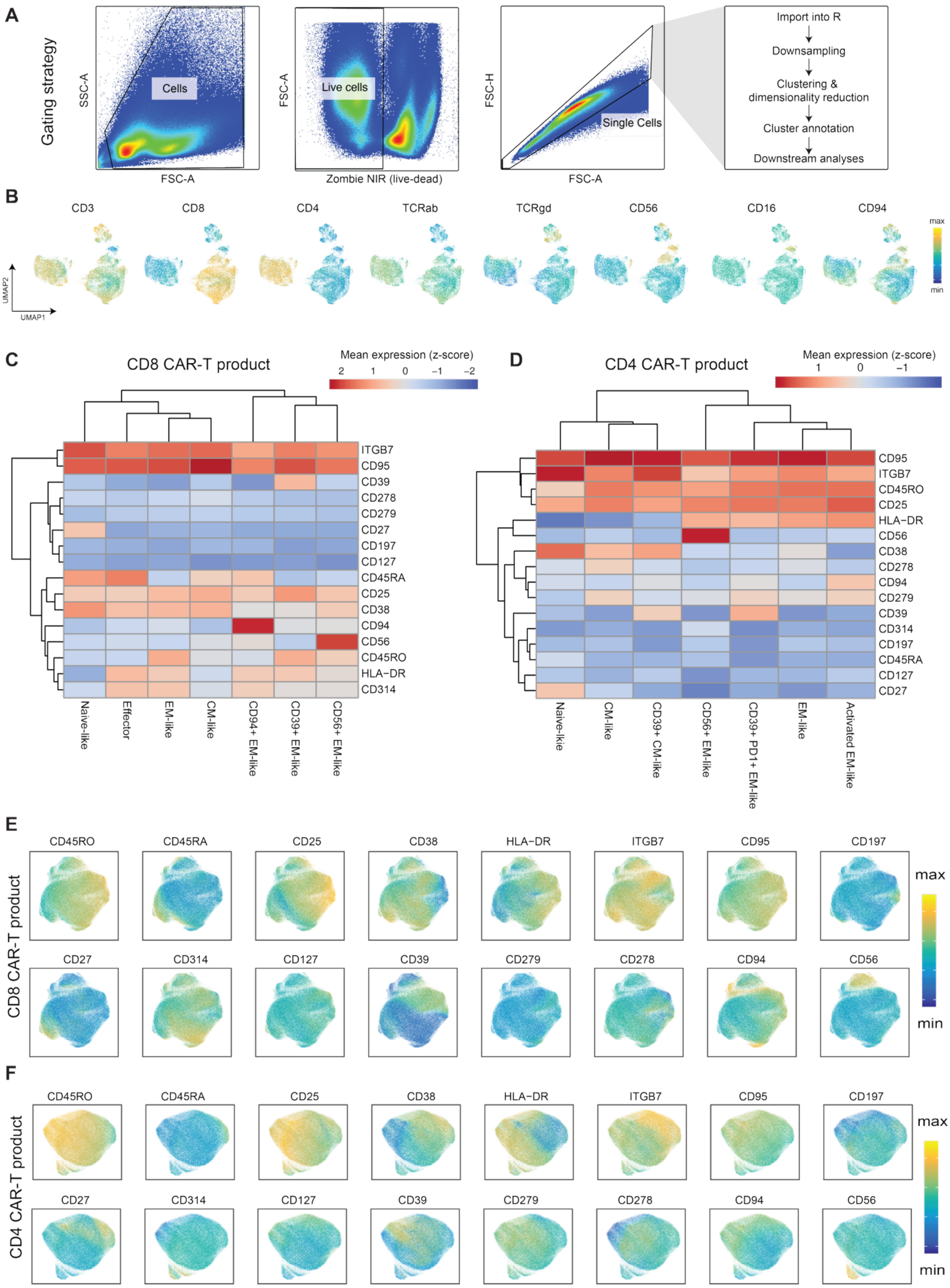

Supplementary Figure 2. Gating strategy and surface marker expression for cluster annotation of CAR-T cells products. A. Gating strategy and downstream workflow for flow cytometry data analysis. **B.** Feature plots highlighting the expression of cell type specific markers within the overview UMAP of the CAR-T cell infusion products (n = 27). **C.** Heatmap showing z-scored mean expression of T cell markers used for annotation of CD8 CAR-T cell clusters. **D.** Heatmap showing z-scored mean expression of T cell markers used for annotation

of CD4 CAR-T cell clusters. **E.** Feature plots showing expression intensities of T cell markers in CD8 CAR-T cells. **F.** Feature plots showing expression intensities of T cell markers in CD4 CAR-T cells.

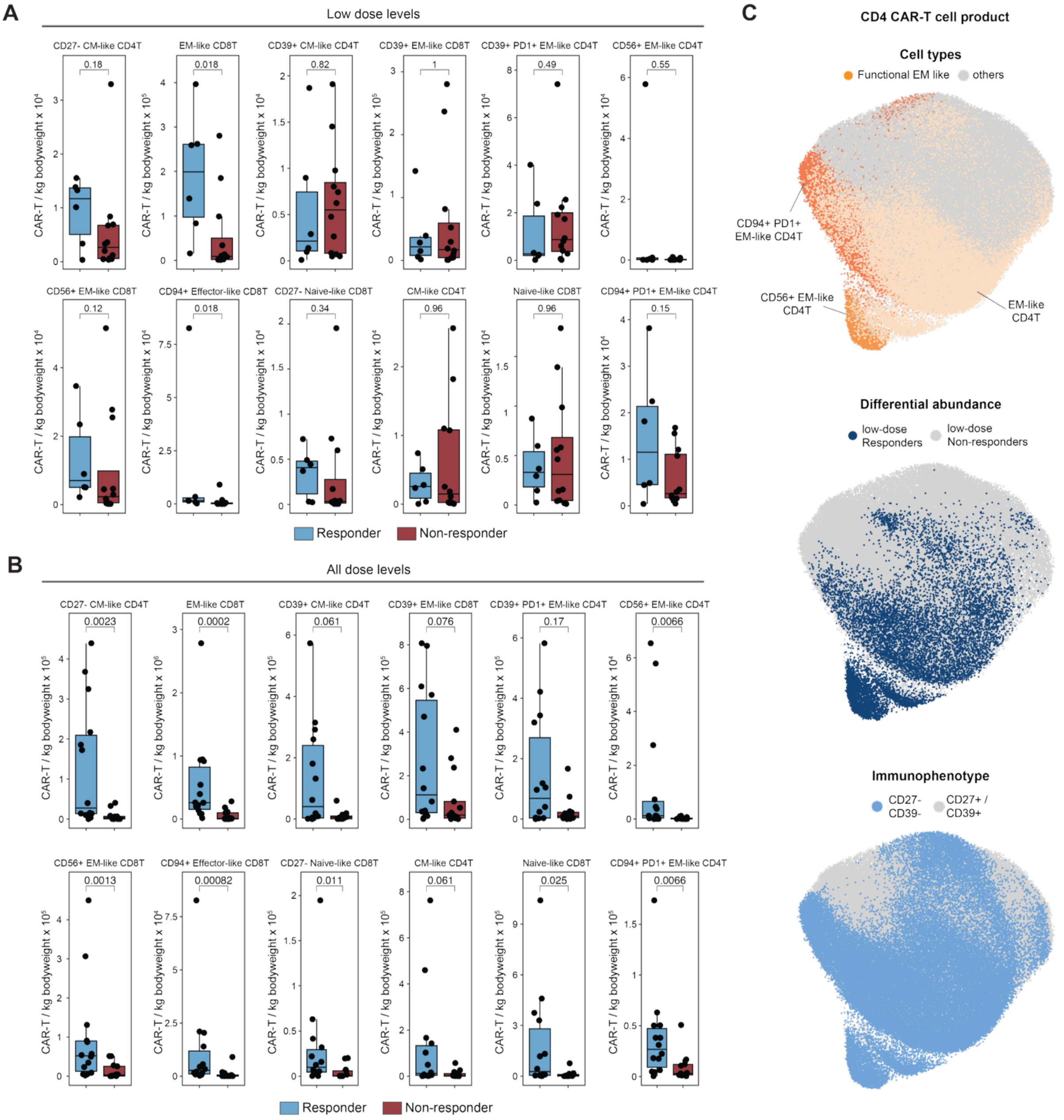

Supplementary Figure 3. Absolute number of functional effector-like CAR-T cells are crucial for therapy response. A. Boxplots comparing the absolute number of infused CAR-T cell subsets per kg of body weight between low-dose responders (n = 6) and low-dose non-responders (n = 12). A Wilcoxon rank-sum test was applied. **B.** Boxplots comparing the absolute number of infused CAR-T cell subsets per kg of body weight within all responders (n = 14) and non-responders (n = 13). A Wilcoxon rank-sum test was applied. **C.** UMAPs of the CD4 CAR-T cells of the infusion product (n = 27). Top panel: Functional effector or effector memory-like populations are highlighted in orange. Middle panel: Differential abundance comparing low-dose responders to low-dose non-responders. Bottom panel: Highlighted immunophenotype based on absence of CD27 and CD39 expression. Abbreviations: EM = effector-memory, CM = central-memory, kg = kilogram. Box plots display the median, first and third quartiles and whiskers are defined as 1.5 times interquartile range.

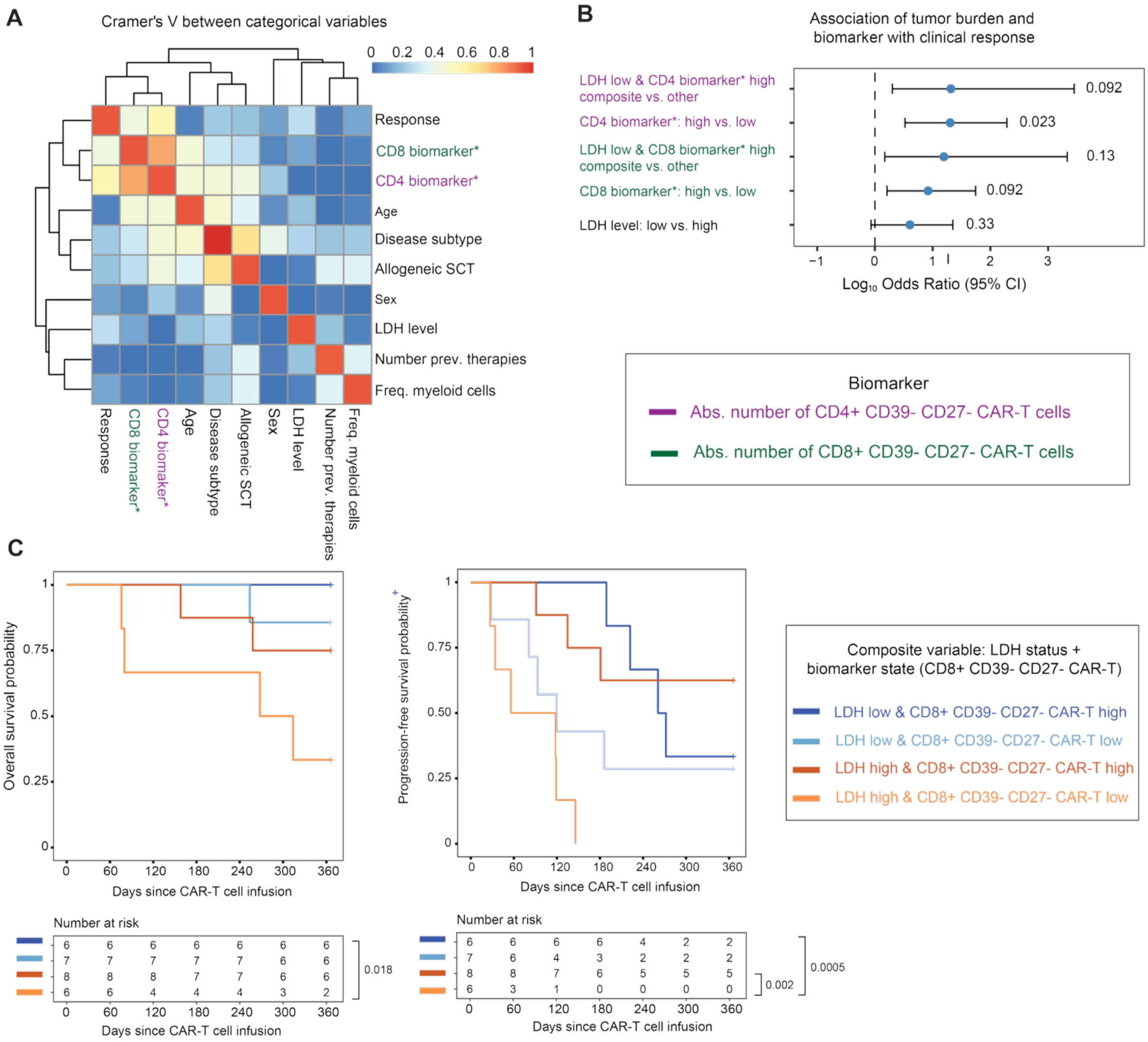

Supplementary Figure 4. Association between biomarkers, potential confounders and long-term outcome. A. Pairwise Cramér's V association matrix of categorical covariates, including clinical response. Values range from 0 (no association) to 1 (strong association). **B.** Forest plot with odds ratios for the association between clinical response, biomarkers and tumor burden. P values were obtained from Fisher's exact tests and adjusted for multiple comparisons using the Benjamini-Hochberg correction. The 95% confidence intervals are displayed. **C.** Kaplan-Meier curves for all patients stratified by a composite variable combining LDH level and the absolute number of $CD8^{+}CD27^{-}CD39^{-}$ CAR-T cells. Pairwise group differences were assessed with log-rank tests. Only significant comparisons are indicated.

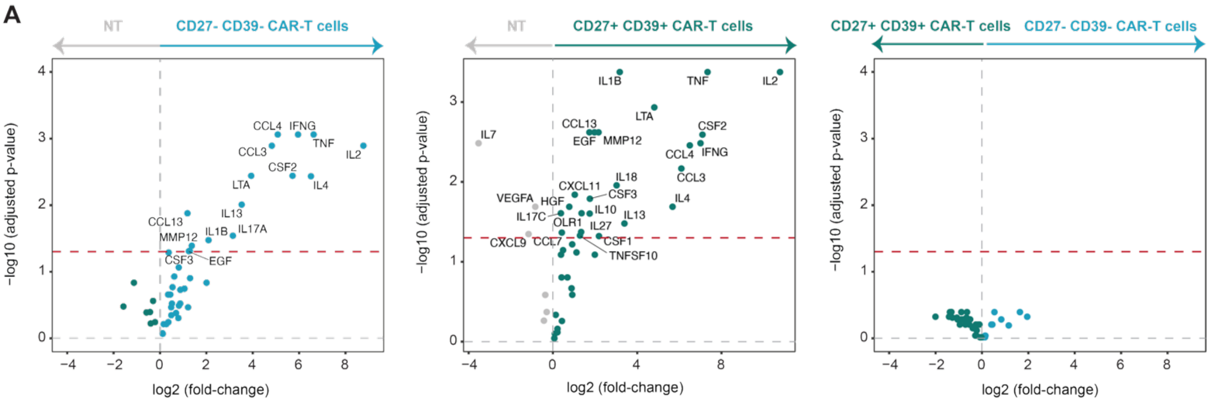

Supplementary Figure 5. Multiplex cytokine profiling of sorted CAR-T cell subsets. A. Olink-based cytokine profiling of supernatants from CAR-T–tumor cell co-cultures at a ratio of 1:1 after the first co-culture round. Left: non-transduced vs CD27^{-}CD39^{-} CAR-T cells. Middle: non-transduced vs CD27^{+}CD39^{+} CAR-T cells. Right: CD27^{+}CD39^{+} vs CD27^{-}CD39^{-} CAR-T cells. P values were determined with paired, two-sided t-tests and adjusted using the Benjamini-Hochberg method. Red dashed lines indicate the significance threshold.

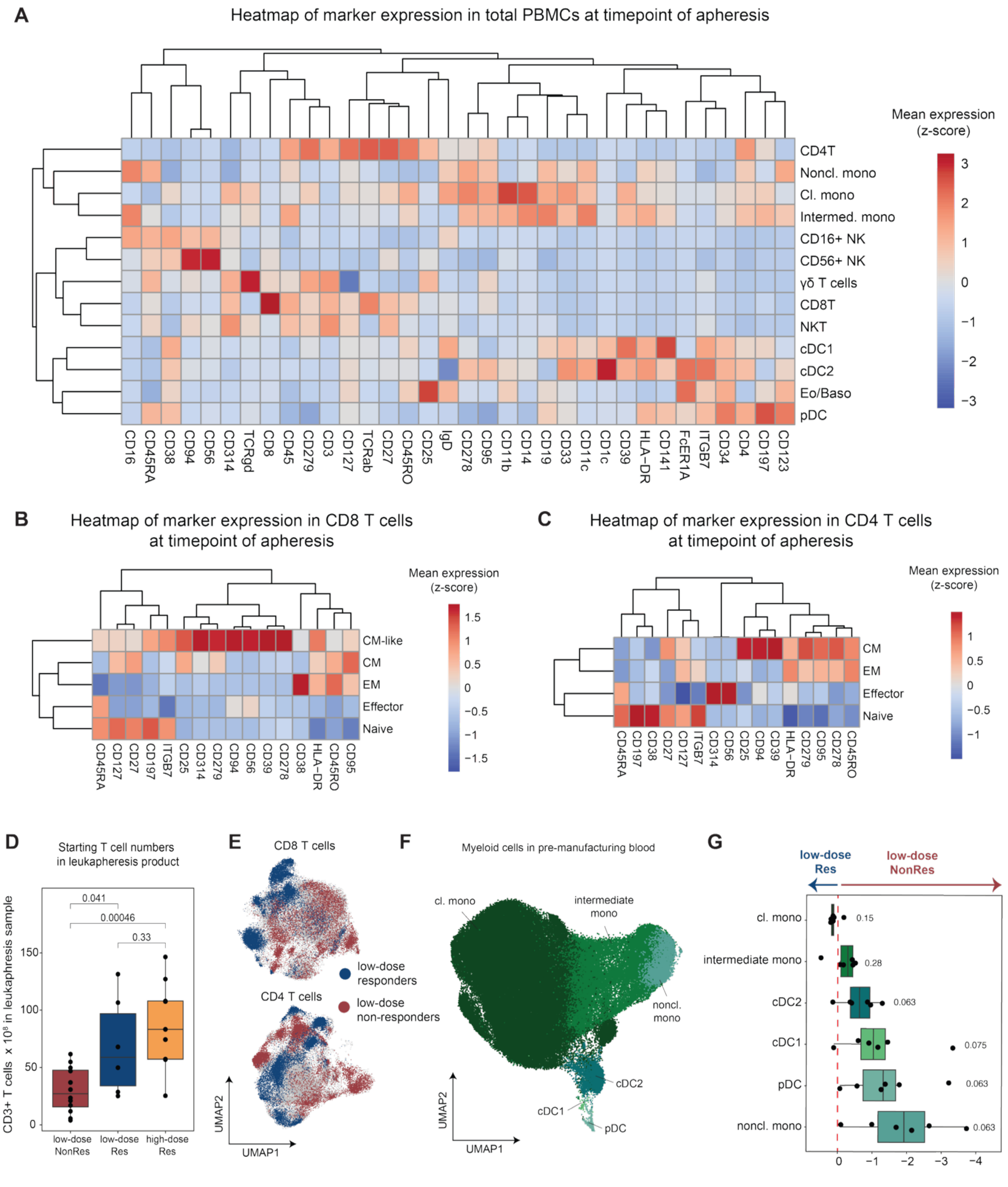

Supplementary Figure 6. Pre-manufacturing blood composition is associated with CAR-T cell therapy response. A. Heatmap with full marker expression (mean z-scored) in all PBMCs at timepoint of leukapheresis. **B.** Heatmap with mean T cell marker expression (z-scored) in CD8 T cell subsets. **C.** Heatmap with mean T cell marker expression (z-scored) in CD4 T cell subsets. **D.** Absolute number of CD3+ T cells in leukapheresis samples. P values were determined with a two-sided Wilcoxon rank-sum test. n = 28 patients. **E.** Differential abundance within CD8 (top) and CD4 (bottom) T cells using DA-seq comparing low-dose responders to low-dose non-responders. **F.** UMAP of the myeloid cells of the pre-manufacturing blood (n = 28). Out of 3,977,171 high-quality cells, 119,315 sketched cells are displayed. **G.** Quantitative comparison of relative frequencies within myeloid cells of low-dose responders compared to non-responders. A one-sample t-test (two-sided) was applied, and p

values were adjusted using Benjamini-Hochberg correction (n = 6). Abbreviations: UMAP = uniform manifold approximation and projection, Res = Responder, NonRes = Non-responder, cDC = conventional dendritic cells, pDC = plasmacytoid dendritic cells, cl.mono = classical monocytes, noncl. mono = non-classical monocytes. Box plots display the median, first and third quartiles and whiskers are defined as 1.5 times interquartile range.

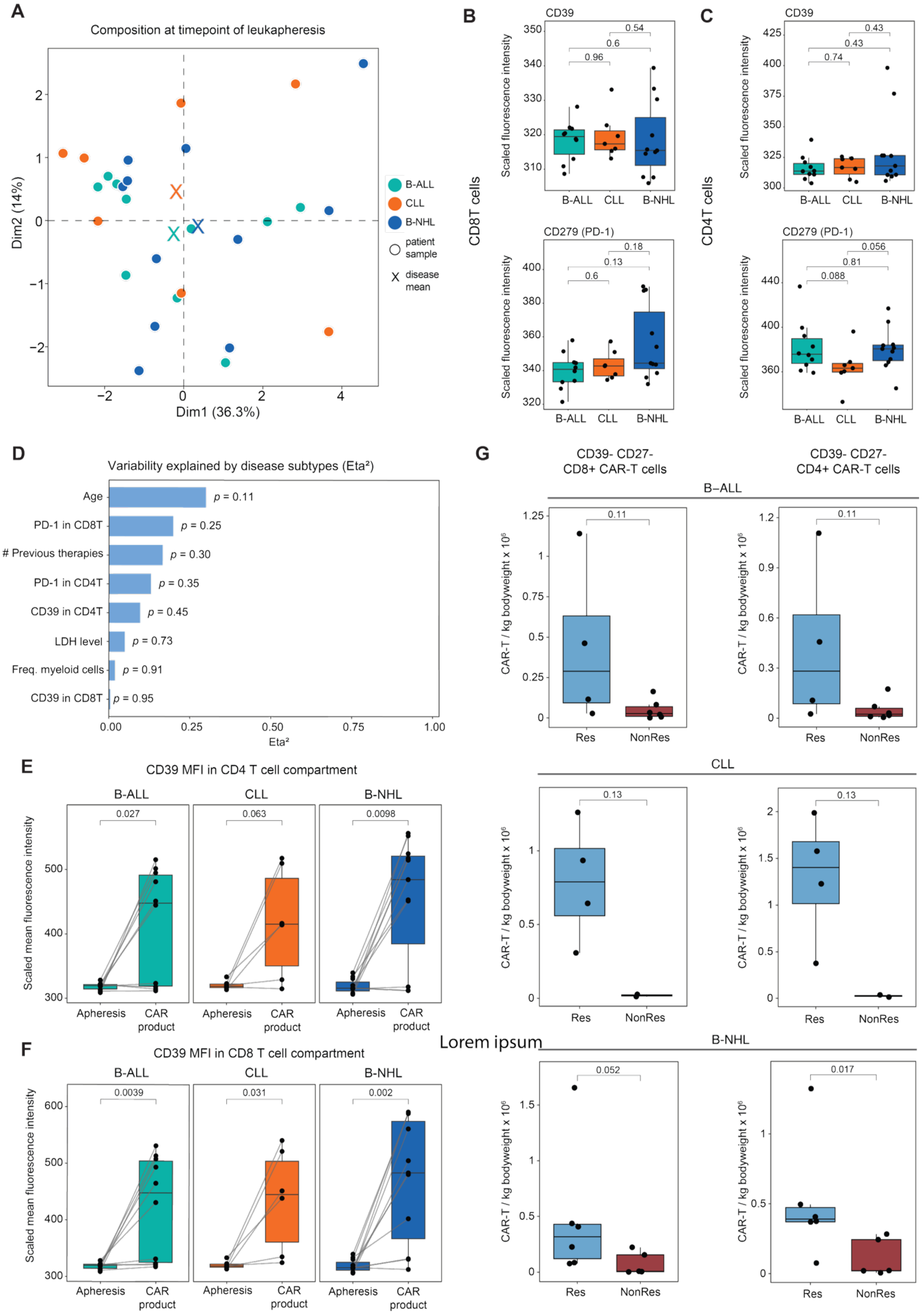

Supplementary Figure 7. Cohort heterogeneity and individual disease subtype analyses. A. Composition analysis using principal component analysis (PCA) of PBMCs at timepoint of leukapheresis, stratified by disease subtype. **B.** Scaled fluorescence intensity of

CD39 and CD279 (PD-1) in CD8 T cells at apheresis, stratified by disease subtype. P values were determined with a two-sided Wilcoxon rank-sum test. n = 27. **C.** Scaled fluorescence intensity of CD39 and CD279 (PD-1) in CD4 T cells at apheresis, stratified by disease subtype. P values were determined with a two-sided Wilcoxon rank-sum test. n = 27. **D.** Global η^2 (eta-squared) analysis of numeric variables by disease subtype using a one-way ANOVA test. P values are adjusted using Benjamini-Hochberg. **E.** Paired CD39 fluorescence intensity in CD8 T cells at apheresis versus CAR-T cell product across disease subtypes. P values were determined with a paired two-sided Wilcoxon rank-sum test. n = 27. **F.** Paired CD39 fluorescence intensity in CD4 T cells at apheresis versus CAR-T cell product across disease subtypes. P values were determined with a paired two-sided Wilcoxon rank-sum test. n = 27. **G.** Absolute numbers of $CD8^{+}CD27^{-}CD39^{-}$ and $CD4^{+}CD27^{-}CD39^{-}$ biomarker populations stratified by disease subtype. P values were determined with a two-sided Wilcoxon rank-sum test. B-ALL n = 10; CLL n = 6; B-NHL n = 11. Abbreviations: kg = kilogram, MFI = mean fluorescence intensity, B-ALL = B cell acute lymphoblastic leukemia, CLL = chronic lymphocytic leukemia, B-NHL = B cell non-Hodgkin's lymphoma. Box plots display the median, first and third quartiles and whiskers are defined as 1.5 times interquartile range.

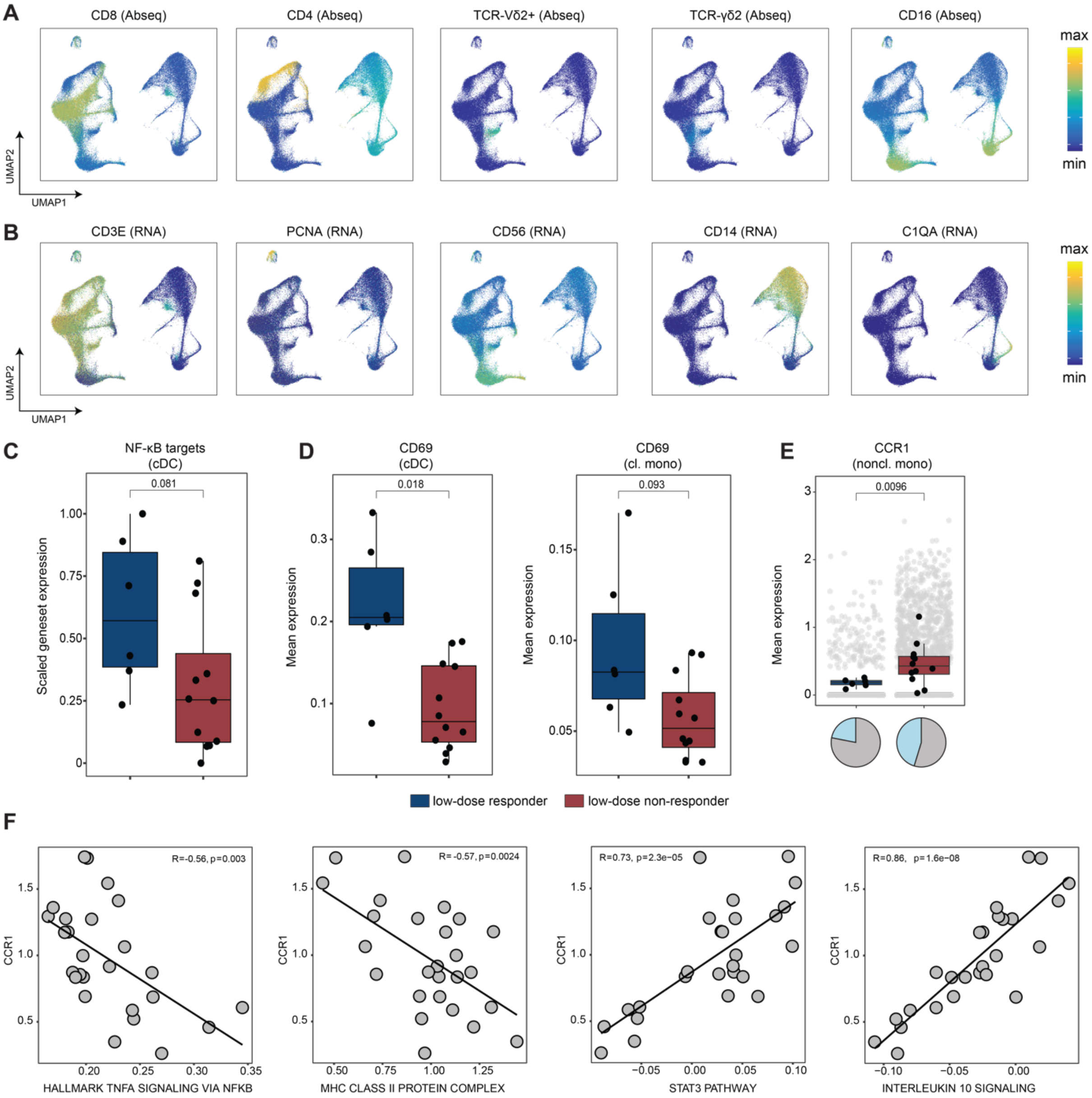

Supplementary Figure 8. Molecular programs of pre-manufacturing myeloid cells contribute to therapy response. A. Feature plots highlighting the Abseq expression levels of cell type specific surface markers within the single-cell proteo-genomics UMAP of pre-manufacturing blood (n=28 patients). **B.** Feature plots highlighting the mRNA expression levels of cell type specific genes within the single-cell proteo-genomics UMAP of pre-manufacturing blood (n = 28 patients). **C.** Gene set (NF-κB targets) in cDC and classical monocytes of low-dose recipients. Min-max scaled expression values are displayed. A two-sample t-test (two-sided) was applied. n = 18 patients**.** **D.** Boxplot of mean CD69 gene expression in cDC and classical monocytes of low-dose responders (n = 6) vs. low-dose non-responders (n = 12). **E.** Boxplot of mean CCR1 gene expression per patient in non-classical monocytes. Individual cells are plotted in the background. Pie charts represent the fraction of cells with expression (expression value > 0; blue) or without expression (expression value = 0; grey). n = 18 patients**. F.** Correlation of mean CCR1 gene expression and scaled expression of different gene sets. n = 27 patients. The Pearson correlation coefficient is displayed. Abbreviations: UMAP = uniform manifold approximation and projection, cDC = conventional dendritic cells, cl.mono = classical monocytes. Box plots display the median, first and third quartiles and whiskers are defined as 1.5 times interquartile range.

A

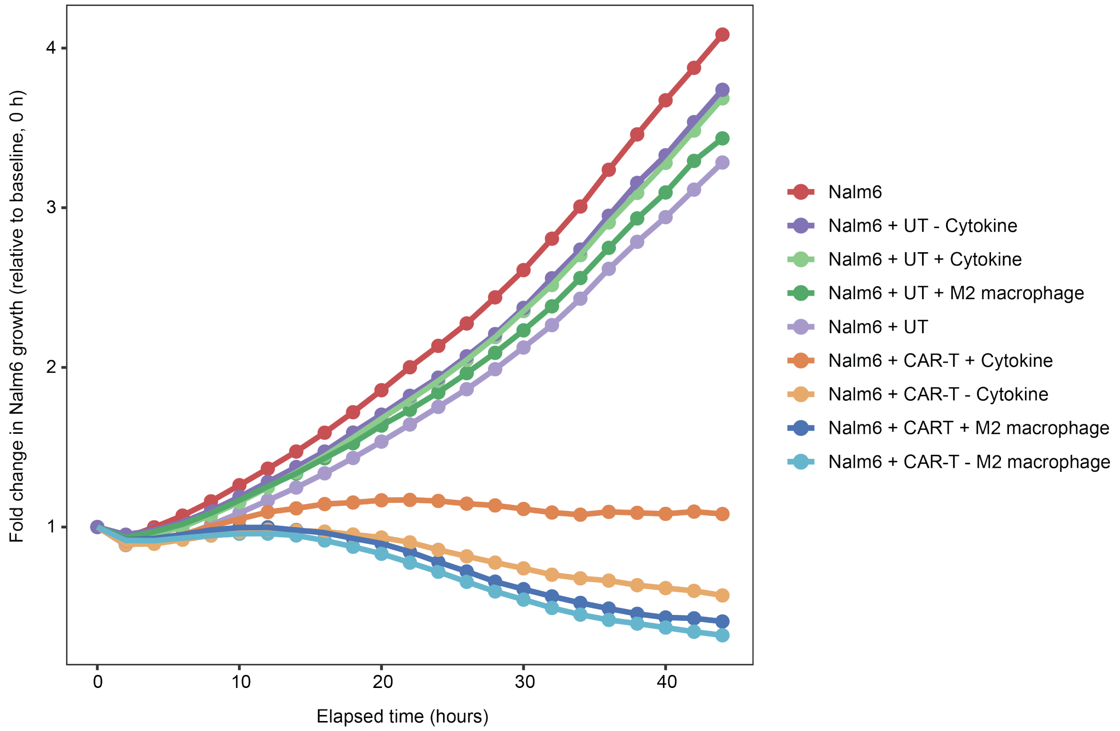

Supplementary Figure 9. Transient exposure to M2-macrophages alone does not impair leukemia cell killing. A. Nalm6 leukemia cell growth assessed with live-cell imaging across different conditions. All conditions used an effector-to-target ratio of 1:1. UT = untransduced T cells.

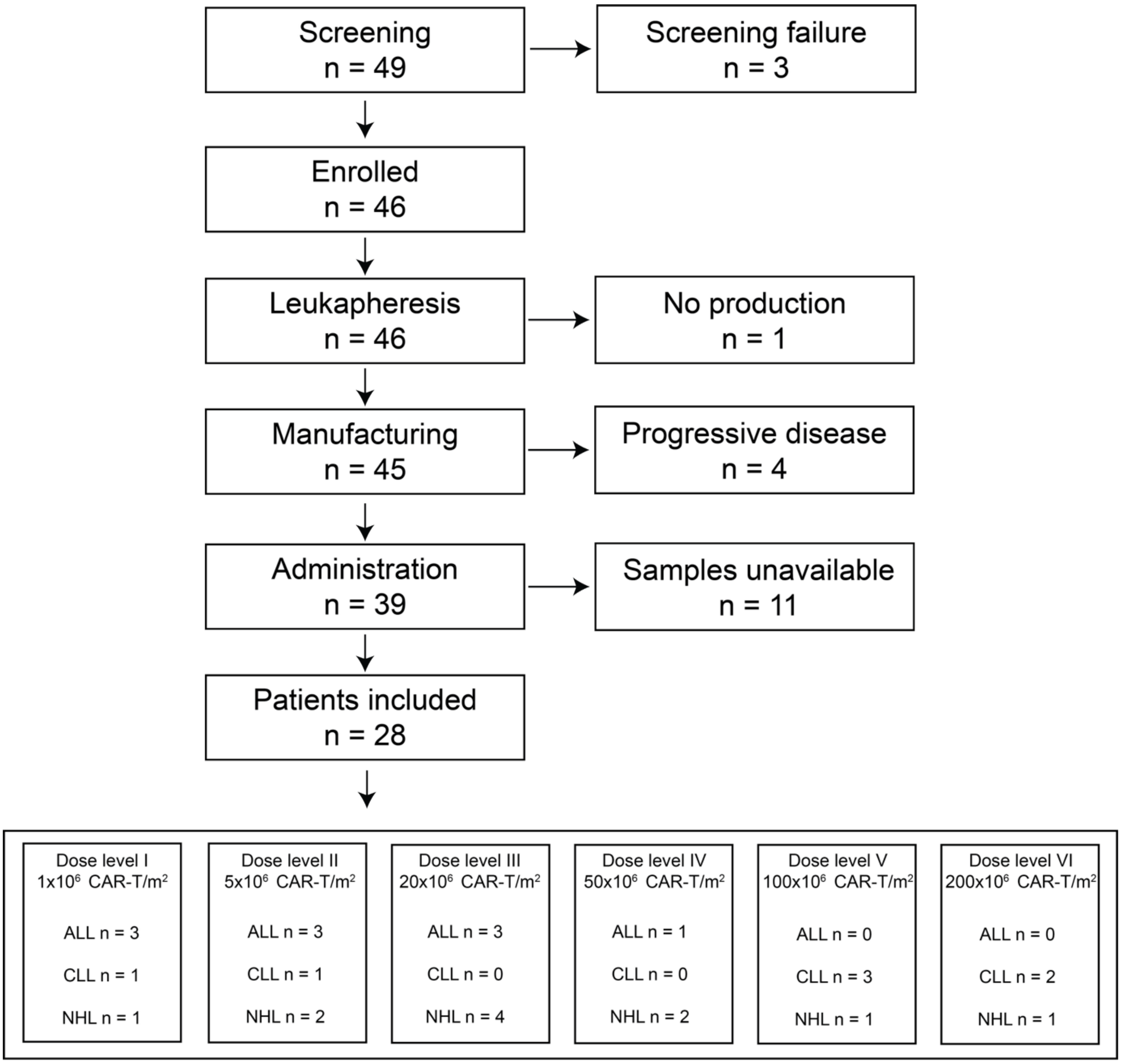

Supplementary Figure 10. Study profile of the HD-CAR-1 cohort. A total of 49 patients with relapsed and/or refractory B-cell malignancies were screened, of whom 46 were enrolled and underwent leukapheresis. CAR-T cell products were successfully manufactured for 45 patients and administered to 39 patients. For 28 of these patients, paired CAR-T cell product and PBMC samples obtained at the time of apheresis were available and included in the present study.

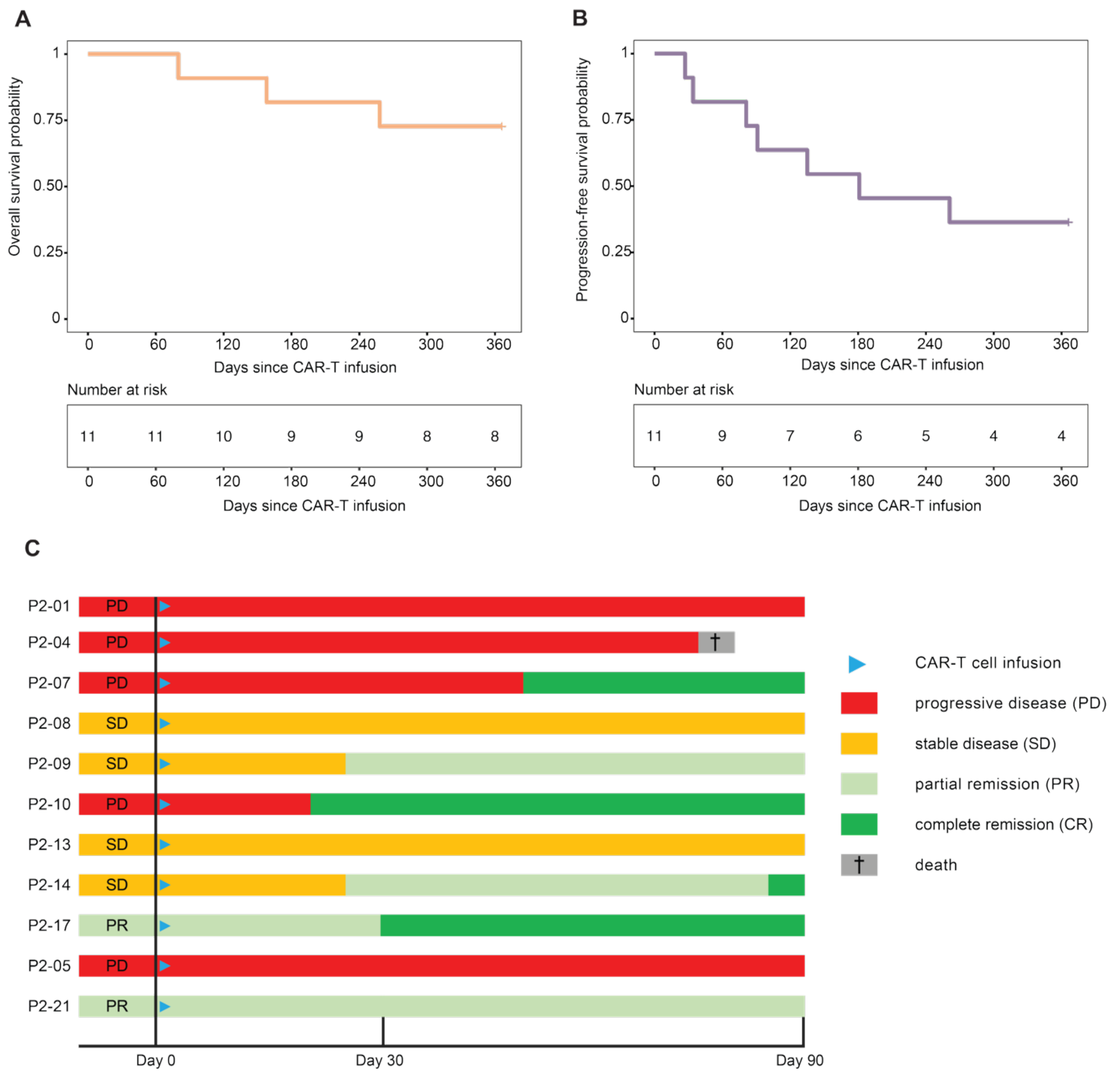

Supplementary Figure 11. Efficacy and clinical outcomes of HD-CAR-1 treatment in B-NHL patients. A. Overall survival (OS) and **B.** progression-free survival (PFS) of B-NHL patients. **C.** Swimmer plot depicting the course of individual B-NHL patients. Dose levels: DL1: P2-01; DL2: P2-05, P2-04; DL3: P2-07, P2-08, P2-09, P2-10; DL4: P2-13, P2-14; DL5: P2-17; DL6: P2-21. Abbreviations: PD = progressive disease, SD = stable disease, PR = partial remission, CR = complete remission.

Supplementary Table 1: Associations between clinical/ biological covariates and clinical response

Metadata	Odds ratio	p value	adj. p value
LDH low & CD4 biomarker* high: composite vs. other	1.31	0.0159	0.0923
CD4 biomarker*: high vs. low	1.3	0.00184	0.239
LDH low & CD8 biomarker* high: composite vs. other	1.19	0.0407	0.132
CD8 biomarker*: high vs. low	0.92	0.0213	0.0923
LDH level: low vs. high	0.6	0.128	0.334
Disease subtype: CLL vs. other Sex: M vs. F	0.34	0.648	0.762
Disease subtype: B-NHL vs. other Age : <58.	0.33	0.44	0.649
vs. > 58	0.079	1	1
# previous therapies: > 5 vs. < 5 Myeloid	-0.18	0.704	0.762
cells [%]: > 25 vs. < 25 Disease subtype: B-	-0.19	0.695	0.762
ALL vs. other Allogeneic stem cell	-0.32	0.449	0.649
transplantation: yes vs. no	-0.33	0.44	0.649
P values were assessed using Fisher's exact test and adjusted using Benjamini-Hochberg correction	-0.46	0.257	0.556

Supplementary Table 2: Panel used for spectral flow cytometry of CAR-T cell infusion products Figure 1

Antibodies and fluorochromes	Identifier (RRID)	Clone	Dilution 1:X	Staining round
Anti-CD16 BUV395	BD Biosciences Cat# 563785	3G8	100	3
Anti-CD19 BUV496	BD Biosciences Cat# 612938	SJ25C1	100	1
Anti-CD33 BUV563	BD Biosciences Cat# 741369	WM53	100	3
Anti-CD314 BUV615	BD Biosciences Cat# 751232	1D11	50	3
Anti-CD27 BUV661	BD Biosciences Cat# 741609	M-T271	100	3
Anti-CD8 BUV737	BD Biosciences Cat# 612754	SK1	100	3
Anti-CD45 BUV805	BD Biosciences Cat# 612891	HI30	200	3
Anti-CD141 BV421	BD Biosciences Cat# 565321	1A4	50	3
Anti-IgD Pacific Blue	Biolegend Cat# 348223	IA6-2	100	3
Anti-CD39 BV480	BD Biosciences Cat# 746454	TU66	100	3
Anti-CD278 BV510	BD Biosciences Cat# 744930	DX29	100	3
Anti-CD45RO BV570	Biolegend Cat# 304225	UCHL1	100	3
Anti-CD11c BV605	Biolegend Cat# 301636	3.9	50	3
Anti-CD279 BV650	BD Biosciences Cat# 564104	EH12.1	50	3
Anti-CD56 BV711	Biolegend Cat# 318336	HCD56	100	3
Anti-TCRab BV750	BD Biosciences Cat# 747180	IP26	75	3
Anti-CD45RA BV786	BD Biosciences Cat# 563870	HI100	200	3
Anti-CD11b BB515	BD Biosciences Cat# 564517	ICRF44	100	3
Anti-CD3 Spark Blue	Biolegend Cat# 344852	SK7	75	3
Anti-CD38 PerCP	Biolegend Cat# 303520	HIT2	50	3
Anti-CD94 BB700	BD Biosciences Cat# 566534	HP-3D9	100	3
Anti-TCRgd PerCP-eFluor710	Invitrogen Cat# 46-9959-42	B1.1	50	3
CAR-T detection reagent PE	Miltenyi Cat# 130-129-550		15	2
Anti-CD1c PE Dazzle594	Biolegend Cat# 331532	L161	50	3
Anti-CD95 PE-Fire640	Biolegend Cat# 305657	DX2	100	3
Anti-ITGB7 Pe-Cy5	BD Biosciences Cat#	FIB504	10	3
Anti-CD25 PE-Fire700	Biolegend Cat# 356145	M-A251	50	3
Anti-FcER1A PE-Cy7	Biolegend Cat# 334620	AER-37	100	3
Anti-CD4 RB780	BD Biosciences Cat# 568605	SK3	100	3
Anti-CD197 APC	BD Biosciences Cat# 566762	2-L1-A	30	3
Anti-CD123 AF647	Biolegend Cat# 306024	6H6	50	3
Anti-CD14 SPARK-NIR	Biolegend Cat# 399209	S18004B	100	3
Anti-CD127 APC R700	BD Biosciences Cat# 565185	HIL-7R-M2	50	3
Live Dead Zombie NIR	Biolegend Cat# 423105		2000	3
Anti-CD34 APC-Cy7	Biolegend Cat# 343514	581	50	3
Anti-HLA-DR APC-Fire810	Biolegend Cat# 307674	L243	50	3

Supplementary Table 3: Panel used for spectral flow cytometry of pre-manufacturing blood Figure 3

**Antibodies and fluorochromes**	**Identifier (RRID)**	**Clone**	**Dilution 1:X**
Anti-CD16 BUV395	BD Biosciences Cat# 563785	3G8	100
Anti-CD19 BUV496	BD Biosciences Cat# 612938	SJ25C1	100
Anti-CD33 BUV563	BD Biosciences Cat# 741369	WM53	100
Anti-CD314 BUV615	BD Biosciences Cat# 751232	1D11	50
Anti-CD27 BUV661	BD Biosciences Cat# 741609	M-T271	100
Anti-CD8 BUV737	BD Biosciences Cat# 612754	SK1	100
Anti-CD45 BUV805	BD Biosciences Cat# 612891	HI30	200
Anti-CD141 BV421	BD Biosciences Cat# 565321	1A4	50
Anti-IgD Pacific Blue	Biolegend Cat# 348223	IA6-2	100
Anti-CD39 BV480	BD Biosciences Cat# 746454	TU66	100
Anti-CD278 BV510	BD Biosciences Cat# 744930	DX29	100
Anti-CD45RO BV570	Biolegend Cat# 304225	UCHL1	100
Anti-CD11c BV605	Biolegend Cat# 301636	3.9	50
Anti-CD279 BV650	BD Biosciences Cat# 564104	EH12.1	50
Anti-CD56 BV711	Biolegend Cat# 318336	HCD56	100
Anti-TCRab BV750	BD Biosciences Cat# 747180	IP26	75
Anti-CD45RA BV786	BD Biosciences Cat# 563870	HI100	200
Anti-CD11b BB515	BD Biosciences Cat# 564517	ICRF44	100
Anti-CD3 Spark Blue	Biolegend Cat# 344852	SK7	75
Anti-CD38 PerCP	Biolegend Cat# 303520	HIT2	50
Anti-CD94 BB700	BD Biosciences Cat# 566534	HP-3D9	100
Anti-TCRgd PerCP-eFluor710 Anti-	Invitrogen Cat# 46-9959-42	B1.1	50
CD1c PE Dazzle594	Biolegend Cat# 331532	L161	50
Anti-CD95 PE-Fire640	Biolegend Cat# 305657	DX2	100
Anti-ITGB7 Pe-Cy5	BD Biosciences Cat#	FIB504	10
Anti-CD25 PE-Fire700	Biolegend Cat# 356145	M-A251	50
Anti-FcER1A PE-Cy7	Biolegend Cat# 334620	AER-37	100
Anti-CD4 RB780	BD Biosciences Cat# 568605	SK3	100
Anti-CD197 APC	BD Biosciences Cat# 566762	2-L1-A	30
Anti-CD123 AF647	Biolegend Cat# 306024	6H6	50
Anti-CD14 SPARK-NIR	Biolegend Cat# 399209	S18004B	100
Anti-CD127 APC R700	BD Biosciences Cat# 565185	HIL-7R-M[illegible]	50
Live Dead Zombie NIR	Biolegend Cat# 423105		2000
Anti-CD34 APC-Cy7	Biolegend Cat# 343514	581	50
Anti-HLA-DR APC-Fire810	Biolegend Cat# 307674	L243	50

Supplementary Table 4: Abseq antibodies for single-cell proteo-genomics of pre-manufacturing blood Figure 4

Antibodies	Identifier (RRID)	Clone
Anti-CD101	BD Biosciences Cat# 940269	V7.1
Anti-CD116	BD Biosciences Cat# 940311	hGMCSFR-M1
Anti-CD119	BD Biosciences Cat# 940253	GIR-208
Anti-CD122	BD Biosciences Cat# 940504	Mik-β2
Anti-CD162	BD Biosciences Cat# 940227	KPL-1
Anti-CD268	BD Biosciences Cat# 940284	11C1
Anti-CD282	BD Biosciences Cat# 940366	11G7
Anti-CD303	BD Biosciences Cat# 940282	V24-785
Anti-CD329	BD Biosciences Cat# 940312	E10-286
Anti-CD337	BD Biosciences Cat# 940291	p30-15
Anti-CD36	BD Biosciences Cat# 940224	CLB-IVC7
Anti-CD41	BD Biosciences Cat# 940219	HIP8
Anti-CD43	BD Biosciences Cat# 940278	1G10
Anti-CD45RO	BD Biosciences Cat# 940022	UCHL1
Anti-CD49f	BD Biosciences Cat# 940160	GoH3
Anti-CD63	BD Biosciences Cat# 940243	H5C6
Anti-CD86	BD Biosciences Cat# 940315	BU63
Anti-CD89	BD Biosciences Cat# 940277	A59
Anti-CD93	BD Biosciences Cat# 940215	R139
Anti-CD96	BD Biosciences Cat# 940272	6F9
Anti-CX3CR1	BD Biosciences Cat# 940216	2A9-1
AntiCD182	BD Biosciences Cat# 940240	6C6
Anti-CD186	BD Biosciences Cat# 940234	13B 1E5
Anti-FcER1A	BD Biosciences Cat# 940220	AER-37
Anti-HLA-DR	BD Biosciences Cat# 940010	G46-6
Anti-TCRgd	BD Biosciences Cat# 940365	11F2
Anti-TCR vdelta2	BD Biosciences Cat# 940297	B6
Anti-CD103	BD Biosciences Cat# 940067	Ber-ACT8
Anti-CD117	BD Biosciences Cat# 940250	104D2
Anti-CD11a	BD Biosciences Cat# 940077	HI111
Anti-CD11b	BD Biosciences Cat# 940266	ICRF44
Anti-CD11c	BD Biosciences Cat# 940363	3.9
Anti-CD123	BD Biosciences Cat# 940020	7G3
Anti-CD127	BD Biosciences Cat# 940012	HIL-7R-M21
Anti-CD13	BD Biosciences Cat# 940044	WM15
Anti-CD133	BD Biosciences Cat# 940373	293C3
Anti-CD14	BD Biosciences Cat# 940257	M5E2
Anti-CD141	BD Biosciences Cat# 940079	1A4

Supplementary Table 5: Panel used for flow cytometry in the validation cohort
Figure 7

**Antibodies and fluorochromes**	**Identifier (RRID)**	**Clone**	**Dilution 1:X**
Anti-CD3 BUV563	BD Biosciences Cat# 741448	SK7	300
Anti-CD27 BUV661	BD Biosciences Cat# 750167	L128	100
Anti-CD39 PE-Fire810	Biolegend Cat# 328245	A1	200
CAR-T detection reagent PE	Miltenyi Cat# 130-129-550		15
Live Dead Zombie NIR	Biolegend Cat# 423105		2000
Anti-CD16 BUV395	BD Biosciences Cat# 563785	3G8	100
Anti-CD14 SPARK-NIR	Biolegend Cat# 399210	S18004E	100
Anti-CD123 AF647	Biolegend Cat# 306024	6H6	50
Anti-CD1c PE-Dazzle594	Biolegend Cat# 331532	L161	50
Anti-CD55 BV650	BD Biosciences Cat# 742680	IA10	75
Anti-CD69 PE-Cy5	BD Biosciences Cat# 555532	FN50	100

Supplementary Table 6: Panel used for flow cytometry of generated CAR-T cell products
Figure 5

**Antibodies and fluorochromes**	**Identifier (RRID)**	**Clone**	**Dilution 1:X**
Anti-CD3 APC	Biolegend Cat# 344812	SK7	100
Anti-CD4 APC-Cy7	Biolegend Cat# 317418	OKT4	100
Anti-CD8 Pacific Blue	Biolegend Cat# 344718	SK1	100
Anti-CD33 FITC	Biolegend Cat# 366620	P67.6	100
Anti-CD39 BV480	BD Biosciences Cat# 746454	TU66	100
Anti-CD16 PE	BD Biosciences Cat# 561313	B73.1	100
7-AAD (Live/Dead)	BD Biosciences Cat# 559925		20
Anti-CD4 FITC	Biolegend Cat# 317408	OKT4	100
Anti-CD27 PE-Cy7	Biolegend Cat# 302802	323	100
Streptavidin PE	BD Biosciences Cat# 554061		100
Purified Recomb Biotinylated Pr	Therfo Fisher Scientific Cat# 29997		10
