# Supplementary material for: A distinct CAR-T cell phenotype mediates therapeutic response at limited doses
Source: Nat Commun. 2026 Jul 29;17:7589. doi: 10.1038/s41467-026-76068-4 (PMC13421456; doi:10.1038/s41467-026-76068-4)
Supplement: Supplementary file 2 — Description of Additional Supplementary Files [file 41467_2026_76068_MOESM2_ESM.pdf]

Legends of Supplementary Data

File name: Supplementary_Data_1_Yousefian_etal_2026.xlsx

Description: This file contains the inclusion criteria, patient characteristics, and clinical parameters of the HD-CAR-1 trial

File name: Supplementary_Data_2_Yousefian_etal_2026.xlsx

Description: This file contains the characteristics of patients included in the independent validation cohort.
