# Supplementary material for: A distinct CAR-T cell phenotype mediates therapeutic response at limited doses
Source: Nat Commun. 2026 Jul 29;17:7589. doi: 10.1038/s41467-026-76068-4 (PMC13421456; doi:10.1038/s41467-026-76068-4)
Supplement: Supplementary file 5 — Reporting Summary [file 41467_2026_76068_MOESM5_ESM.pdf]

Corresponding author(s): Haas, Simon

Last updated by author(s): 20.05.2026

Reporting Summary

Nature Portfolio wishes to improve the reproducibility of the work that we publish. This form provides structure for consistency and transparency in reporting. For further information on Nature Portfolio policies, see our Editorial Policies and the Editorial Policy Checklist.

Statistics

For all statistical analyses, confirm that the following items are present in the figure legend, table legend, main text, or Methods section.

n/a	Confirmed	
☐	☒	The exact sample size (*n*) for each experimental group/condition, given as a discrete number and unit of measurement
☐	☒	A statement on whether measurements were taken from distinct samples or whether the same sample was measured repeatedly
☐	☒	The statistical test(s) used AND whether they are one- or two-sided<br>*Only common tests should be described solely by name; describe more complex techniques in the Methods section.*
☐	☒	A description of all covariates tested
☐	☒	A description of any assumptions or corrections, such as tests of normality and adjustment for multiple comparisons
☐	☒	A full description of the statistical parameters including central tendency (e.g. means) or other basic estimates (e.g. regression coefficient) AND variation (e.g. standard deviation) or associated estimates of uncertainty (e.g. confidence intervals)
☐	☒	For null hypothesis testing, the test statistic (e.g. *F*, *t*, *r*) with confidence intervals, effect sizes, degrees of freedom and *P* value noted<br>*Give P values as exact values whenever suitable.*
☒	☐	For Bayesian analysis, information on the choice of priors and Markov chain Monte Carlo settings
☒	☐	For hierarchical and complex designs, identification of the appropriate level for tests and full reporting of outcomes
☒	☐	Estimates of effect sizes (e.g. Cohen's *d*, Pearson's *r*), indicating how they were calculated

Our web collection on statistics for biologists contains articles on many of the points above.

Software and code

Policy information about availability of computer code

Data collection	For the acquisition of flow cytometry data, the SpectroFlo® (Cytek Biosciences, v.3.2.1) software used.<br>Raw sequencing data were pre-processed using the BD Rhapsody analysis pipeline (v2.0; BD Biosciences) according to the manufacturer's recommendations and instructions.
Data analysis	Flow cytometry data was pre-processed using FlowJo v10.8.1 and flowAI R package (v1.24.0).<br><br>All downstream analyses were performed in R v4.3.0. The following packages were used: Seurat v5.0.3, BPCells v0.1.0, cowplot v.1.1.1, ggpubr v0.6.0, viridis v0.6.3, purrr v1.0.1, tidyr v.1.3.0, tibble v.3.2.1, tidyverse v2.0.0, Matrix v1.6-5, patchwork v1.1.2, ggrepel v0.9.5, ggplot2 v3.4.4, SeuratObject v5.0.1.9008, dplyr v.1.1.2, data.table v1.14.8, MASS v7.3-60, sctransform v0.4.1, RColorBrewer v.1.1-3, stats4 v4.3.0, WGCNA v1.73, scWGCNA v1.0.0, pals v1.7, miloDE v0.0.0.9000, GenomicRanges v1.52.0, GenomeInfoDb v1.36.0, limma v3.56.2, edgeR v3.42.4, randomForest v4.7-1.1, scales v1.3.0, DAseq v1.0.0, miloR v1.8.1, msigdbr v7.5.1, fgsea v1.26.0, DElegate v1.2.1<br><br>A detailed description of flow cytometry and single-cell proteo-genomics data analysis is provided in the Methods.<br><br>Code for key results is provided in the Zenodo repository: https://doi.org/10.5281/zenodo.14711865<br><br>Raw and processed sequencing data have been submitted to NCBI Gene Expression Omnibus (GEO) and are currently being processed by GEO.

For manuscripts utilizing custom algorithms or software that are central to the research but not yet described in published literature, software must be made available to editors and reviewers. We strongly encourage code deposition in a community repository (e.g. GitHub). See the Nature Portfolio guidelines for submitting code & software for further information.

Data

Policy information about availability of data

All manuscripts must include a data availability statement. This statement should provide the following information, where applicable:

- Accession codes, unique identifiers, or web links for publicly available datasets
- A description of any restrictions on data availability
- For clinical datasets or third party data, please ensure that the statement adheres to our policy

High quality flow cytometry data, single-cell proteo-genomics data and clinical metadata are provided under the following link: https://doi.org/10.5281/zenodo.14711865.
The sequencing data generated in this study have been deposited in the NCBI Gene Expression Omnibus (GEO) under accession number GSE314571. Source data are provided with this paper and can also be found in the Zenodo repository.

Research involving human participants, their data, or biological material

Policy information about studies with human participants or human data. See also policy information about sex, gender (identity/presentation), and sexual orientation and race, ethnicity and racism.

Reporting on sex and gender	Female and male patients with confirmed CD19-positive and previously treated hematologic malignancy were eligible for participation in the HD-CAR-1 trial and subsequent analysis. Trial population consisted of both genders, determined based on self-report. Within the written consent of HD-CAR-1, consent for reporting demographic and clinical data including self-reported gender was obtained. Therapy response was not significantly associated with sex or age. A summary of sex and gender can be found in Supplementary Table 1.<br><br>Characteristics, including the sex of patients included in the validation cohort are summarized in Supplementary Table 8.
Reporting on race, ethnicity, or other socially relevant groupings	No information on race, ethnicity, or other socially relevant groupings was collected or used for analysis within the HD-CAR-1 trial.
Population characteristics	Patients with confirmed CD19+ and previously treated hematologic malignancy were eligible for participation in the HD-CAR-1 trial and subsequent analysis.<br><br>For inclusion and exclusion criteria see NCT03676504 (clinivaltrials.gov) trial registration and:<br>- Schubert ML, Schmitt A, Sellner L, Neuber B, Kunz J, Wuchter P, Kunz A, Gern U, Michels B, Hofmann S, Hückelhoven-Krauss A, Kulozik A, Ho AD, Müller-Tidow C, Dreger P, Schmitt M. Treatment of patients with relapsed or refractory CD19+ lymphoid disease with T lymphocytes transduced by RV-SFG.CD19.CD28.4-1BBzeta retroviral vector: a unicentre phase I/II clinical trial protocol. BMJ Open. 2019 May 19;9(5):e026644. doi: 10.1136/bmjopen-2018-026644. PMID: 31110096.<br><br>Population characteristics of the ALL and CLL cohort have been previously published:<br>-Schubert ML, Schmitt A, Hückelhoven-Krauss A, Neuber B, Kunz A, Waldhoff P, Vonficht D, Yousefian S, Jopp-Saile L, Wang L, Korell F, Keib A, Michels B, Haas D, Sauer T, Derigs P, Kulozik A, Kunz J, Pavel P, Laier S, Wuchter P, Schmier J, Bug G, Lang F, Gökbuget N, Casper J, Görner M, Finke J, Neubauer A, Ringhoffer M, Wolleschak D, Brüggemann M, Haas S, Ho AD, Müller-Tidow C, Dreger P, Schmitt M. Treatment of adult ALL patients with third-generation CD19-directed CAR T cells: results of a pivotal trial. J Hematol Oncol. 2023 Jul 22;16(1):79. doi: 10.1186/s13045-023-01470-0. PMID: 37481608;<br><br>- Derigs P, Schubert ML, Dreger P, Schmitt A, Yousefian S, Haas S, Röthemeier C, Neuber B, Hückelhoven-Krauss A, Brüggemann M, Bernhard H, Kobbe G, Lindemann A, Rummel M, Michels B, Korell F, Ho AD, Müller-Tidow C, Schmitt M. Third-generation anti-CD19 CAR T cells for relapsed/refractory chronic lymphocytic leukemia: a phase 1/2 study. Leukemia. 2024 Nov;38(11):2419-2428. doi: 10.1038/s41375-024-02392-7. PMID: 39192036.<br><br>Population characteristics of the B-HL cohort are presented in Supplementary Table 1. Clinical efficacy and outcome data for the B-NHL cohort are presented in Supplementary Figure 11.<br><br>Population characteristics of the validation cohort are presented in Supplementary Table 8.
Recruitment	Patients with confirmed CD19+ and previously treated hematologic malignancy were screened in the present study; inclusion and exclusion criteria are summarized under trial registration NCT03676504 (clinicaltrials.gov) have been published and are available under:<br><br>- Schubert ML, Schmitt A, Sellner L, Neuber B, Kunz J, Wuchter P, Kunz A, Gern U, Michels B, Hofmann S, Hückelhoven-Krauss A, Kulozik A, Ho AD, Müller-Tidow C, Dreger P, Schmitt M. Treatment of patients with relapsed or refractory CD19+ lymphoid disease with T lymphocytes transduced by RV-SFG.CD19.CD28.4-1BBzeta retroviral vector: a unicentre phase I/II clinical trial protocol. BMJ Open. 2019 May 19;9(5):e026644. doi: 10.1136/bmjopen-2018-026644. PMID: 31110096.<br>Prior to screening, written informed consent was obtained from all participants after verbal and written information about the study protocol. Patients did not receive compensation for participating in the study.

Ethics oversight	Ethical approval and approvals from the local and federal competent authorities were granted. HD-CAR-1 trial protocol received Institutional Review Board approval from the EC of the University of Heidelberg in October 2017 (AFmu-405/2017).<br><br>Patient samples of the validation cohort were obtained after written informed consent in accordance with the Declaration of Helsinki. The study was approved by the institutional ethics committee of the Charité Universitätsmedizin Berlin (Ethics approval number EA2/142/20).

Note that full information on the approval of the study protocol must also be provided in the manuscript.

Field-specific reporting

Please select the one below that is the best fit for your research. If you are not sure, read the appropriate sections before making your selection.

- [x] Life sciences
- [ ] Behavioural & social sciences
- [ ] Ecological, evolutionary & environmental sciences

For a reference copy of the document with all sections, see nature.com/documents/nr-reporting-summary-flat.pdf

Life sciences study design

All studies must disclose on these points even when the disclosure is negative.

Sample size	No formal statistical methods were used to predetermine the sample size. The analyses were based on available samples from the HD-CAR-1 clinical trial. All eligible samples from these studies were included to maximize data use and ensure representativeness. The combined sample size (n=28) is sufficient to support the exploratory objectives of our study.<br><br>For validation purposes, patient samples from an independent cohort were used (n=42).
Data exclusions	The infusion product of P2-19 was unavailable on the day of measurement and was excluded from the flow cytometry analysis. For the apheresis sample of P2-18 not sufficient myeloid cells could be captured during single-cell proteo-genomics preparation. Therefore this sample was excluded from analyses specifically involving myeloid cells.<br><br>The information can be also found in Supplementary Table 1.
Replication	This study was based on a single set of 28 unique biological patient samples obtained from the HD-CAR-1 clinical trial. No technical or biological replicates were performed. Functional experiments were generally performed with at least n=6 biologically independent donors or independent experiments, and selected assays were repeated with additional samples to confirm the robustness of the observed effects. Biomarker findings from the discovery cohort were further assessed in an independent validation cohort comprising different disease entities, CAR targets and commercial CAR-T products. All replication and validation analyses supported the main conclusions of the study.<br><br>All experiments and analyses were applied consistently across all samples using standardized protocols to support internal consistency and reproducibility of the results.
Randomization	This study involved a retrospective analysis of patient samples from the HD-CAR-1 clinical trial. Patients had already been classified as responders or non-responders based on predefined clinical criteria as outlined in our methods section. Therefore, no additional allocation or randomization was performed.
Blinding	Blinding was not performed, as this was a retrospective study analyzing previously collected and categorized samples. Investigators had access to response and dose level classifications during the analysis.

Reporting for specific materials, systems and methods

We require information from authors about some types of materials, experimental systems and methods used in many studies. Here, indicate whether each material, system or method listed is relevant to your study. If you are not sure if a list item applies to your research, read the appropriate section before selecting a response.

Materials & experimental systems

n/a	Involved in the study	
[ ]	[x]	Antibodies
[ ]	[x]	Eukaryotic cell lines
[x]	[ ]	Palaeontology and archaeology
[x]	[ ]	Animals and other organisms
[ ]	[x]	Clinical data
[x]	[ ]	Dual use research of concern
[x]	[ ]	Plants

Methods

n/a	Involved in the study	
[x]	[ ]	ChIP-seq
[ ]	[x]	Flow cytometry
[x]	[ ]	MRI-based neuroimaging

nature portfolio | reporting summary

Antibodies

Antibodies used

Antibodies (Epitope, Fluorochrome, Vendor, Identifier (RRID), Clone):

Flow cytometry analysis CAR-T cell product:

Anti-CD16 BUV395 BD Biosciences Cat# 563785 3G8
Anti-CD19 BUV496 BD Biosciences Cat# 612938 SJ25C1
Anti-CD33 BUV563 BD Biosciences Cat# 741369 WM53
Anti-CD314 BUV615 BD Biosciences Cat# 751232 1D11
Anti-CD27 BUV661 BD Biosciences Cat# 741609 M-T271
Anti-CD8 BUV737 BD Biosciences Cat# 612754 SK1
Anti-CD45 BUV805 BD Biosciences Cat# 612891 HI30
Anti-CD141 BV421 BD Biosciences Cat# 565321 1A4
Anti-IgD Pacific Blue Biolegend Cat# 348223 IA6-2
Anti-CD39 BV480 BD Biosciences Cat# 746454 TU66
Anti-CD278 BV510 BD Biosciences Cat# 744930 DX29
Anti-CD45RO BV570 Biolegend Cat# 304225 UCHL1
Anti-CD11c BV605 Biolegend Cat# 301636 3.9
Anti-CD279 BV650 BD Biosciences Cat# 564104 EH12.1
Anti-CD56 BV711 Biolegend Cat# 318336 HCD56
Anti-TCRab BV750 BD Biosciences Cat# 747180 IP26
Anti-CD45RA BV786 BD Biosciences Cat# 563870 HI100
Anti-CD11b BB515 BD Biosciences Cat# 564517 ICRF44
Anti-CD3 Spark Blue Biolegend Cat# 344852 SK7
Anti-CD38 PerCP Biolegend Cat# 303520 HIT2
Anti-CD94 BB700 BD Biosciences Cat# 566534 HP-3D9
Anti-TCRgd PerCP-eFluor710 Invitrogen Cat# 46-9959-42 B1.1
CAR-T detection reagent PE Miltenyi Cat# 130-129-550
Anti-CD1c PE Dazzle594 Biolegend Cat# 331532 L161
Anti-CD95 PE-Fire640 Biolegend Cat# 305657 DX2
Anti-ITGB7 Pe-Cy5 BD Biosciences Cat# FIB504
Anti-CD25 PE-Fire700 Biolegend Cat# 356145 M-A251
Anti-FcER1A PE-Cy7 Biolegend Cat# 334620 AER-37
Anti-CD4 RB780 BD Biosciences Cat# 568605 SK3
Anti-CD197 APC BD Biosciences Cat# 566762 2-L1-A
Anti-CD123 AF647 Biolegend Cat# 306024 6H6
Anti-CD14 SPARK-NIR Biolegend Cat# 399209 S18004B
Anti-CD127 APC R700 BD Biosciences Cat# 565185 HIL-7R-M21
Live Dead Zombie NIR Biolegend Cat# 423105
Anti-CD34 APC-Cy7 Biolegend Cat# 343514 581
Anti-HLA-DR APC-Fire810 Biolegend Cat# 307674 L243

Flow cytometry analysis pre-manufacturing blood samples:

Anti-CD16 BUV395 BD Biosciences Cat# 563785 3G8
Anti-CD19 BUV496 BD Biosciences Cat# 612938 SJ25C1
Anti-CD33 BUV563 BD Biosciences Cat# 741369 WM53
Anti-CD314 BUV615 BD Biosciences Cat# 751232 1D11
Anti-CD27 BUV661 BD Biosciences Cat# 741609 M-T271
Anti-CD8 BUV737 BD Biosciences Cat# 612754 SK1
Anti-CD45 BUV805 BD Biosciences Cat# 612891 HI30
Anti-CD141 BV421 BD Biosciences Cat# 565321 1A4
Anti-IgD Pacific Blue Biolegend Cat# 348223 IA6-2
Anti-CD39 BV480 BD Biosciences Cat# 746454 TU66
Anti-CD278 BV510 BD Biosciences Cat# 744930 DX29
Anti-CD45RO BV570 Biolegend Cat# 304225 UCHL1
Anti-CD11c BV605 Biolegend Cat# 301636 3.9
Anti-CD279 BV650 BD Biosciences Cat# 564104 EH12.1
Anti-CD56 BV711 Biolegend Cat# 318336 HCD56
Anti-TCRab BV750 BD Biosciences Cat# 747180 IP26
Anti-CD45RA BV786 BD Biosciences Cat# 563870 HI100
Anti-CD11b BB515 BD Biosciences Cat# 564517 ICRF44
Anti-CD3 Spark Blue Biolegend Cat# 344852 SK7
Anti-CD38 PerCP Biolegend Cat# 303520 HIT2
Anti-CD94 BB700 BD Biosciences Cat# 566534 HP-3D9
Anti-TCRgd PerCP-eFluor710 Invitrogen Cat# 46-9959-42 B1.1

Anti-CD1c PE Dazzle594 Biolegend Cat# 331532 L161
Anti-CD95 PE-Fire640 Biolegend Cat# 305657 DX2
Anti-ITGB7 Pe-Cy5 BD Biosciences Cat# FIB504
Anti-CD25 PE-Fire700 Biolegend Cat# 356145 M-A251
Anti-FcER1A PE-Cy7 Biolegend Cat# 334620 AER-37
Anti-CD4 RB780 BD Biosciences Cat# 568605 SK3
Anti-CD197 APC BD Biosciences Cat# 566762 2-L1-A
Anti-CD123 AF647 Biolegend Cat# 306024 6H6
Anti-CD14 SPARK-NIR Biolegend Cat# 399209 S18004B
Anti-CD127 APC R700 BD Biosciences Cat# 565185 HIL-7R-M21
Live Dead Zombie NIR Biolegend Cat# 423105
Anti-CD34 APC-Cy7 Biolegend Cat# 343514 581
Anti-HLA-DR APC-Fire810 Biolegend Cat# 307674 L243

Abseq antibodies (no fluorochrome attached) for single-cell proteo-genomics:

Anti-CD101 BD Biosciences Cat# 940269 V7.1
Anti-CD116 BD Biosciences Cat# 940311 hGMCSFR-M1
Anti-CD119 BD Biosciences Cat# 940253 GIR-208
Anti-CD122 BD Biosciences Cat# 940504 Mik-β2
Anti-CD162 BD Biosciences Cat# 940227 KPL-1
Anti-CD268 BD Biosciences Cat# 940284 11C1
Anti-CD282 BD Biosciences Cat# 940366 11G7
Anti-CD303 BD Biosciences Cat# 940282 V24-785
Anti-CD329 BD Biosciences Cat# 940312 E10-286
Anti-CD337 BD Biosciences Cat# 940291 p30-15
Anti-CD36 BD Biosciences Cat# 940224 CLB-IVC7
Anti-CD41 BD Biosciences Cat# 940219 HIP8
Anti-CD43 BD Biosciences Cat# 940278 1G10
Anti-CD45RO BD Biosciences Cat# 940022 UCHL1
Anti-CD49f BD Biosciences Cat# 940160 GoH3
Anti-CD63 BD Biosciences Cat# 940243 H5C6
Anti-CD86 BD Biosciences Cat# 940315 BU63
Anti-CD89 BD Biosciences Cat# 940277 A59
Anti-CD93 BD Biosciences Cat# 940215 R139
Anti-CD96 BD Biosciences Cat# 940272 6F9
Anti-CX3CR1 BD Biosciences Cat# 940216 2A9-1
AntiCD182 BD Biosciences Cat# 940240 6C6
Anti-CD186 BD Biosciences Cat# 940234 13B 1E5
Anti-FcER1A BD Biosciences Cat# 940220 AER-37
Anti-HLA-DR BD Biosciences Cat# 940010 G46-6
Anti-TCRgd BD Biosciences Cat# 940365 11F2
Anti-TCR vdelta2 BD Biosciences Cat# 940297 B6
Anti-CD103 BD Biosciences Cat# 940067 Ber-ACT8
Anti-CD117 BD Biosciences Cat# 940250 104D2
Anti-CD11a BD Biosciences Cat# 940077 HI111
Anti-CD11b BD Biosciences Cat# 940266 ICRF44
Anti-CD11c BD Biosciences Cat# 940363 3.9
Anti-CD123 BD Biosciences Cat# 940020 7G3
Anti-CD127 BD Biosciences Cat# 940012 HIL-7R-M21
Anti-CD13 BD Biosciences Cat# 940044 WM15
Anti-CD133 BD Biosciences Cat# 940373 293C3
Anti-CD14 BD Biosciences Cat# 940257 M5E2
Anti-CD141 BD Biosciences Cat# 940079 1A4
Anti-CD16 BD Biosciences Cat# 940006 3G8
Anti-CD18 BD Biosciences Cat# 940086 6.7
Anti-CD183 BD Biosciences Cat# 940030 1C6/CXCR3
Anti-CD184 BD Biosciences Cat# 940056 12G5
Anti-CD185 BD Biosciences Cat# 940042 RF8B2
Anti-CD19 BD Biosciences Cat# 940004 SJ25C1
Anti-CD194 BD Biosciences Cat# 940047 1G1
Anti-CD196 BD Biosciences Cat# 940033 11A9
Anti-CD197 BD Biosciences Cat# 940014 3D12
Anti-CD2 BD Biosciences Cat# 940046 RPA-2.10
Anti-CD1c BD Biosciences Cat# 940083 F10/21A3
Anti-CD206 BD Biosciences Cat# 940068 19.2
Anti-CD223 BD Biosciences Cat# 940080 T47-530
Anti-CD226 BD Biosciences Cat# 940075 DX11
Anti-CD25 BD Biosciences Cat# 940009 2A3

Anti-CD26 BD Biosciences Cat# 940101 M-A261
Anti-CD27 BD Biosciences Cat# 940018 M-T271
Anti-CD272 BD Biosciences Cat# 940105 J168-540
Anti-CD274 BD Biosciences Cat# 940035 MIH1
Anti-CD273 BD Biosciences Cat# 940071 MIH18
Anti-CD275 BD Biosciences Cat# 940091 2D3/B7-H2
Anti-CD278 BD Biosciences Cat# 940043 DX29
Anti-CD279 BD Biosciences Cat# 940015 EH12.1
Anti-CD28 BD Biosciences Cat# 940017 CD28.2
Anti-CD3 BD Biosciences Cat# 940000 SK7
Anti-CD314 BD Biosciences Cat# 940061 1D11
Anti-CD33 BD Biosciences Cat# 940031 WM53
Anti-CD335 BD Biosciences Cat# 940064 9E2/NKp46
Anti-CD336 BD Biosciences Cat# 940085 p44-8
Anti-CD34 BD Biosciences Cat# 940021 563
Anti-CD39 BD Biosciences Cat# 940073 TU66
Anti-CD4 BD Biosciences Cat# 940001 SK3
Anti-CD45RA BD Biosciences Cat# 940011 HI100
Anti-CD5 BD Biosciences Cat# 940038 UCHT2
Anti-CD56 BD Biosciences Cat# 940007 NCAM16.2
Anti-CD62L BD Biosciences Cat# 940041 DREG-56
Anti-CD69 BD Biosciences Cat# 940019 FN50
Anti-CD7 BD Biosciences Cat# 940029 M-T701
Anti-CD8 BD Biosciences Cat# 940003 RPA-T8
Anti-CD94 BD Biosciences Cat# 940081 HP-3D9
Anti-CD95 BD Biosciences Cat# 940037 DX2
Anti-CD98 BD Biosciences Cat# 940093 UM7F8
Anti-TCRab BD Biosciences Cat# 940074 IP26
Anti-TCRgd BD Biosciences Cat# 940057 B1
Anti-Tim3 BD Biosciences Cat# 940066 7D3

Flow cytometry analysis of control/treated CAR-T cell products:

Anti-CD3 APC Biolegend Cat# 344812 SK7
Anti-CD4 APC-Cy7 Biolegend Cat# 317418 OKT4
Anti-CD8 Pacific Blue Biolegend Cat# 344718 SK1
Anti-CD33 FITC Biolegend Cat# 366620 P67.6
Anti-CD39 BV480 BD Biosciences Cat# 746454 TU66
Anti-CD16 PE BD Biosciences Cat# 561313 B73.1
7-AAD (Live/Dead) BD Biosciences Cat# 559925
Anti-CD4 FITC Biolegend Cat# 317408 OKT4
Streptavidin PE BD Biosciences Cat# 554061
Purified Recomb Biotinylated Protein L Therfo Fisher Scientific Cat# 29997

Flow cytometry analysis in validation cohort:

Anti-CD3 BUV563 BD Biosciences Cat# 741448 SK7
Anti-CD27 BUV661 BD Biosciences Cat# 750167 L128
Anti-CD39 PE-Fire810 Biolegend Cat# 328245 A1
CAR-T detection reagent PE Miltenyi Cat# 130-129-550
Live Dead Zombie NIR Biolegend Cat# 423105
Anti-CD16 BUV395 BD Biosciences Cat# 563785 3G8
Anti-CD14 SPARK-NIR Biolegend Cat# 399210 S18004B
Anti-CD123 AF647 Biolegend Cat# 306024 6H6
Anti-CD1c PE-Dazzle594 Biolegend Cat# 331532 L161
Anti-CD55 BV650 BD Biosciences Cat# 742680 IA10
Anti-CD69 PE-Cy5 BD Biosciences Cat# 555532 FN50

Validation

All antibodies used in this study are commercially available, broadly established, and validated by the respective manufacturers for the indicated species and applications, as detailed on their websites (see RRIDs above for each antibody). Validation information for each primary antibody includes species reactivity, specificity, and application data provided by the manufacturers.

In addition, all primary antibodies have been routinely used in our laboratory with reproducible and consistent results across multiple experiments and independent batches. This includes verification of expected staining patterns in positive control tissues/cells and the absence of non-specific staining in negative controls.

Eukaryotic cell lines

Policy information about cell lines and Sex and Gender in Research

Cell line source(s)	Nalm6 cell line and 293T cell line:the cell lines were obtained from DSMZ, German Collection of Microorganisms and Cell Cultures GmbH.
Authentication	The cell lines were authenticated through STR analyses.
Mycoplasma contamination	Cells were tested negative for mycoplasma.
Commonly misidentified lines (See ICLAC register)	Nalm6 and 293T are not listed as a misidentified or cross-contaminated cell line in the ICLAC register.

Clinical data

Policy information about clinical studies

All manuscripts should comply with the ICMJE guidelines for publication of clinical research and a completed CONSORT checklist must be included with all submissions.

Clinical trial registration	HD-CAR-1 trial was registered in the European Union Drug Regulating Authorities Clinical Trials Database (Eudra CT) (Nr. 2016-004808-60) as well as clinicaltrials.gov (NCT03676504).
Study protocol	The HD-CAR-1 trial protocol was published under:<br>Schubert ML, Schmitt A, Sellner L, Neuber B, Kunz J, Wuchter P, Kunz A, Gern U, Michels B, Hofmann S, Hückelhoven-Krauss A, Kulozik A, Ho AD, Müller-Tidow C, Dreger P, Schmitt M. Treatment of patients with relapsed or refractory CD19+ lymphoid disease with T lymphocytes transduced by RV-SFG.CD19.CD28.4-1BBzeta retroviral vector: a unicentre phase I/II clinical trial protocol. BMJ Open. 2019 May 19;9(5):e026644. doi: 10.1136/bmjopen-2018-026644. PMID: 31110096.
Data collection	In HD-CAR-1, patients were evaluated as outlined in the study calendar published under Schubert ML, et al. BMJ Open. 2019 May 19;9(5):e026644. All data were documented on case report forms (CRFs). Patient data were documented pseudonymously. To ensure data quality, regular monitoring at site was performed by an independent clinical monitor surveying completeness, validity and plausibility of data. Missing data or inconsistencies were reported back and had to be clarified by the responsible investigator prior to database lock. Data were collected in accordance to the International Council on Harmonisation of Technical Requirements for Registration of Pharmaceuticals for Human Use Harmonized Tripartite Guideline on Good Clinical Practice (GCP) (as effective by 14 June 2017). Confidentiality of data was ensured according to the European Datenschutz-Grundverordnung (DSGVO) and the German Bundesdatenschutzgesetz. The data obtained in the course of the trial were treated pursuant to the Federal Data Protection Law (Bundesdatenschutzgesetz, BDSG). During the clinical trial, enrolled patients were identified solely by means of their individual identification code (subject number, randomisation number).
Outcomes	Outcomes of HD-CAR-1 included evaluation of survival (PFS and OS), correlation of clinical response, assessment of toxicities, number of circulating gene-modified CAR T cells, assessment of reduction of disease, i.e. response and duration of response.

Plants

Seed stocks	NA
Novel plant genotypes	NA
Authentication	NA

Flow Cytometry

Plots

Confirm that:

- [x] The axis labels state the marker and fluorochrome used (e.g. CD4-FITC).
- [x] The axis scales are clearly visible. Include numbers along axes only for bottom left plot of group (a 'group' is an analysis of identical markers).
- [x] All plots are contour plots with outliers or pseudocolor plots.
- [x] A numerical value for number of cells or percentage (with statistics) is provided.

Methodology

Sample preparation	Samples were thawed at 37 °C, washed and cell suspensions resuspended in 2% FCS 0.5 mM EDTA PBS (FACS buffer) for performing staining with the antibody mix. Staining was performed at 4 °C for 30 min.
Instrument	For flow cytometric analysis, a Cytek Aurora (Cytek Biosciences) equipped with 5 lasers was used.
Software	For the acquisition of flow cytometry data, the SpectroFlo® (Cytek Biosciences, v.3.2.1) software used.
Cell population abundance	NA
Gating strategy	FSC-SSC gates were set so that FSC-low and SSC-high events were excluded (cell gate). Dead cells were removed by gating on cells low in viability dyes (Zombie NIR). Single cells (as gated based on FSC-A and FSC-H) were retained for downstream analyses.

☒ Tick this box to confirm that a figure exemplifying the gating strategy is provided in the Supplementary Information.
