# Supplementary material for: A distinct CAR-T cell phenotype mediates therapeutic response at limited doses
Source: Nat Commun. 2026 Jul 29;17:7589. doi: 10.1038/s41467-026-76068-4 (PMC13421456; doi:10.1038/s41467-026-76068-4)
Supplement: Supplementary file 6 — Transparent Peer Review file [file 41467_2026_76068_MOESM6_ESM.pdf]

nature portfolio

Peer Review File

A Distinct CAR-T Cell Phenotype Mediates Therapeutic Response at Limited Doses

Corresponding Author: Professor Simon Haas

This file contains all reviewer reports in order by version, followed by all author rebuttals in order by version.

Version 0:

Reviewer comments:

Reviewer #1

(Remarks to the Author)
In this manuscript, Yousefian and colleagues investigate determinants of therapeutic efficacy in low-dose anti-CD19 CAR T-cell therapy across multiple B-cell malignancies. Using deep immunophenotyping and single-cell proteogenomics of both leukapheresis and final CAR T-cell products, the authors propose that a CD27⁻CD39⁻ effector-like phenotype identifies therapeutically active CAR T cells capable of mediating clinical responses even at limited cell doses. This work addresses a critical challenge in cell therapy manufacturing and seeks to define correlates of response in settings of reduced CAR T-cell input.

While the findings are intriguing and potentially impactful, the manuscript would benefit from several major and minor revisions to strengthen its mechanistic foundation, interpretability, and translational relevance.

Major Comments
• Cohort Heterogeneity and Confounding by Disease Type
• The study cohort comprises patients with distinct B-cell malignancies (e.g., B-ALL, CLL, DLBCL, MCL), each with divergent immune landscapes and T-cell phenotypes. These inherent biological differences—including baseline T-cell exhaustion levels and leukapheresis composition—may confound interpretation of phenotypic signatures and response biomarkers. Stratifying analyses by disease subtype and evaluating biomarker performance within each group would improve rigor. Moreover, the high proportion of responders from B-ALL and MCL groups (versus CLL and DLBCL non-responders) raises concerns that disease biology, rather than CAR T phenotype, may drive response at low doses.

• Limited Statistical Power and Cohort Size
• The sample size, particularly within the low-dose responder subgroup (n=6), is small and limits generalizability. The statistical comparisons (e.g., Supplementary Table S2) require clarification—particularly regarding how disease subtypes and small subgroup sizes were accounted for. The possibility of confounding variables (e.g., tumor burden, myeloid cell prevalence) further limits causal inference.

• Insufficient Functional Validation of CD27⁻CD39⁻ Biomarker
• The proposed biomarker (CD27⁻CD39⁻) lacks mechanistic validation. Functional assays—such as cytotoxicity, cytokine production, serial killing, and persistence studies using sorted CD27⁻CD39⁻ CAR T cells—would significantly strengthen the central claim. Furthermore, the authors should assess biomarker stability across diseases and CAR constructs, as this study used a third-generation CAR.
•
• Myeloid Influence and Pre-Manufacture Conditions
• The authors suggest that M2-like monocytes impair CAR T-cell phenotype pre-manufacture, but do not provide functional evidence linking myeloid environment to CAR T-cell potency. Absolute counts (rather than relative abundances) and kinetic studies (e.g., washout and recovery experiments after cytokine exposure) would clarify this relationship.
•
• Ambiguity in Exhaustion and Effector Definitions
• The characterization of exhaustion based on PD1 and CD39 expression may conflate activation with dysfunction. Additional markers (e.g., CD38, Ki67, CD25, CD69) should be shown to clarify the state of these cells. The categorization of “effector-like” subsets also needs refinement, especially within CD4⁺ T-cell clusters where multiple EM-like subsets exist.

Heatmaps or supplementary figures delineating full marker expression per cluster would aid interpretation.

• Tumor Burden and Response Dynamics
• Tumor burden is a known predictor of CAR T-cell outcomes, yet corresponding data for responders vs. non-responders in the low-dose group are not presented. In addition, the duration of response and persistence in the absence of naïve or central memory cells should be addressed. The authors should comment on follow-up time and long-term outcomes.

• Inconsistencies Between Relative and Absolute Metrics
• Despite stating that absolute cell numbers are more predictive than relative frequencies (Discussion lines 444–446), the primary analyses rely heavily on relative abundance. Consistent quantification and reporting of both metrics across figures would be more appropriate.

• Minor Comments
• Clarify whether equal numbers of cells per sample were used in UMAP projections.
• Figure 1E: "Naïve-like CD8 T" is shown on a CD4 plot—please correct or explain if these are double-positive cells.
• Define "effector CD4" populations explicitly (e.g., Figure 3FG).
• Specify whether Tregs were excluded from naïve CD4 compartments in Figure 3, as their inclusion may affect observed frequencies.
• Provide details on starting T-cell numbers and expansion kinetics during manufacturing. Were phenotypic differences observed between products with higher or lower expansion?
• Include body weight, prior therapies, and disease burden metrics in the Supplementary Table for cohort characterization.
• Clarify the rationale for selecting subsets of total CD8/CD4 cells for analysis (e.g., 66k out of 10M).
• Improve data visualization by color-coding disease subtypes across figures to better interpret disease-specific trends.
• Add a heatmap or supplementary figure showing full marker expression across manually annotated populations (e.g., Figure 3A).
• Clarify whether $CD27^{-}CD39^{-}$ phenotype spans multiple subsets (e.g., $CD94^{+}$EM-like, $CD56^{+}$EM-like) and how these were defined or grouped functionally.

Overall Recommendation:
This manuscript presents a promising analysis of CAR T-cell phenotypes associated with low-dose therapeutic response. However, to fully support the proposed biomarker strategy and its clinical implications, the authors must address cohort heterogeneity, validate findings functionally, and clarify several methodological and analytical inconsistencies. With these revisions, the study would represent a meaningful advance in personalized cell therapy optimization.

Reviewer #2

(Remarks to the Author)
In this study, Yousefian et al. present a detailed phenotypic analysis of infusion products from a clinical trial evaluating a third-generation CD19-directed CAR T cell therapy with both CD28 and 4-1BB signaling domains. The focus on low dose responders is clinically relevant, especially in light of manufacturing limitations and the growing need to understand determinants of efficacy. Identifying intrinsic features of the product that correlate with treatment response in this setting is compelling.
The integration of full-spectrum flow cytometry, immune profiling, and single-cell proteogenomics provides qvaluable multidimensional insights. The authors propose that, although the relative frequencies of T cell subsets differ between low and high dose responders, the absolute numbers of functional CAR T cell subsets are comparable and may underlie clinical responses across dosing levels and show that myeloid polarization in pre aphaeresis samples may have a critical impact on CART phenotypes
While the study is ambitious and presents important findings, several critical issues must be addressed to strengthen the robustness, clarity, and impact of the conclusions:
Major Points:
1. Several groups are testing CAR T cell products in shorter cultures or enhanced with cytokines resulting in a more potent product on a per cell basis. These studies have used much lower cell numbers, for example PMID: 37249512, PMID: 40334157. In light of the demonstration that much lower cell numbers in these studies and shorter culture times producing a more potent product on a cell for cell basis, comment on the implications for the results presented in this study.
2. Does the low cell number in products reflect inefficiency in cell collection, recent lymphotoxic or lymphostatic chemotherapy, or poor expansion quality T cells, or some identifiable combination?
3. The manuscript does not report the percentage of CAR+ T cells or fold-expansion data for the infusion products, is there any correlation of CAR frequency and absolute CAR numbers with treatment response? It would be very helpful to include these metrics to enhance the interpretation.
4. The responder and non-responder groups at low doses are small (n = 6 for low-dose responders) and include diverse diseases (B-ALL, MCL, FL), while other disease types (DLBCL, CLL) are present in the low-dose non-responder and high-dose responder groups. These biological and clinical differences across groups (such as Tumor Microenvironment context, disease type or prior treatments) represent major confounding factors. Although the sample size is limited, what would restricted analysis in a single disease subtype (ie B-ALL) look like in reproducing or enhancing the reported observation. Conversely, authors should clearly acknowledge and discuss this limitation in the manuscript.
5. The findings are not validated in an independent dataset. Given the small sample sizes and high-dimensional analyses, a validation cohort would greatly improve confidence in the reported findings.
6. The authors present an interesting association between M2-like pre-apheresis myeloid polarization and dysfunctional

CART phenotypes. To functionally validate the impact of myeloid polarization on CAR T cell quality, the authors should consider complementing cytokine -based assays with co-culture experiments using in vitro generated M1 and M2 macrophages. This would more closely replicate physiologic conditions and enable assessment of downstream CAR-T phenotypes (CD27, CD39 expression, memory/exhaustion states and or cytotoxic potential). In vitro and/or in vivo testing of CAR T cells expanded after these preconditioning conditions would further enhance the translational relevance.
Minor points
The authors state that "manual annotation of clusters was conducted based on marker expression and expert knowledge." Please specify which markers were used to define key populations.
Line 87: Capitalize "a" in "figure 1a."
The manuscript does not address how the third-generation CAR design (CD28 + 4-1BB signaling) may influence observed T cell phenotypes. This is important, as costimulatory domains are known to shape expansion kinetics, exhaustion resistance, and memory retention. Please discuss.
Please clarify the statistical approach used for calculating "comparable" absolute numbers of CAR-T subsets between low- and high-dose responders.

Reviewer #3

(Remarks to the Author)
I co-reviewed this manuscript with one of the reviewers who provided the listed reports. This is part of the Nature Communications initiative to facilitate training in peer review and to provide appropriate recognition for Early Career Researchers who co-review manuscripts.

Version 1:

Reviewer comments:

Reviewer #1

(Remarks to the Author)
Overall, the authors have mostly addressed the reviewers' concerns with substantive additions rather than purely rhetorical edits. The revision includes meaningful new analyses to reduce confounding, clearer statistical methodology, added functional validation of the proposed biomarker subset, and an independent external cohort that supports generalizability. A small number of issues remain only partially resolved due to structural constraints (sample size, heterogeneity, and limited access to commercial product material).
Major concerns and how they were addressed
1) Disease heterogeneity/ confounding by indication
The authors added disease-subtype stratified analyses (composition differences, baseline phenotype comparisons, effect-size screening of covariates, and subtype-stratified biomarker performance). These analyses support that the key signal is not simply an artifact of pooled disease types. This addresses the confounding concern to a large extent, though subgroup Ns necessarily limit statistical decisiveness.
2) Statistical clarity and small-sample handling
They substantially clarified the statistical framework (univariable modeling appropriate for sparse data, explicit handling of separation, and correlation/association matrices to assess confounding). This improves transparency and reduces analytic ambiguity. Power limitations remain inherent but are acknowledged and appropriately handled without overfitting.
3) Functional validation of the $CD27^{-}CD39^{-}$ subset
A key improvement is the addition of sorted-subset functional experiments (sequential co-culture challenges, expansion/phenotypic evolution readouts, residual tumor quantification, and cytokine profiling). This directly strengthens the mechanistic plausibility of the proposed biomarker by linking phenotype to functional performance in vitro. While the model remains simplified, this is a substantive response to the request for functional validation.
4) Myeloid/cytokine milieu and reversibility ("washout")
The authors added cytokine exposure plus washout experiments demonstrating that induction of the implicated dysfunction-associated phenotype is reversible/preventable and that functional impairment recovers after washout. This is a strong, targeted response to concerns about causal linkage and kinetic plausibility, with the caveat that it remains a reductionist proxy for in vivo myeloid effects.
5) Generalizability across CAR constructs/ products
They added an independent validation cohort spanning multiple commercial products and targets. This materially improves confidence that the signal is not restricted to a single CAR architecture. However, the cohort relies on early post-infusion blood as a proxy phenotype rather than direct infusion-product profiling, which should be viewed as validation of early on-treatment behavior rather than manufacturing phenotype per se.
Partially addressed/ residual issues
Tumor burden and outcome durability in the low-dose subgroup:
The authors provide survival analyses and use LDH as a burden proxy due to mixed malignancies. This is a reasonable workaround but remains an imperfect substitute for standardized volumetric tumor burden measures and is underpowered in low-dose subgroup analyses.
Activation vs dysfunction ambiguity and Treg contamination:
They improved transparency with expanded marker visualization and more cautious cluster naming, but surface-marker panels cannot fully resolve activation-versus-durable dysfunction without orthogonal evidence. Treg concerns are mitigated with proxy gating approaches but remain inferential without intracellular markers.

Reviewer #2

(Remarks to the Author)
The authors have made a substantial and good-faith effort to address initial concerns and have added extensive new analyses and functional experiments that significantly strengthen the manuscript. Overall, the study now represents a high-quality translational contribution with important implications for CAR T-cell biomarker development and manufacturing optimization. However, several central conclusions remain overstated relative to the data and require further refinement. Additional experiments would not be strictly necessary, provided the interpretation and abstract are appropriately revised. However, if the authors wish to further strengthen mechanistic claims, a limited in vivo assessment of product-level potency could be informative.

1. First, the authors have made a commendable effort to validate the proposed CD27-CD39- biomarker in an independent cohort spanning multiple diseases, CAR constructs, targets, and commercially approved products. These analyses support the generalizability of the CD27-CD39- phenotype as a *correlate* of clinical response. However, due to practical limitations, validation was performed using early post-infusion peripheral blood samples rather than manufactured infusion products. As such, these data primarily reflect early in vivo CAR T-cell dynamics rather than intrinsic properties of the infusion product itself. Consequently, while the biomarker appears predictive of response, the data do not fully support claims that product-intrinsic features determine therapeutic efficacy, particularly in the context of low-dose CAR T-cell therapy. This limitation should be explicitly acknowledged in the manuscript.
2. The authors performed well-executed functional assays using sorted CAR T-cell subsets, including killing assays, phenotypic stability analyses, and cytokine profiling. These data convincingly demonstrate that CD27-CD39- CAR T cells represent a stable, terminal effector state with high per-cell cytotoxic efficiency. At the same time, the experiments that authors performed also show that CD27+CD39+ CAR T cells exhibit greater proliferative capacity and can give rise to CD27-CD39- progeny, contributing substantially to overall tumor clearance. Thus, while the biomarker identifies a biologically meaningful effector state associated with response, it does not uniquely define a superior or exclusive driver of therapeutic efficacy. Importantly, no experiments directly test the necessity or sufficiency of the CD27-CD39- population at the product level (depletion/add-back or limiting-dose in vivo transfer), nor is an in vivo persistence experiment performed in preclinical models where advantage is proved or demonstrated. Accordingly, the authors should either moderate their conclusions to reflect this limitation or, alternatively, perform in vivo studies to directly assess the relative potency of these subsets. If such in vivo experiments are not undertaken, the authors should explicitly acknowledge that comparative in vivo potency studies would further strengthen the mechanistic interpretation and therefore represent a limitation of the current work.
3. The authors added thoughtful mechanistic follow-up experiments using M2 macrophage co-culture and cytokine washout approaches. These data show that sustained exposure to suppressive cytokines during manufacturing can transiently impair CAR T-cell function and induce CD39 expression, and that these effects are reversible upon removal of suppressive signals. Notably, transient exposure to M2 macrophages alone did not durably impair CAR T-cell quality. These findings are consistent with prior literature on cytokine-mediated T-cell suppression and support a process-dependent and reversible mechanism rather than a fixed, patient-intrinsic determinant. Accordingly, conclusions should be moderated, and the abstract should be revised to accurately reflect this.
4. This statement in the response is incorrect: “as reflected by current commercial practice, manufacturers (e.g. Novartis) have reverted to longer, more conventional culture durations for approved products” Kymriah and other CAR T products, as approved, have a culture period that is specified by each regulatory product approved and have not been shortened (and could not without supplemental regulatory filings). Each commercial developer is advancing shorter products in clinical trials with the intent for regulatory submissions. The implication that these shorter culture products will be harder to implement is in this reviewer’s judgement, incorrect.

Reviewer #3

(Remarks to the Author)
I co-reviewed this manuscript with one of the reviewers who provided the listed reports. This is part of the Nature Communications initiative to facilitate training in peer review and to provide appropriate recognition for Early Career Researchers who co-review manuscripts.

Open Access This Peer Review File is licensed under a Creative Commons Attribution 4.0 International License, which permits use, sharing, adaptation, distribution and reproduction in any medium or format, as long as you give appropriate credit to the original author(s) and the source, provide a link to the Creative Commons license, and indicate if changes were made.
In cases where reviewers are anonymous, credit should be given to 'Anonymous Referee' and the source.
The images or other third party material in this Peer Review File are included in the article's Creative Commons license, unless indicated otherwise in a credit line to the material. If material is not included in the article's Creative Commons license and your intended use is not permitted by statutory regulation or exceeds the permitted use, you will need to obtain permission directly from the copyright holder.
To view a copy of this license, visit https://creativecommons.org/licenses/by/4.0/

Reviewer #1 (Remarks to the Author):

In this manuscript, Yousefian and colleagues investigate determinants of therapeutic efficacy in low-dose anti-CD19 CAR T-cell therapy across multiple B-cell malignancies. Using deep immunophenotyping and single-cell proteogenomics of both leukapheresis and final CAR T-cell products, the authors propose that a $CD27^{-}CD39^{-}$ effector-like phenotype identifies therapeutically active CAR T cells capable of mediating clinical responses even at limited cell doses. This work addresses a critical challenge in cell therapy manufacturing and seeks to define correlates of response in settings of reduced CAR T-cell input.

While the findings are intriguing and potentially impactful, the manuscript would benefit from several major and minor revisions to strengthen its mechanistic foundation, interpretability, and translational relevance.

We thank the reviewer for the thoughtful summary of our work and for recognizing the potential impact of defining therapeutically active CAR-T cell subsets in the context of low-dose anti-CD19 therapy across B-cell malignancies. We fully agree that strengthening the mechanistic foundation, interpretability, and translational relevance of our findings is essential. In response, we have revised the manuscript to better contextualize the CD27-CD39- effector-like phenotype, performed functional validation experiments and validated our biomarker combination in an independent patient cohort comprising both anti-CD19 and anti-BCMA CAR-T cell constructs. Detailed point-by-point responses to all major and minor comments are provided below.

Major Comments

Cohort Heterogeneity and Confounding by Disease Type

• The study cohort comprises patients with distinct B-cell malignancies (e.g., B-ALL, CLL, DLBCL, MCL), each with divergent immune landscapes and T-cell phenotypes. These inherent biological differences—including baseline T-cell exhaustion levels and leukapheresis composition—may confound interpretation of phenotypic signatures and response biomarkers. Stratifying analyses by disease subtype and evaluating biomarker performance within each group would improve rigor. Moreover, the high proportion of responders from B-ALL and MCL groups (versus CLL and DLBCL non-responders) raises concerns that disease biology, rather than CAR T phenotype, may drive response at low doses.

We thank the reviewer for raising this important point. We fully agree that the inclusion of patients with different B-cell malignancies can introduce biological heterogeneity that may influence both CAR-T cell phenotype and clinical outcome. To better understand whether, and to what extent, disease subtype contributes to the observed phenotypic signatures and identified biomarkers, we performed additional analyses (Figure R1). First, we examined whether disease subtypes differ in cellular

composition at the time of leukapheresis (Figure R1A). We observed largely similar cellular compositions across subtypes, with no clear clustering by disease in a principal component analysis based on the respective cell type frequencies. We then assessed baseline CD8 and CD4 T cell exhaustion markers per disease subtype (Figure R1B,C), revealing only minor differences with none of them displaying a significant enrichment in any specific disease group at leukapheresis.

To more systematically determine which variables and potential confounders are explained by disease subtype and might therefore affect biomarker signatures, we performed a global η^2 (eta squared) analysis (Figure R1D). Specifically, we tested each numeric variable and potential confounder using a one-way ANOVA with disease subtype as the independent factor and calculated η^2 as an effect size, quantifying the proportion of variance in each variable attributable to disease subtype. Across all tested potential confounding variables, we observed only moderate η^2 values, indicating that disease subtype explains only a limited fraction of the variability in these features. Because these baseline characteristics did not show strong heterogeneity across disease subtypes, we subsequently evaluated whether T cell/CAR-T cell exhaustion after manufacturing, as reflected by CD39 expression, was influenced by disease subtype (Figure R1E). In both the CD8 and CD4 CAR-T cell compartments, we did not observe a discernible effect of disease subtype on CD39 expression levels in the infusion product. Finally, we assessed biomarker performance stratified by disease subtype (Figure R1G). Importantly, in all disease groups the CD27-CD39- CAR-T cell phenotype was enriched in responders compared to non-responders. However, due to the reduced sample size in each group, statistical significance was only reached in some disease groups.

Together with the positive validation results in an independent cohort (see Figure R4 below), these analyses suggest that while disease-specific biology may contribute to overall outcome differences in a minor fashion, our CD27-CD39- biomarker combination is not primarily driven by disease subtype and appears to capture a cross-disease CAR-T cell feature associated with response at low CAR-T cell doses. These results have now been implemented as Supplementary Figure 7.

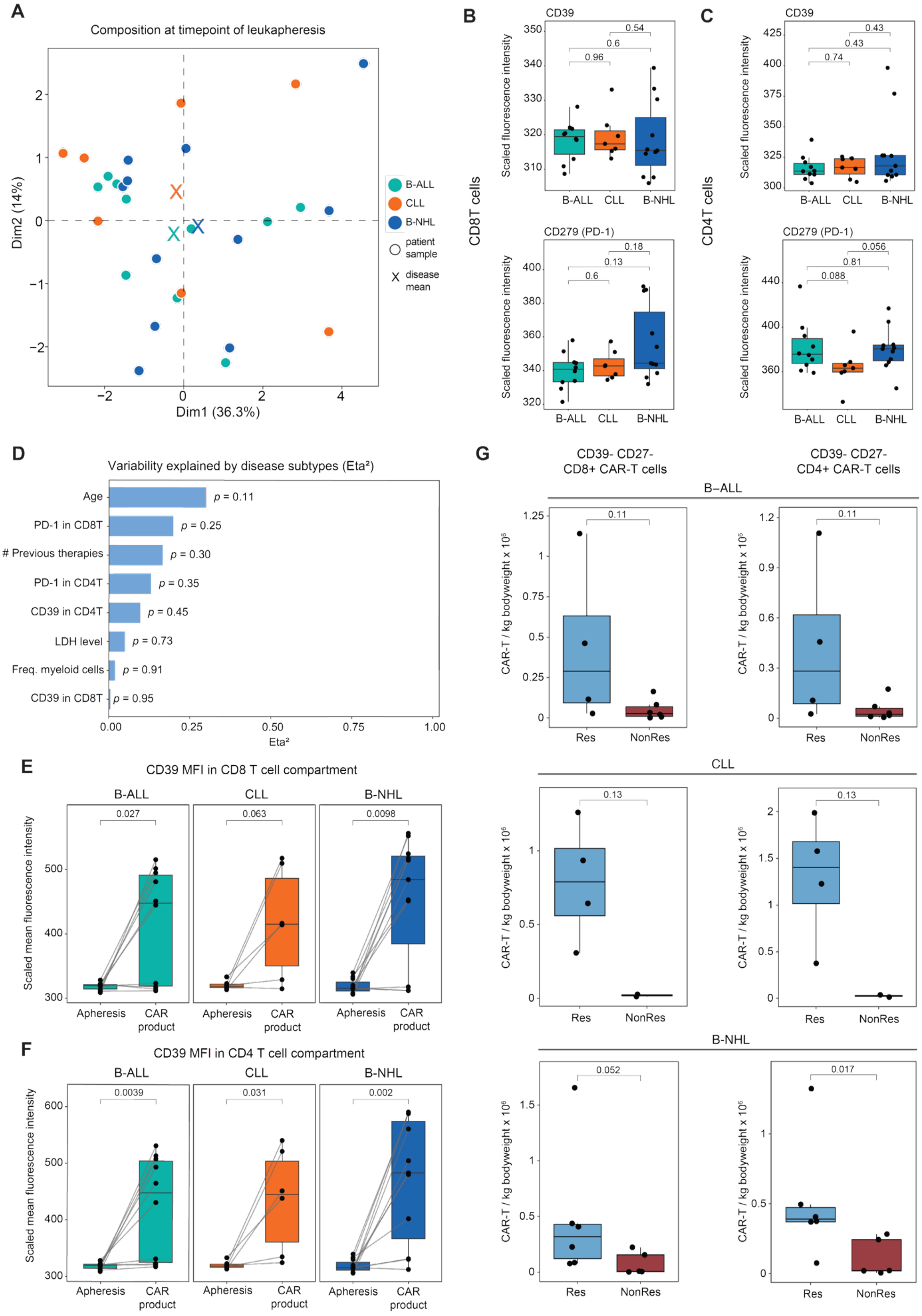

Figure R1. Cohort heterogeneity and individual disease subtype analyses. A. Composition analysis using principal component analysis (PCA) of PBMCs at timepoint of

leukapheresis, stratified by disease subtype. **B.** Scaled fluorescence intensity of CD39 and CD279 (PD-1) in CD8 T cells at apheresis, stratified by disease subtype. P values were determined with a two-sided Wilcoxon rank-sum test. n = 27. **C.** Scaled fluorescence intensity of CD39 and CD279 (PD-1) in CD4 T cells at apheresis, stratified by disease subtype. P values were determined with a two-sided Wilcoxon rank-sum test. n = 27. **D.** Global η^2 (eta-squared) analysis of numeric variables by disease subtype using a one-way ANOVA test. P values are adjusted using Benjamini-Hochberg. **E.** Paired CD39 fluorescence intensity in CD8 T cells at apheresis versus CAR-T cell product across disease subtypes. P values were determined with a paired two-sided Wilcoxon rank-sum test. n = 27. **F.** Paired CD39 fluorescence intensity in CD4 T cells at apheresis versus CAR-T cell product across disease subtypes. P values were determined with a paired two-sided Wilcoxon rank-sum test. n = 27. **G.** Absolute numbers of CD8+CD27-CD39- and CD4+CD27-CD39- biomarker populations stratified by disease subtype. P values were determined with a two-sided Wilcoxon rank-sum test. B-ALL n = 10; CLL n =6; B-NHL n = 11.

Limited Statistical Power and Cohort Size

• The sample size, particularly within the low-dose responder subgroup (n=6), is small and limits generalizability. The statistical comparisons (e.g., Supplementary Table S2) require clarification—particularly regarding how disease subtypes and small subgroup sizes were accounted for. The possibility of confounding variables (e.g., tumor burden, myeloid cell prevalence) further limits causal inference.

We thank the reviewer for this thoughtful comment and fully agree that the limited cohort size, particularly in the low-dose responder subgroup, constrains statistical power and generalizability. We also appreciate the need to clarify the statistical framework used in Supplementary Table S2. In the revised manuscript, we now explicitly describe in the Methods section how odds ratios were calculated and how disease subtype and dose categories were incorporated, and we have updated Supplementary Table S2 accordingly. Given the small subgroup sizes, we refrained from fitting complex multivariable models including many covariates, as such models would be statistically underpowered and unstable. Instead, we focused on robust univariable and correlation-based analyses to explore potential confounding factors.

To clarify the analyses underlying Supplementary Table S2, now also visualized in Figure R2A and implemented as Supplementary Figures 1A and 4B, we performed univariable logistic regression with clinical response (Responder vs. Non-Responder) as the binary outcome and each clinical or biological covariate as a predictor. For each variable, we estimated odds ratios (ORs) with 95% confidence intervals. Because of the small sample size and sparse data in some categories, we used a stepwise strategy to obtain stable odds ratio estimates. We first ran standard logistic regression models. If these failed due to separation (i.e., one outcome occurring only in one group), we used Firth's penalized logistic regression, an approach specifically designed to handle small samples and separation in logistic regression. If this was still not applicable, we finally applied a Haldane-Anscombe continuity correction (adding 0.5 to each cell of the 2×2 table) to calculate finite odds ratios and confidence intervals.

P-values were obtained from Fisher's exact tests and adjusted for multiple testing. In these analyses, we observed that tumor burden, approximated by lactate dehydrogenase (LDH) levels, had a moderate, non-significant association with clinical outcome. In contrast, our biomarker combination was significantly associated with clinical response, both when computed separately for the CD4 or CD8 CAR-T cell compartments. A composite variable combining LDH level with the biomarker did not further strengthen this association (Table in Figure R2A), suggesting that our biomarker combination is not simply a surrogate for LDH-defined tumor burden.

To further explore the relationships between potential confounders and our biomarker combination, we computed pairwise Pearson correlations across all numeric covariates and the biomarkers (Figure R2B). We did not observe meaningful correlations between the biomarkers and other numeric covariates, but we did detect strong correlations between our biomarker signatures when derived from the CD4 and CD8 CAR-T cell compartments, indicating that these two features capture related biological information. In addition, to characterize relationships between categorical covariates and to assess potential collinearity, we computed pairwise associations among all categorical variables, including clinical response. For each pair, we constructed contingency tables and quantified the strength of association using Cramér's V, yielding a matrix of association coefficients ranging from 0 to 1 (Figure R2C). As expected, we found the strongest association between our biomarker combinations and clinical response. Other covariates showed only weak to moderate associations with either the CD4/CD8 biomarker combination or clinical response, arguing against individual dominant categorical confounders driving the observed biomarker-response relationship.

Taken together, these additional analyses indicate that, within the constraints of our limited cohort size, the association between the biomarker combination and clinical response is not readily explained by the examined clinical and biological confounders, although we acknowledge that validation in larger, independent datasets will be essential to confirm these findings, which we will address below (see response to this reviewer's point below and Figure R4).

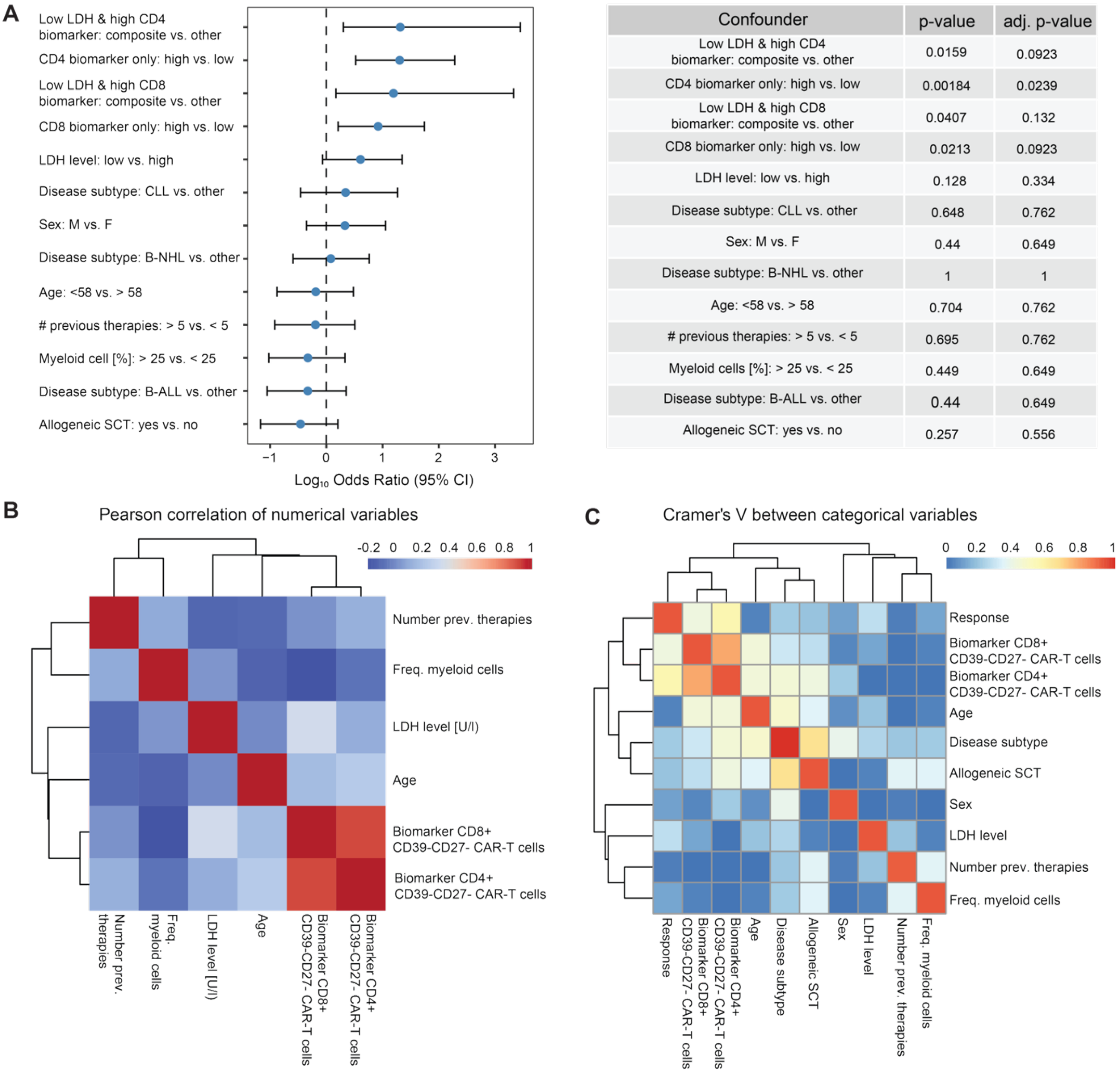

Confounder	p-value	adj. p-value
Low LDH & high CD4 biomarker: composite vs. other	0.0159	0.0923
CD4 biomarker only: high vs. low	0.00184	0.0239
Low LDH & high CD8 biomarker: composite vs. other	0.0407	0.132
CD8 biomarker only: high vs. low	0.0213	0.0923
LDH level: low vs. high	0.128	0.334
Disease subtype: CLL vs. other	0.648	0.762
Sex: M vs. F	0.44	0.649
Disease subtype: B-NHL vs. other	1	1
Age: <58 vs. > 58	0.704	0.762
# previous therapies: > 5 vs. < 5	0.695	0.762
Myeloid cells [%]: > 25 vs. < 25	0.449	0.649
Disease subtype: B-ALL vs. other	0.44	0.649
Allogeneic SCT: yes vs. no	0.257	0.556

Figure R2. Association between biomarkers and potential confounders. A. Forest plot with odds ratios for the association between clinical response and clinical or biological covariates. P values were obtained from Fisher's exact tests and adjusted for multiple comparisons using the Benjamini-Hochberg correction. **B.** Pairwise Pearson correlation matrix of numeric covariates and the CD4/CD8 biomarker combination. Color scale indicates correlation coefficients (-0.2 - 1). **C.** Pairwise Cramér's V association matrix of categorical covariates, including clinical response. Values range from 0 (no association) to 1 (strong association).

Insufficient Functional Validation of $CD27^{-}CD39^{-}$ Biomarker

• The proposed biomarker ($CD27^{-}CD39^{-}$) lacks mechanistic validation. Functional assays—such as cytotoxicity, cytokine production, serial killing, and persistence studies using sorted $CD27^{-}CD39^{-}$ CAR T cells—would significantly strengthen the central claim. Furthermore, the authors should assess biomarker stability across diseases and CAR constructs, as this study used a third-generation CAR.

We thank the reviewer for this important comment. We fully agree that both mechanistic validation of the CD27-CD39- subset and assessment of biomarker stability across diseases and CAR constructs are critical to strengthen our central claim. In response, we have performed functional in vitro experiments to directly assess the activity of CD27-CD39- CAR-T cells (Figure R3) and, in addition, validated our biomarker combination in an independent patient cohort encompassing different diseases (B-cell non-Hodgkin's lymphoma (B-NHL) and multiple myeloma (MM)), CAR constructs (a variety of second-generation designs in addition to the third-generation of the previously presented data), and CAR targets (anti-CD19, anti-BCMA) (Figure R4).

To characterize the functional properties of the CD27-CD39- CAR-T cell population, we performed two rounds of co-culture assays using sorted CAR-T cells from six healthy donors and Nalm6 leukemia cells (B-cell precursor leukemia cell line). We compared the CD27-CD39- CAR-T cell subset with CD27+CD39+ CAR-T cells, which served as a phenotypic "opposite" control to our proposed biomarker population. After 72 hours of co-culture at a 1:1 effector-to-target ratio, we harvested the cells and repeated a second 72-hour co-culture round. Across these two sequential co-cultures, we quantified phenotypic plasticity, CAR-T cell expansion, and residual leukemia cell counts as a measure of cytotoxic efficacy. Consistent with their classification as terminal effector cells, CD27-CD39- CAR-T cells retained their immunophenotype and exhibited minimal plasticity. In contrast, the CD27+CD39+ CAR-T cell population gave rise to a diverse range of CAR-T cell subsets, including CD27-CD39- cells, indicating greater phenotypic plasticity. Analysis of CAR-T cell expansion revealed that CD27+CD39+ cells proliferated robustly during the first co-culture round, more than doubling in number, whereas CD27-CD39- CAR-T cells showed only limited expansion, being consistent with their effector phenotype (Figure R3A, B).

When cytotoxicity was assessed by quantifying residual leukemia cells, both subsets displayed potent leukemia-killing activity compared to non-transduced controls. Notably, CD27+CD39+ CAR-T cells eliminated a slightly higher absolute number of leukemia cells, likely reflecting their greater early expansion and the emergence of CD27-CD39- progeny. However, when normalized on a per CAR-T cell basis - accounting for both CAR-T cell proliferation and leukemia cell growth over the 72-hour period - the CD27-CD39- subset demonstrated higher killing efficiency in the first co-culture round (Figure R3C). In the second round, per CAR-T cell killing was similar between the CD27-CD39- and CD27+CD39+ starting conditions (Figure R3D). This is likely due to the fact that, by the end of the first co-culture, a large fraction of CD27+CD39+ CAR-T cells had converted to a CD27-CD39- phenotype, so that both cultures were now dominated by CD27-CD39- CAR-T cells. As a consequence, the phenotypic convergence of the two populations reduces detectable differences in per-cell cytotoxicity in the second killing round. To obtain further mechanistic insights into CD27-CD39- CAR-T cell function, we performed multiplex Olink-based cytokine profiling of supernatants collected from the CAR-T-leukemia cell co-cultures (Figure

R3E). When comparing each CAR-T cell subset to the non-transduced control, we observed enrichment of key effector cytokines, including IFN-γ, TNF, and IL-2, consistent with robust cytotoxic activity. In contrast, direct comparison of CD27+CD39+ and CD27-CD39- CAR-T cells did not reveal cytokines that were selectively or significantly enriched in one subset over the other.

Together, these findings support the interpretation that CD27-CD39- CAR-T cells represent a highly functional, terminal effector subset characterized by stable phenotype, limited plasticity, and high cytotoxic potency. These results have now been implemented as Figure 3 and Supplementary Figure 5 of the manuscript.

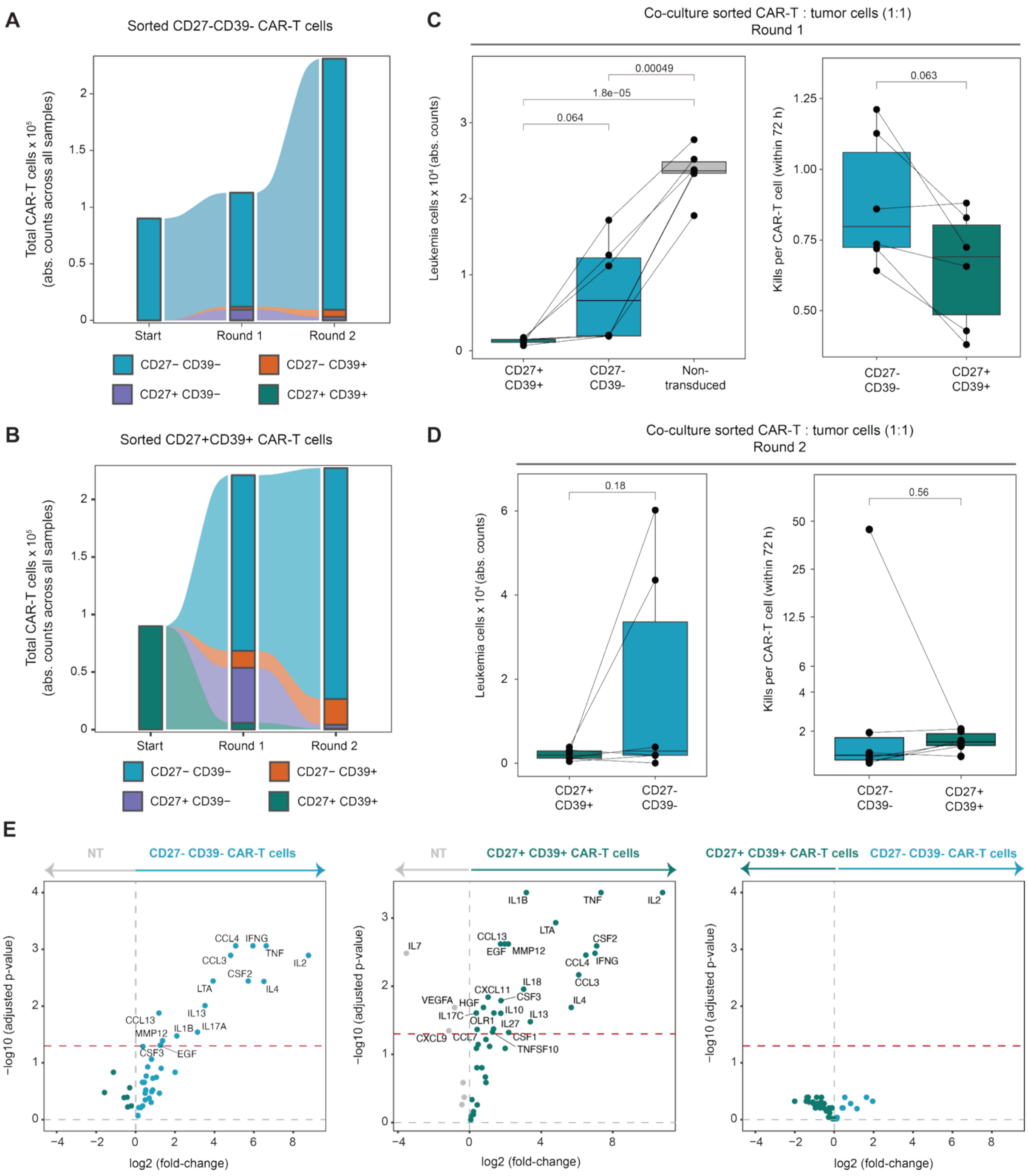

Figure R3. CD27-CD39- CAR-T cells represent a terminal effector subset with limited plasticity and high cytotoxic potency. A. Alluvial plot showing CAR-T cell expansion (absolute cell numbers summed across all six healthy donors) from sorted CD27-CD39- CAR-T cells across two sequential co-culture rounds with Nalm6 leukemia cells. **B.** Alluvial plot showing CAR-T cell expansion (absolute cell numbers summed across all six healthy donors) from sorted CD27+CD39+ CAR-T cells across two sequential co-culture rounds with Nalm6 leukemia cells. **C.** Left: quantification of residual leukemia cells after the first co-culture round. P values were determined with a paired, two-sided t-test. n = 6. Right: per CAR-T cell killing efficiency in round 1, normalized for total CAR-T cell abundance and leukemia cell growth. P values were determined with a paired, two-sided Wilcoxon rank-sum test. n = 6. **D.** Left: quantification of residual leukemia cells after the second co-culture round. P values were

determined with a paired, two-sided t-test. n = 6. Right: per CAR-T cell killing efficiency in round 2, normalized for total CAR-T cell abundance and leukemia cell growth. P values were determined with a paired, two-sided Wilcoxon rank-sum test. n = 6. **E.** Olink-based cytokine profiling of supernatants from CAR-T-leukemia cell co-cultures after the first co-culture round. Left: non-transduced vs CD27-CD39- CAR-T cells. Middle: non-transduced vs CD27+CD39+ CAR-T cells. Right: CD27+CD39+ vs CD27-CD39- CAR-T cells. P values were determined with paired, two-sided t-tests and adjusted using the Benjamini-Hochberg procedure. Red dashed lines indicate the significance threshold.

To assess the robustness and generalizability of our proposed biomarker combination beyond our initial cohort and CAR design, we next evaluated its association with clinical response in an independent patient cohort spanning multiple diseases (B-cell non-Hodgkin's lymphoma [B-NHL] and multiple myeloma [MM]), CAR constructs (second-generation CARs with either CD28 or 4-1BB co-stimulatory domains), and targets (anti-CD19, anti-BCMA) (Figure R4). Patients in this validation cohort were treated with four different commercial CAR-T cell products: Breyanzi (anti-CD19, Bristol Myers Squibb), Yescarta (anti-CD19, Kite/Gilead Sciences), Kymriah (anti-CD19, Novartis), and Carvykti (anti-BCMA, Janssen Biotech/Legend Biotech).

Because these were commercial infusion products, we were legally not permitted to perform direct analyses on the CAR-T cell products themselves. We therefore measured CD27 and CD39 expression of CAR-T cells in the first peripheral blood sample obtained after infusion (between day 1 and day 10 for each patient) and used this early time point as a proxy readout of the CAR-T cell product, CAR-T cell activity and biomarker expression *in vivo*. In total, we analyzed samples from 17 MM and 25 B-NHL patients (Figure R4A). In addition, to validate our previously observed myeloid phenotype in pre-manufacturing PBMCs, we assessed CD55 and CD69 expression on PBMCs collected at the time of leukapheresis. As expected for commercially approved anti-CD19 CAR-T cell therapies, the infused CAR-T cell doses were higher than in our phase I/II dose-escalation study. Importantly, however, all MM patients received the same CAR-T cell dose according to the end-product specifications (Figure R4B). In this setting, we found that the biomarker combination measured in CAR-T cells in the first post-infusion PBMC sample could stratify patients into responders and non-responders when using the absolute number of CD27-CD39- CAR-T cells (Figure R4C). Given the relatively similar infused doses within product groups, the relative frequency of CD27-CD39- CAR-T cells could also discriminate between responders and non-responders (Figure R4D), indicating that within a comparable dose range, both absolute counts and relative frequencies of the biomarker population carry predictive information. When comparing our proposed biomarker to previously reported signatures for anti-CD19 CAR-T cells, our CD27-CD39- combination outperformed these alternatives in patients treated with CD19-directed CAR-T cells in terms of both sensitivity and specificity for clinical response (Figure R4E).

Finally, we validated our biomarkers associated with myeloid cells in the premanufacturing blood, including CD55 and CD69 (Figure R4F). Fully recapitulating our initial findings, CD55 expression on non-classical monocytes was higher in non-responding patients, whereas CD69 expression was predominantly enriched on conventional dendritic cells (cDCs) and classical monocytes from responders at the timepoint of leukapheresis (before CAR-T cell manufacturing).

Taken together, these validation data support that the CD27-CD39- biomarker combination is reproducibly associated with clinical response across different diseases, and CAR constructs, suggesting that it captures a core biological property of highly potent and therapeutically effective CAR-T cells rather than a cohort- or product-specific effect. We acknowledge, however, that these validation analyses were performed on CAR-T cells in early post-infusion peripheral blood samples rather than on the infusion products themselves, which represents an important limitation that will need to be addressed in future studies. Moreover, we could validate biomarkers within the myeloid compartment at the time before CAR-T cell generation. These results have now been implemented as Figure 7 of the main manuscript.

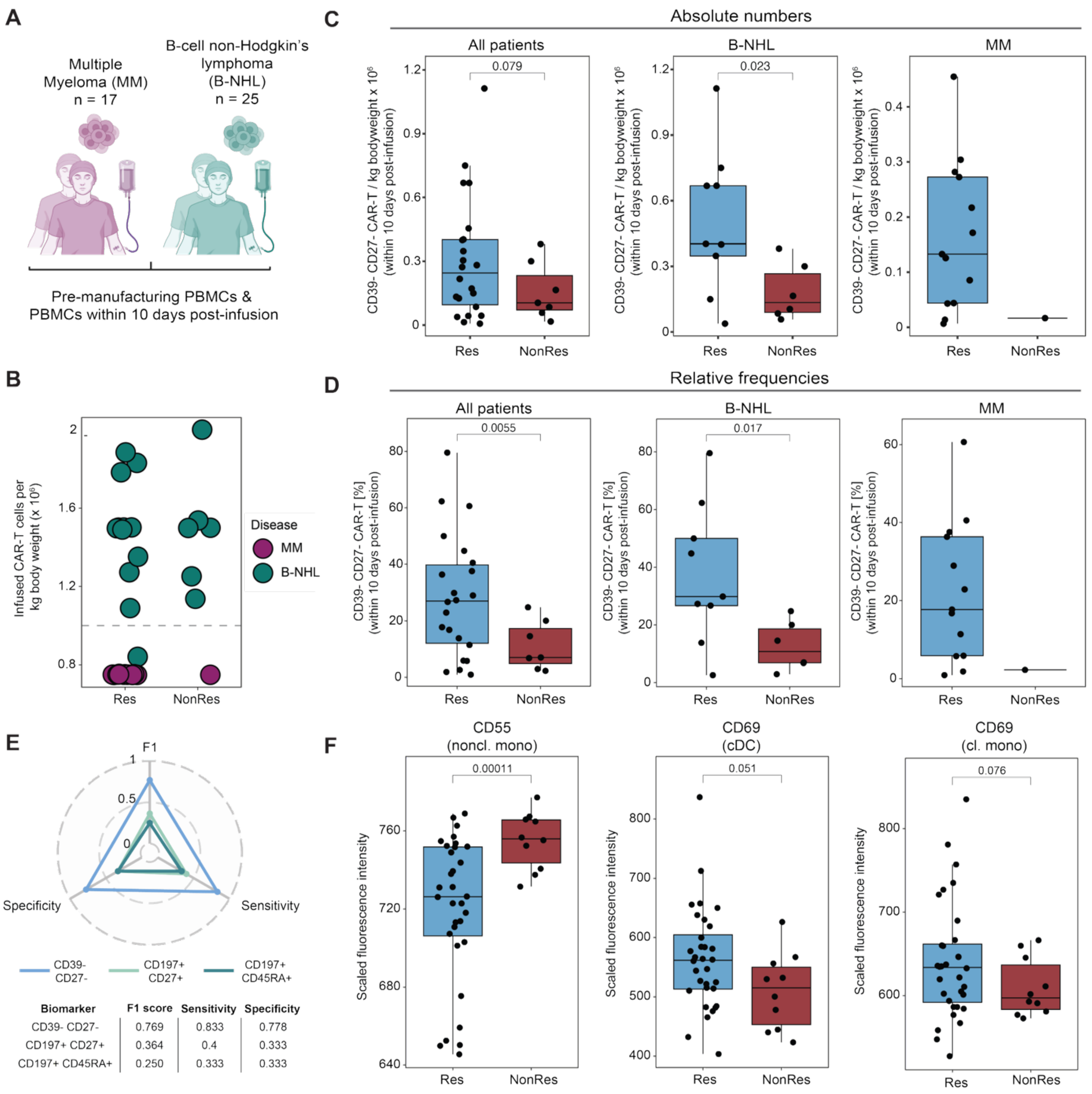

Figure R4. Validation of the CD27-CD39- biomarker across an independent patient cohort. A. Overview of patients in the validation cohort (n = 42). PBMCs at the time of apheresis and CAR-T cells at a single post-infusion time point between day 1 and day 10 were analysed. **B.** Infused number of CAR-T cells per kg body weight. The grey dashed line indicates the threshold used to define low-dose patients in the original cohort. **C.** Absolute number of CD27-CD39- CAR-T cells in the first post-infusion PBMC sample across all patients (left, n = 29), B-NHL (middle, n = 15), and MM (right, n = 14). P values were determined with a two-sided Welch's t-test. **D.** Relative frequency of CD27-CD39- CAR-T cells in the first post-infusion PBMC sample across all patients (left, n = 29), B-NHL (middle, n = 15), and MM (right, n = 14). P values were determined with a two-sided Welch's t-test. **E.** Comparative evaluation of different biomarker signatures using F1-score, specificity, and sensitivity. **F.** Mean fluorescence intensity of CD55 and CD69 in non-classical monocytes (noncl. mono), classical dendritic cells (cDC), and classical monocytes (cl. mono). P values were determined with a two-sided Welch's t-test (n = 42).

Myeloid Influence and Pre-Manufacture Conditions

• The authors suggest that M2-like monocytes impair CAR T-cell phenotype pre-manufacture, but do not provide functional evidence linking myeloid environment to CAR T-cell potency. Absolute counts (rather than relative abundances) and kinetic studies (e.g., washout and recovery experiments after cytokine exposure) would clarify this relationship.

We thank the reviewer for this insightful comment and fully agree that more functional evidence is required to link the myeloid environment to CAR-T cell potency, and that "washout" experiments are particularly informative to assess whether these are transient or rather long-lasting effects. To address this, we repeated the in vitro pre-treatment experiments previously shown in Figure 5 of the main manuscript and extended them with an additional washout condition. PBMCs were exposed to a cytokine mix designed to mimic an M2-like myeloid environment and were then used for CAR-T cell manufacturing under three distinct pre-treatment conditions:

1. PBMCs without cytokine pre-treatment (control),
2. PBMCs pre-treated with the cytokine mix (to mimic an M2-like myeloid environment) without extensive washout, and
3. PBMCs pre-treated with the cytokine mix followed by extensive washout before CAR-T cell manufacturing was initiated.

As expected and consistent with our original data, the frequency of CD39+ CAR-T cells increased significantly in the condition with M2-related cytokine pre-treatment and no washout compared with the untreated control (Figure R5A). In contrast, when cytokines were washed out before manufacturing, CD39+ CAR-T cell frequencies remained at levels comparable to the untreated control, indicating that the cytokine-driven upregulation of CD39 is largely transient and can be prevented by removing the myeloid-like cytokine milieu prior to CAR-T cell generation. We then addressed whether this phenotypic "reversal" translated into restored cytotoxic effector function. Using co-cultures of CAR-T cells and leukemia cells at a 1:1 effector-to-target ratio, we quantified the absolute number of residual leukemia cells after co-culture. As expected, and in line with our original data (Figure 5E of the main manuscript), CAR-T cells derived from M2-related cytokine pre-treated PBMCs without washout showed reduced killing, reflected by higher absolute numbers of remaining leukemia cells compared with the control. In contrast, CAR-T cells derived from the washout condition displayed leukemia cell killing comparable to the untreated control, indicating that reversal of the cytokine milieu before manufacturing prevents the functional impairment of CAR-T cells (Figure R5B). Finally, Spearman correlation analysis between the percentage of CD39+ CAR-T cells and the absolute number of residual leukemia cells demonstrated a significant positive association, supporting the notion that the degree of CD39 expression is linked to reduced CAR-T cell cytotoxicity in vitro (Figure R5C).

Taken together, these additional experiments provide functional support that an M2-like myeloid cytokine environment can transiently impair CAR-T cell phenotype and killing capacity, and that this effect is at partially reversible when the cytokine/myeloid influence is removed prior to CAR-T cell manufacturing. Although this in vitro system is a simplified model of the in vivo myeloid compartment, it strengthens the mechanistic link between the myeloid milieu, CD39 upregulation, and diminished CAR-T cell potency inferred from our patient data. These results have now been implemented in Figure 6 of the manuscript.

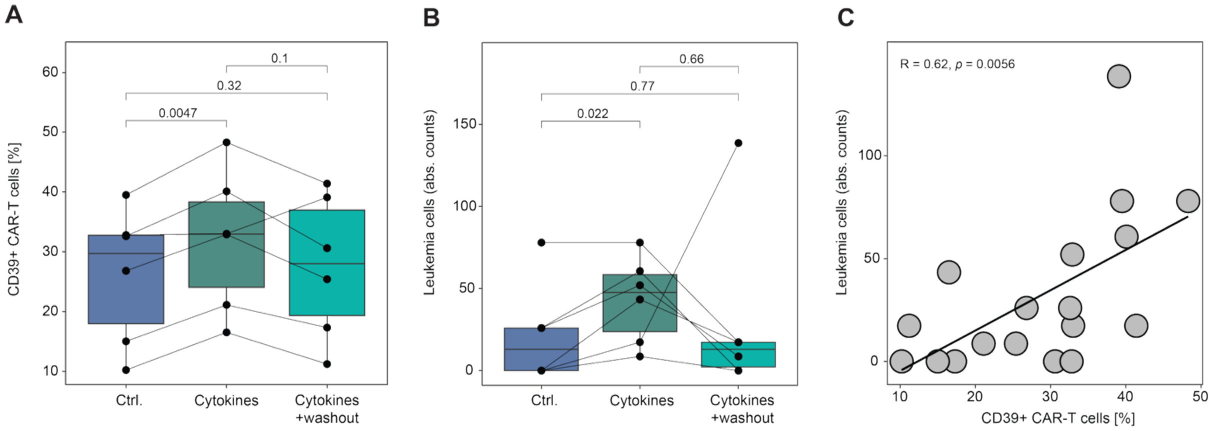

Figure R5. Cytokine washout experiments. A. Percentage of CD39+ CAR-T cells in PBMCs following cytokine pre-treatment prior to CAR-T cell manufacturing. n = 6 per condition. **B.** Absolute number of residual Nalm6 leukemia cells after co-culture with CAR-T cells at a 1:1 effector-to-target ratio. n = 6 per condition. **C.** Spearman correlation between the percentage of CD39+ CAR-T cells after cytokine pre-treatment and the absolute number of residual leukemia cells in the co-culture assay. n = 18.

Ambiguity in Exhaustion and Effector Definitions

• The characterization of exhaustion based on PD1 and CD39 expression may conflate activation with dysfunction. Additional markers (e.g., CD38, Ki67, CD25, CD69) should be shown to clarify the state of these cells. The categorization of "effector-like" subsets also needs refinement, especially within CD4^{+} T-cell clusters where multiple EM-like subsets exist. Heatmaps or supplementary figures delineating full marker expression per cluster would aid interpretation.

Thank you for this comment. We fully agree that in the context of CAR-T cell products, where essentially all T cells have undergone strong activation during manufacturing, it can be challenging to cleanly distinguish activation from dysfunction or precisely define the CAR-T cell state. For this reason, throughout the manuscript, we have deliberately referred to "effector-like", "memory-like" and related "-like" states, to indicate that these phenotypes resemble canonical T cell states, rather than implying fully defined functional states. As suggested by the reviewer, we have expanded our characterization by highlighting additional markers from our cytometry panel in heatmaps and feature plots, thereby displaying the full T cell marker expression profile

of each cluster in both the CD4 and CD8 CAR-T cell compartments (Figure R6A-D). These visualizations are intended to make the basis for our cluster annotations more transparent and to better differentiate predominantly activated from more exhausted phenotypes.

During this process, we noted that some clusters appear less clearly separable depending on the visualization modality (heatmap vs feature plot). To avoid over-interpretation, we have therefore revised a few cluster names to be more descriptive and less definite. We remain open to further suggestions from the reviewer regarding cluster labeling and cell state annotation. The heatmaps and feature plots have now been added to Supplementary Figure 2 of the manuscript.

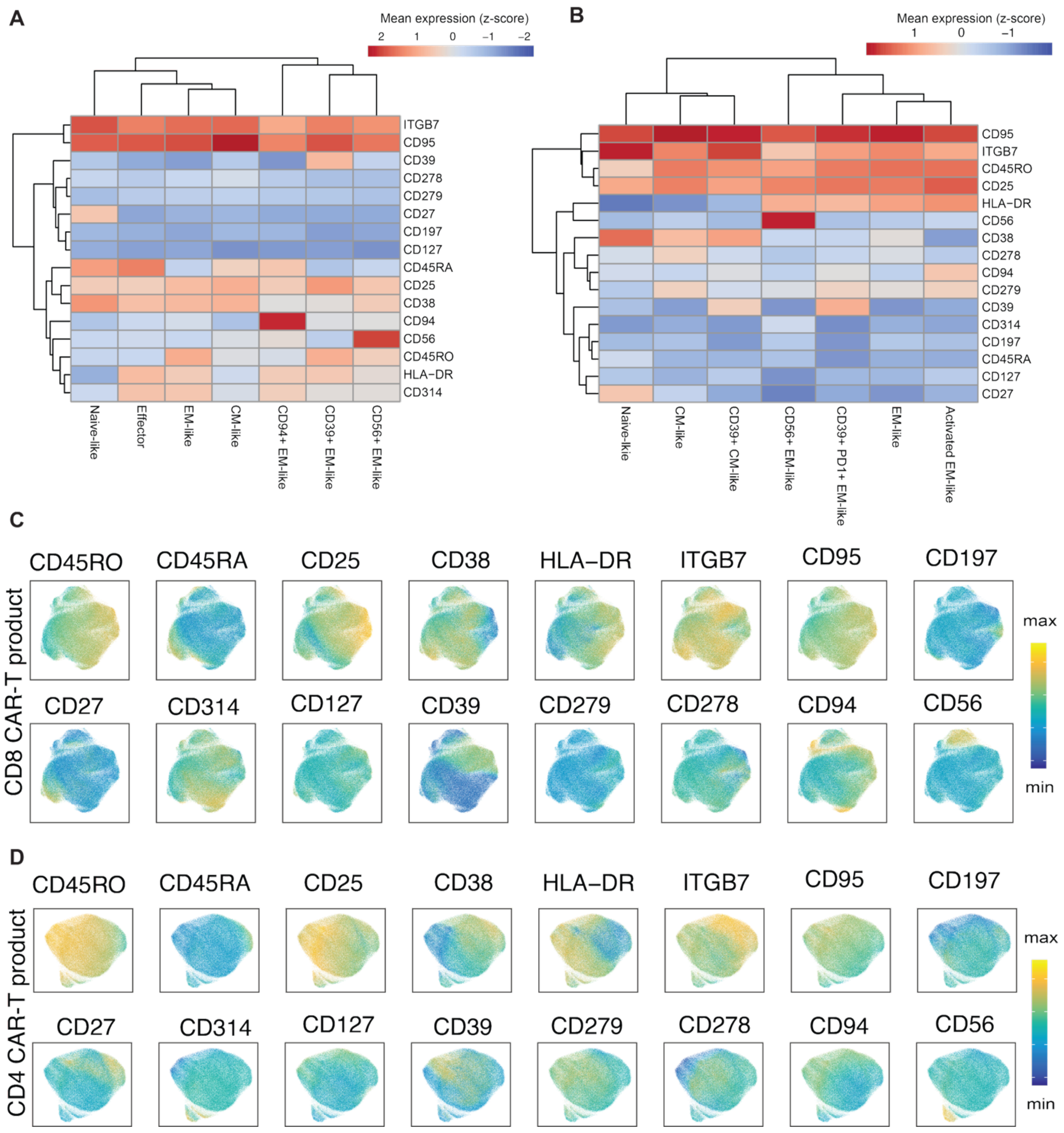

Figure R6. Heatmaps and feature plots of marker intensities in CD8 and CD4 CAR-T cells. A. Heatmap showing z-scored mean expression of T cell markers used for annotation of CD8 CAR-T cell clusters. **B.** Heatmap showing z-scored mean expression of T cell markers used for annotation of CD4 CAR-T cell clusters. **C.** Feature plots showing expression intensities of T cell markers in CD8 CAR-T cells. **D.** Feature plots showing expression intensities of T cell markers in CD4 CAR-T cells.

Tumor Burden and Response Dynamics

• Tumor burden is a known predictor of CAR T-cell outcomes, yet corresponding data for responders vs. non-responders in the low-dose group are not presented. In addition, the duration of response and persistence in the absence of naïve or central memory cells should be addressed. The authors should comment on follow-up time and long-term outcomes.

We thank the reviewer for highlighting the importance of tumor burden. We would like to use this opportunity to bring this into the context of our cohort and expand on response dynamics, and long-term outcomes. First, to address long-term outcomes in the low-dose cohort, we generated Kaplan-Meier curves for overall survival (OS) and progression-free survival (PFS) restricted to low-dose patients. Although no comparison reached statistical significance due to the limited sample size, low-dose responders showed a clear trend toward improved OS and PFS compared with low-dose non-responders (Figure R7A), indicating that responses in this group are not merely transient.

Next, we examined tumor burden in responders versus non-responders. Because our cohort comprises different B-cell malignancies, we did not have a unified volumetric measure of tumor burden and therefore used lactate dehydrogenase (LDH) levels as a proxy. Within the low-dose cohort, non-responders displayed a non-significant trend towards higher LDH levels compared to responders, whereas in the overall study population, LDH levels did not differ significantly between responders and non-responders (Figure R7B). To further evaluate the impact of tumor burden, we stratified patients into high- and low-LDH groups and plotted Kaplan-Meier curves for OS and PFS (Figure R7C). A significant difference was observed for OS but not for PFS, suggesting that tumor burden contributes to long-term survival but is not the sole determinant of disease control.

We then investigated how our proposed biomarker relates to long-term outcome in the context of tumor burden. Patients were grouped according to high versus low numbers of biomarker-associated CAR-T cells (CD27-CD39-), and we created a composite variable combining LDH status (high/low) with biomarker status (high/low). Kaplan-Meier analyses based on this composite stratification showed that patients with low LDH and high numbers of biomarker-positive CAR-T cells had the best OS, whereas those with high LDH and low biomarker levels had the poorest OS (Figure R7D). For PFS, a similar pattern emerged in which patients with high biomarker levels had the

most favorable PFS irrespective of LDH status, consistent with the lack of a strong LDH effect on PFS when considered alone (Figure R7D).

Taken together, these analyses indicate that tumor burden, as reflected by LDH, does influence long-term outcome, particularly OS, but that the proposed CD27-CD39- biomarker adds important prognostic information beyond tumor burden, especially for PFS. This is in line with our analyses from Figure R2, where univariable logistic regression (Figure R2A) showed only a moderate, non-significant association between LDH (as a proxy for tumor burden) and clinical outcome, but a significant association between our biomarker combination and response, which was not further strengthened by a composite LDH-biomarker variable. Complementary Pearson correlation and Cramér's V analyses (Figure R2B,C) likewise indicated that the biomarker combination does not strongly correlate with LDH or other clinical covariates, supporting the interpretation that LDH and our biomarker capture largely independent aspects of disease biology and CAR-T cell efficacy. Jointly, this supports the view that our biomarker captures qualitative features of CAR-T cell efficacy that are important for disease control rather than simply mirroring baseline disease load. These results have now been implemented in Supplementary Figure 1 and Supplementary Figure 4.

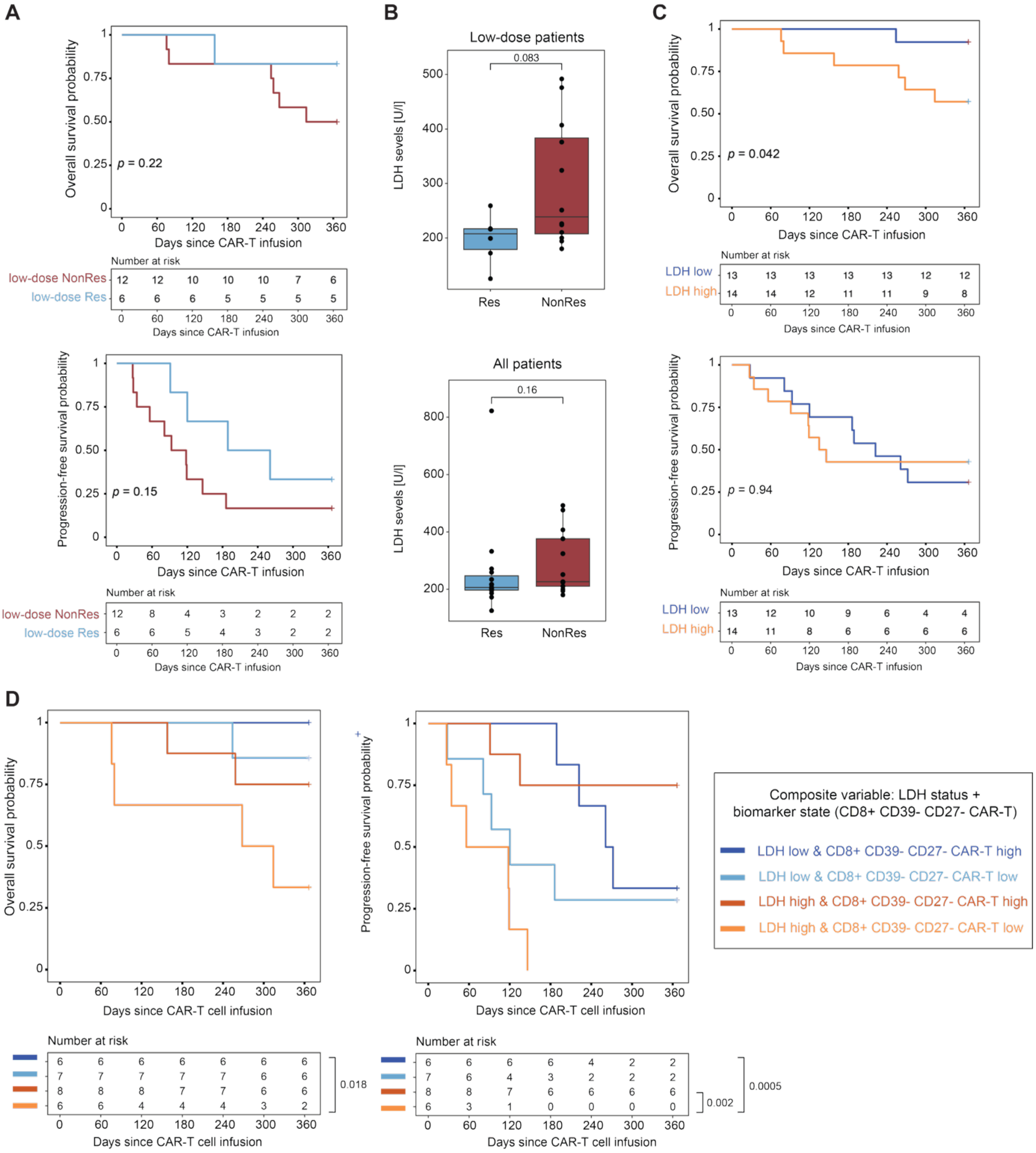

Figure R7. Impact of tumor burden on long term outcomes. A. Kaplan-Meier curves for low-dose patients showing overall survival (OS, top) and progression-free survival (PFS, bottom). P values were computed using the log-rank test. **B.** Lactate dehydrogenase (LDH) levels as a proxy for tumor burden in low-dose patients (top, n = 18) and in all patients from the original cohort (bottom, n = 28). **C.** Kaplan-Meier curves for all patients stratified by LDH level, showing overall survival (OS, top) and progression-free survival (PFS, bottom). P values were computed using the log-rank test. **D.** Kaplan-Meier curves for all patients stratified by a composite variable combining LDH level and the absolute number of CD8+CD27-CD39- CAR-T cells. Pairwise group differences were assessed with log-rank tests. Only significant comparisons are indicated.

Inconsistencies Between Relative and Absolute Metrics

• Despite stating that absolute cell numbers are more predictive than relative frequencies (Discussion lines 444–446), the primary analyses rely heavily on relative abundance. Consistent quantification and reporting of both metrics across figures would be more appropriate.

We fully agree with the reviewer that our initial analyses in Figure 1 of the main manuscript relied predominantly on relative abundances. We originally introduced the concept of absolute CAR-T cell counts in Figure 2 of the main manuscript and have consistently used absolute numbers whenever we analyzed the CAR-T cell product itself. Importantly, Figure 3 (pre-manufacturing PBMCs) and Figure 4 (single-cell sequencing data of PBMCs) of the main manuscript relate to pre-manufacturing blood, not CAR-T cells, where absolute counts were not available, and therefore only relative frequencies were reported. In Figure 5 of the main manuscript, counting beads were not included in the experiments, which prevented us from reporting absolute CAR-T cell counts. In response to the reviewer's comment, we have now incorporated counting beads into the new experiments performed as part of the revision. This allowed us to report absolute numbers in the functional validation experiments (Figure R3) and in additional analyses (Figure R4, Figure R5) or whenever applicable.

We want to emphasize that both absolute numbers and relative frequencies are informative. Absolute counts are particularly useful when comparing different CAR-T cell dose levels that are infused (e.g. out-of-specification, low-dose CAR-T cell infusions), whereas relative frequencies are useful when CAR-T cell doses are comparable (see Figure R4C, D). We have now clarified this more explicitly in the discussion.

• Minor Comments
• Clarify whether equal numbers of cells per sample were used in UMAP projections.

We thank the reviewer for this comment and appreciate the chance to clarify this. We did not use the same number of cells from each sample for clustering and UMAP projections. Instead, we applied a sketch-based subsampling strategy ("atomic sketching") as implemented in the Seurat pipeline (Hao et al., 2023). In brief, for each patient we first selected a representative subset of cells ("atoms") based on leverage scores, which intentionally enriches for rare populations rather than enforcing the same number of cells per sample. These atoms were then used for joint clustering and dimensionality reduction and the remaining cells and their labels were subsequently predicted. As a consequence, the number of cells contributed by each patient to the UMAP is not identical. However, the total cell numbers per patient fall within a comparable range, so no single sample dominates the embedding. We have now clarified this point in the Methods section of our manuscript. Importantly, while this approach is designed to provide an optimal visualization of the data, all statistical analyses were performed using the complete dataset, without any sampling, to ensure an accurate and fair comparison.

• Figure 1E: “Naïve-like CD8 T” is shown on a CD4 plot—please correct or explain if these are double-positive cells.

We thank the reviewer for this careful observation and appreciate the detailed look at our figures. This was indeed a labeling error, and the population in Figure 1E should have been labeled as a CD4 subset. We have corrected the label in the revised figure.

• Define “effector CD4” populations explicitly (e.g., Figure 3FG).

We thank the reviewer for this comment and would like to clarify our definition. In our analyses, the “effector CD4” population in Figure 3F refers to CD4 T cells lacking naïve and central-memory markers CD27 and CD197 (CCR7), lacking the memory marker CD45RO, and expressing CD45RA together with cytotoxicity-associated markers such as CD314 (NKG2D) and CD56 (NCAM1) (Figure R8A). This combination is intended to capture terminally differentiated, cytotoxic effector-like CD4 T cells. However, we remain open to any advice from the reviewer on this matter. This heatmap has now been added to Supplementary Figure 6.

A

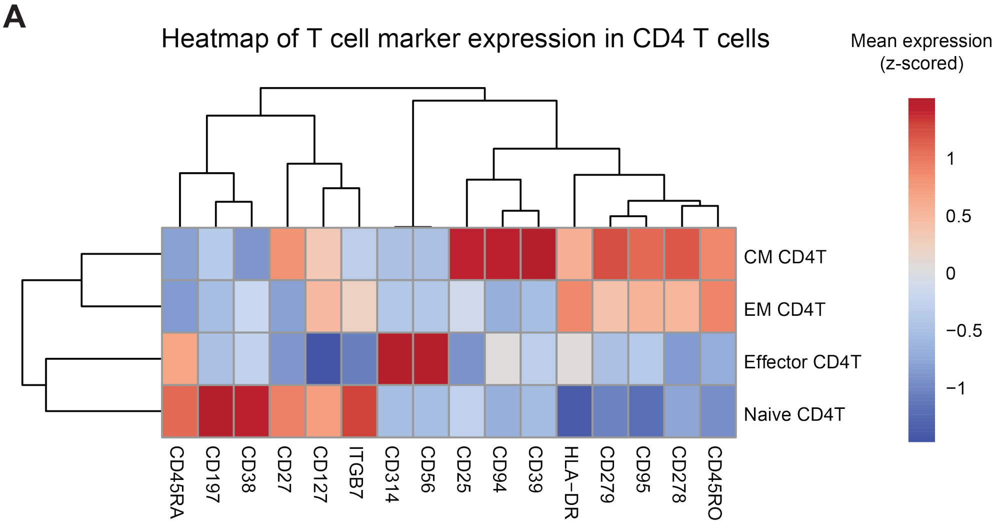

Figure R8. Heatmap of T cell marker expression in CD4 T cells. A. Heatmap displaying mean marker expression (z-scored) of features used for clustering and annotation of CD4 T cells at timepoint of apheresis.

• Specify whether Tregs were excluded from naïve CD4 compartments in Figure 3, as their inclusion may affect observed frequencies.

We thank the reviewer for this comment and the opportunity to clarify how regulatory CD4 T cells (Tregs) were handled. The cytometry panel used in this study included only surface markers and no intracellular staining. Therefore, we cannot definitively identify Tregs, as canonical Treg markers, such as FOXP3, are not present. However, using the surface markers CD25 and CD127, we can define a population of putative Tregs as CD4+CD25+CD127- cells (Figure R9A, B). As shown in the heatmap in

Figure R8A these putative Tregs do not map to the naïve CD4 compartment, which is characterized by lack of CD25 expression. In line with this, the UMAP visualization in Figure R9A indicates that CD25+CD127- CD4 T cells localize predominantly within the central memory compartment rather than the naïve compartment.

Taken together, these data indicate that putative Tregs are not included in the naïve CD4 compartment in Figure 3. At the same time, we acknowledge that, given the absence of FOXP3 and other intracellular markers, Tregs cannot be unambiguously defined in our dataset.

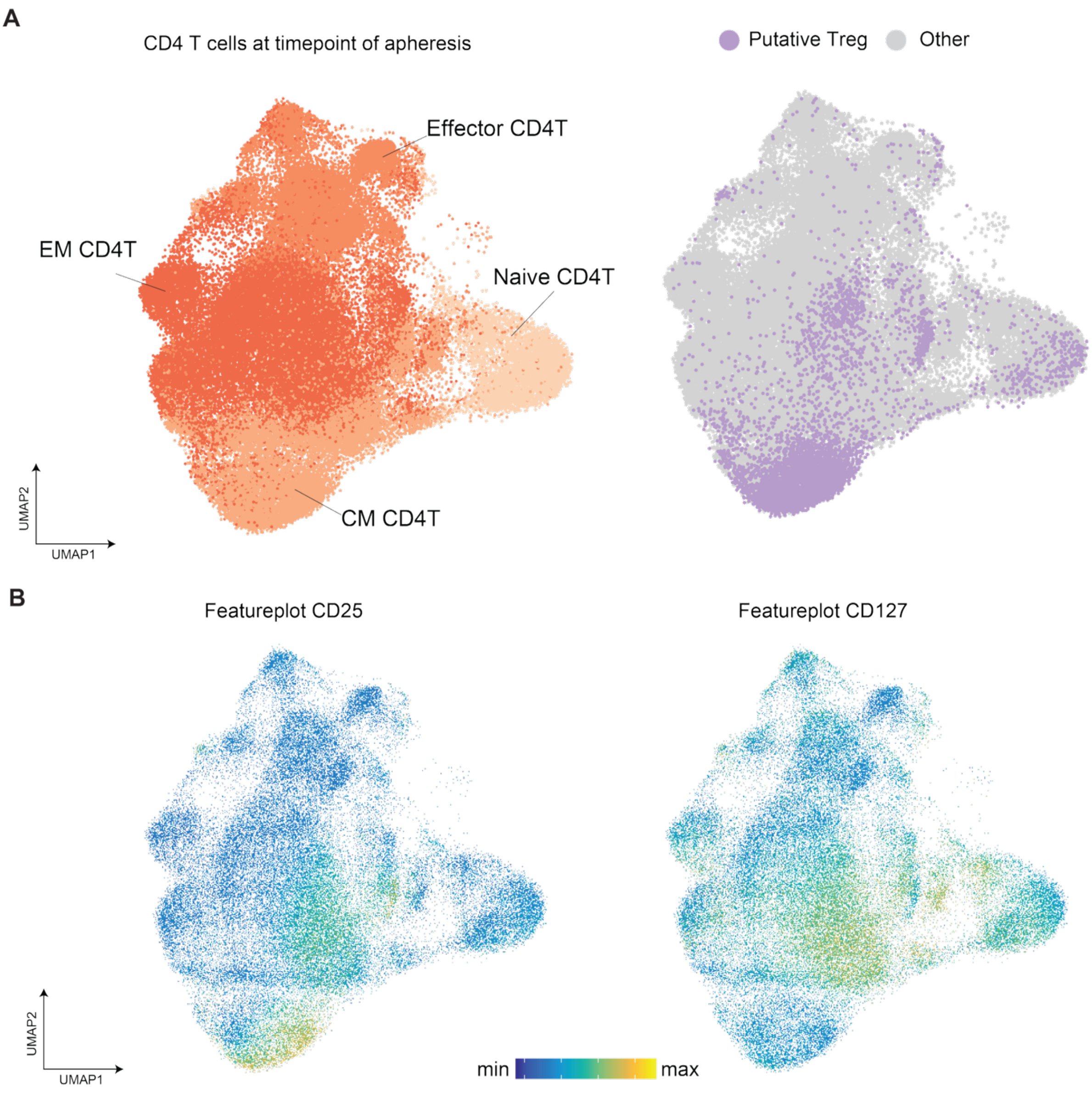

Figure R9. Putative regulatory CD4 T cells at timepoint of leukapheresis. A. Putative regulatory CD4 T cells (Tregs) at timepoint of apheresis identified by CD25 positivity and CD127 negativity. **B.** Feature plots of CD25 and CD127 used to identify Tregs.

• Provide details on starting T-cell numbers and expansion kinetics during manufacturing. Were phenotypic differences observed between products with higher or lower expansion?

We thank the reviewer for raising this important point and would like to provide more information on starting T cell numbers and expansion kinetics. As previously shown in Figure 3C of the main manuscript, low-dose responders are enriched for CD8 T cells in PBMCs at the time of leukapheresis. In response to the reviewer's comment, we have now additionally quantified the total T cell count in the leukapheresis products (Figure R10A). Low-dose non-responders had the lowest absolute T cell numbers in their leukapheresis samples, whereas low-dose responders and high-dose patients showed partially higher starting T cell counts. The frequency of CAR+ cells in the infusion product, as measured by flow cytometry, was comparable within the low-dose patients (Figure R10B). Interestingly, high-dose responders displayed comparatively higher CAR+ cell frequencies in their infusion products. Next, we investigated *in vivo* CAR-T cell expansion. To avoid bias from later events such as death or, in rare cases, a second CAR-T cell infusion, we defined peak expansion as the maximum CAR-T cell level within the first 28 days post-infusion. Within this 28-day window, we did not observe a difference in peak expansion between low-dose responders and low-dose non-responders (Figure R10C,D), suggesting that early *in vivo* expansion alone does not explain clinical outcome in the low-dose setting. By contrast, high-dose responders showed markedly higher peak CAR-T cell expansion (Figure R10C,D). This is consistent with their higher infused CAR-T cell doses, as we observed a correlation between the number of infused CAR-T cells and peak expansion (Figure R10E).

To assess the relationship with long-term outcome, we correlated both the number of infused CAR-T cells and the CAR-T cell peak expansion values with progression-free survival (PFS) using Spearman correlation (Figure R10F). Both parameters showed significant associations with PFS, indicating that the absolute dose of infused CAR-T cells and *in vivo* expansion contribute to long-term clinical benefit. Notably, these relationships did not differ substantially between low-dose responders and non-responders, which points to additional biological or phenotypic factors beyond dose and expansion that are likely influencing response in the low-dose cohort. To address the question of phenotypic differences between products with higher or lower expansion, we stratified patients according to their fold-change expansion and then performed principal component analysis using the cell-type frequencies of CD8 and CD4 CAR-T cell subsets in the infusion product (Figure R10G, H). We did not observe a clear separation between products from patients with high versus low expansion in this space, suggesting that broad compositional features of the CAR-T cell product are not strongly predictive of *in vivo* expansion capacity.

Taken together, these analyses indicate that low-dose non-responders start with fewer T cells at apheresis and that both infused CAR-T cell dose and *in vivo* expansion are associated with long-term outcome. However, within the low-dose cohort, differences

in expansion are modest, supporting the notion that more specific qualitative features of CAR-T cells such as those captured by our CD27-CD39- biomarker may be key determinants of clinical response. These results have now been implemented in Supplementary Figure 1.

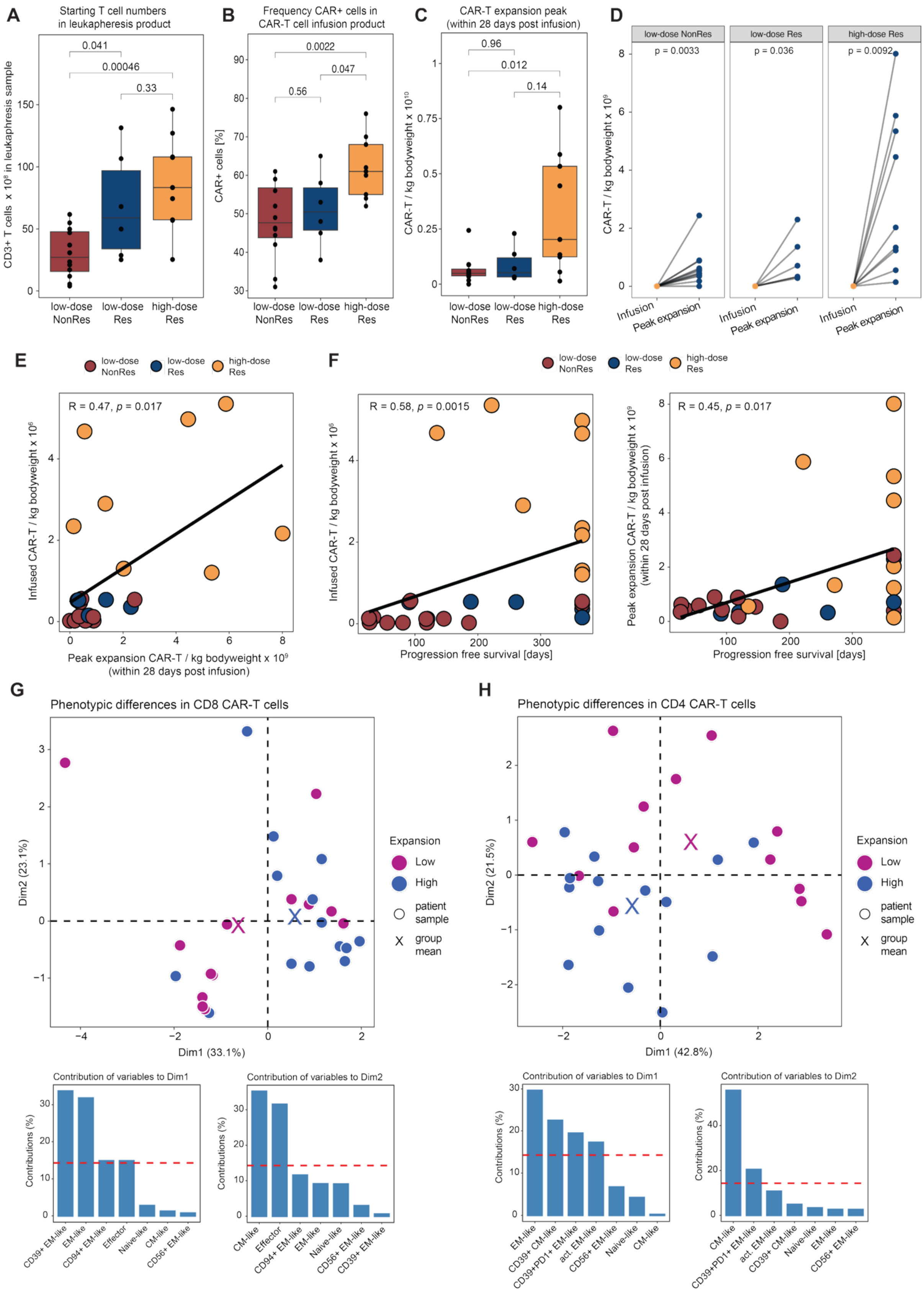

Figure R10. Limited expansion differences in low-dose patients highlight the role of qualitative CAR-T cell features. A. Absolute number of CD3+ T cells in leukapheresis samples. P values were determined with a two-sided Wilcoxon rank-sum test. n = 28. **B.** Frequency of CAR+ cells in the infusion product measured by flow cytometry. P values were

determined with a two-sided Welch's t-test. n = 28. **C.** Absolute number of CAR-T cells per kg body weight at peak expansion within 28 days post-infusion, quantified by quantitative real-time PCR (qPCR) for the CAR transgene. P values were determined with a two-sided Wilcoxon rank-sum test. n = 28. **D.** Paired comparison of the absolute number of CAR-T cells per kg body weight at infusion versus peak expansion. P values were determined with a paired, two-sided Wilcoxon rank-sum test. n = 27. **E.** Spearman correlation between the absolute number of infused CAR-T cells per kg body weight and the absolute number of CAR-T cells per kg body weight at peak expansion. **F.** Spearman correlation between progression-free survival (days) and the absolute number of infused CAR-T cells per kg body weight (left) and the absolute number of CAR-T cells per kg body weight at peak expansion (right). **G.** Principal component analysis of the CAR-T cell infusion product based on CD8 CAR-T cell subset frequencies. Samples are colored according to their fold-change expansion. Variable contributions to PC1 and PC2 are shown below. **H.** Principal component analysis of the CAR-T cell infusion product based on CD4+ CAR-T cell subset frequencies. Samples are colored according to their fold-change expansion. Variable contributions to PC1 and PC2 are shown below.

• Include body weight, prior therapies, and disease burden metrics in the Supplementary Table for cohort characterization.

We have now included body weight, prior therapies, and tumor burden metrics in the Supplementary Table 1.

• Clarify the rationale for selecting subsets of total CD8/CD4 cells for analysis (e.g., 66k out of 10M).

We thank the reviewer for this comment and the opportunity to clarify our rationale. For the clustering and UMAP visualizations, we did not use all available cells (on the order of 10 million), because running standard high-dimensional clustering and embedding on the full dataset can be computationally challenging. Instead, we used the atomic sketching strategy implemented in Seurat (Hao et al., 2023). In brief, this approach selects a representative subset ("sketch") of cells from each sample based on leverage scores, which is designed to preserve the overall cellular landscape and to enrich for rare populations. Clustering and UMAPs are then computed on this sketch (for example, ~66,000 cells), and the remaining cells are subsequently mapped back into this reference space or are assigned cell-type labels by prediction. In this way, only a subset of cells is used for computing the embedding, but all cells are ultimately annotated and included in the downstream analyses.

To illustrate that the results are not overly sensitive to the exact number of cells used for the sketch, we repeated the analysis with different subsampling levels, using 121,550 cells (approximately 1.2% of all cells; Figure R11A) and 243,106 cells (approximately 2.4% of all cells; Figure R11B). In both cases, the UMAP landscapes remained highly stable and closely resembled the embedding shown in the main Figure 1C.

Taken together, this sketch-based strategy allows us to perform robust clustering and visualization on very large datasets while preserving the underlying cellular structure, and all downstream quantitative analyses are performed on the complete dataset including all cells.

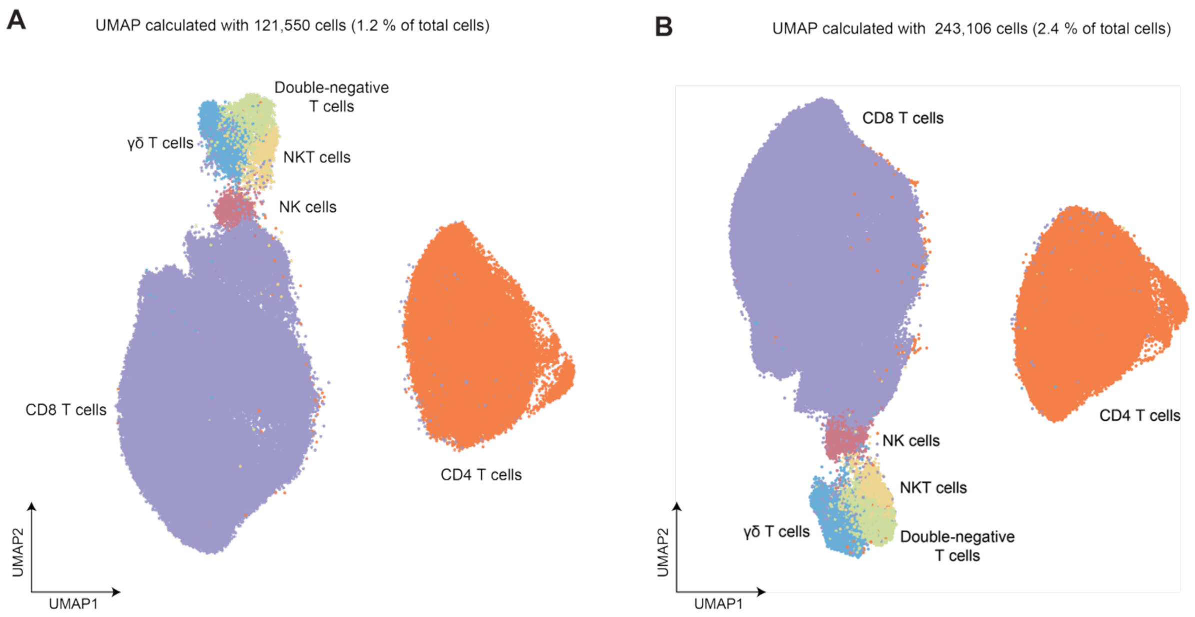

Figure R11. UMAP visualizations calculated with different subsampled cell numbers. A. UMAP of CAR-T cell infusion products calculated with 121,550 sketched cells. **B.** UMAP of CAR-T cell infusion products calculated with 243,106 sketched cells.

• Improve data visualization by color-coding disease subtypes across figures to better interpret disease-specific trends.

We thank the reviewer for this helpful suggestion. In response, we now show UMAP visualizations of CAR-T cells (shown in Figure 1 of the main manuscript) splitted by disease subtype to facilitate interpretation of disease-specific patterns (Figure R12A-C). Together with the analyses shown in Figures R1 and R2, this representation illustrates that cells from different disease subtypes are broadly intermingled without clear disease-driven clustering, indicating that patients from these three entities behave largely similarly at the phenotypic level in our dataset.

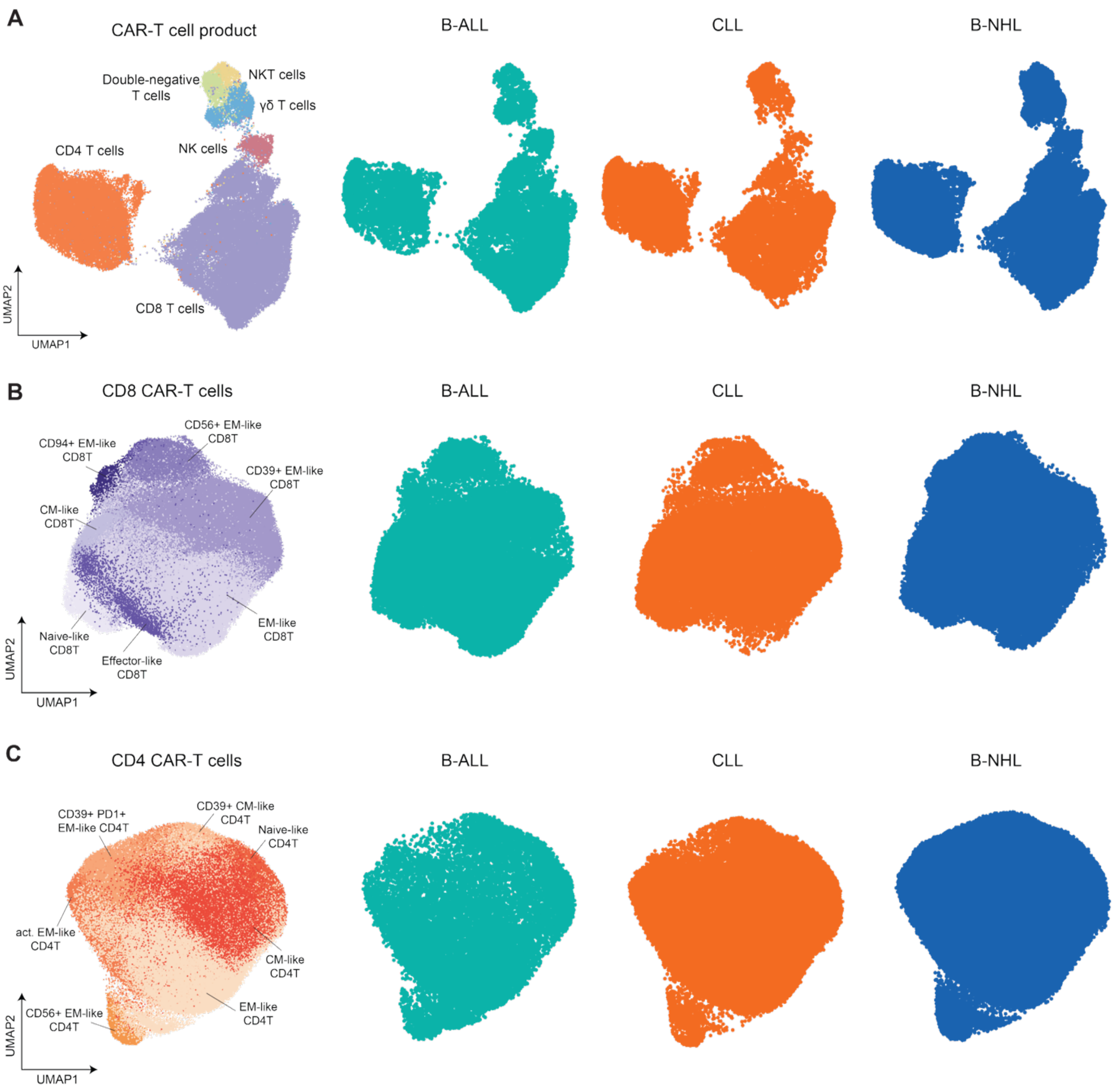

Figure R12. CAR-T cell UMAP visualizations split by disease subtypes A. UMAP visualization of the CAR-T cell product split into disease subtypes. **B.** UMAP visualization of CD8 CAR-T cells split into disease subtypes. **C.** UMAP visualization of CD4 CAR-T cells split into disease subtypes.

• Add a heatmap or supplementary figure showing full marker expression across manually annotated populations (e.g., Figure 3A).

We thank the reviewer for this suggestion. In response, we have now added heatmaps showing the full marker expression profiles for all manually annotated populations corresponding to Figure 3A of the main manuscript (Figure R13A). In addition, we provide separate heatmaps for the CD4 and CD8 T cell UMAPs from Figure 3D and F of the main manuscript, displaying all T cell markers used for clustering (Figure R13B,C). Because only T cell–related markers were used for clustering, UMAP visualization and subsequent annotation of cell clusters, we restrict the heatmaps to

this marker set to accurately reflect the features driving the cluster definitions. The heatmaps have now been added to Supplementary Figure 6.

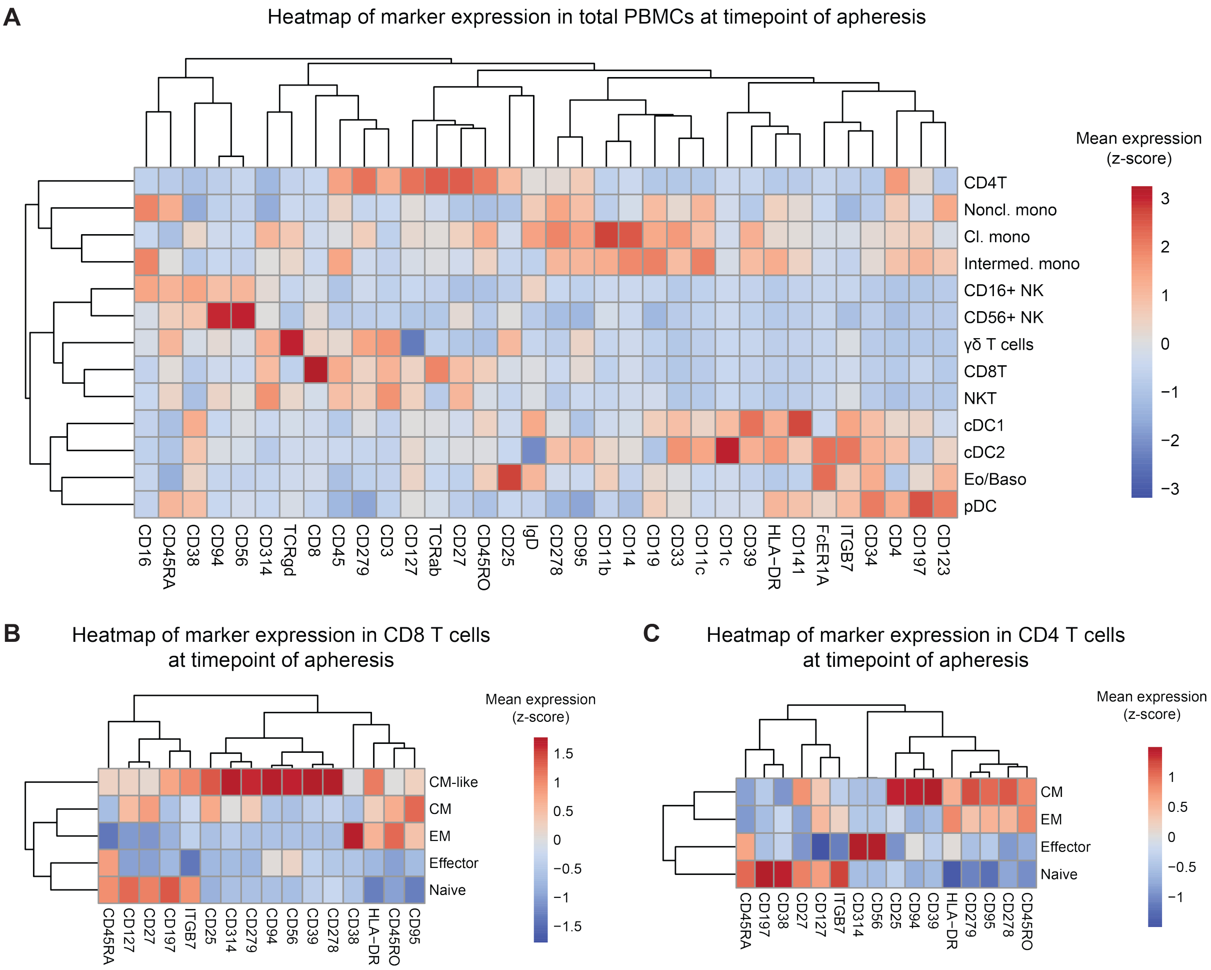

Figure R13. Heatmaps of marker expression in PBMCs at timepoint of leukapheresis A. Heatmap with full marker expression (mean z-scored) in all PBMCs at timepoint of leukapheresis. **B.** Heatmap with mean T cell marker expression (z-scored) in CD8 T cell subsets. **C.** Heatmap with mean T cell marker expression (z-scored) in CD4 T cell subsets.

• Clarify whether CD27⁻CD39⁻ phenotype spans multiple subsets (e.g., CD94⁺EM-like, CD56⁺EM-like) and how these were defined or grouped functionally.

Yes, the CD27-CD39- phenotype does span multiple effector-like and EM-like subsets, which we had previously indicated in Figure 2D and Figure S1C of the main manuscript. We apologize if this was not sufficiently clear and would like to take this opportunity to expand on it. As shown in the newly added Figure R14A,B, we now explicitly highlight the CD8 and CD4 UMAPs of the CAR-T cell infusion product and label the individual subsets that are encompassed by the CD27-CD39- biomarker combination (such as CD94+ EM-like and CD56+ EM-like populations). To further aid interpretation, we also provide the corresponding feature plots for CD27 and CD39 alongside these UMAPs, making it easier to see how the biomarker phenotype overlays with, and spans across, these functionally related effector-like clusters.

It is important to note that these subsets are defined in two different but complementary ways. The “effector-like” and “EM-like” clusters arise from unsupervised clustering in high-dimensional marker space and represent data-driven phenotypic states. In contrast, the CD27-CD39- population is defined by a simple two-marker gating strategy intended to provide a pragmatic and easy-to-implement biomarker that can be used across platforms and laboratories. It is therefore expected and by design, that this two-marker gate captures several closely related effector-like clusters rather than a single high-dimensional subset.

Taken together, these additions clarify that the CD27-CD39- phenotype serves as an operational biomarker that aggregates multiple effector-like and EM-like states identified by unsupervised clustering, thereby linking a simple, clinically applicable gate to the more detailed high-dimensional phenotypic landscape.

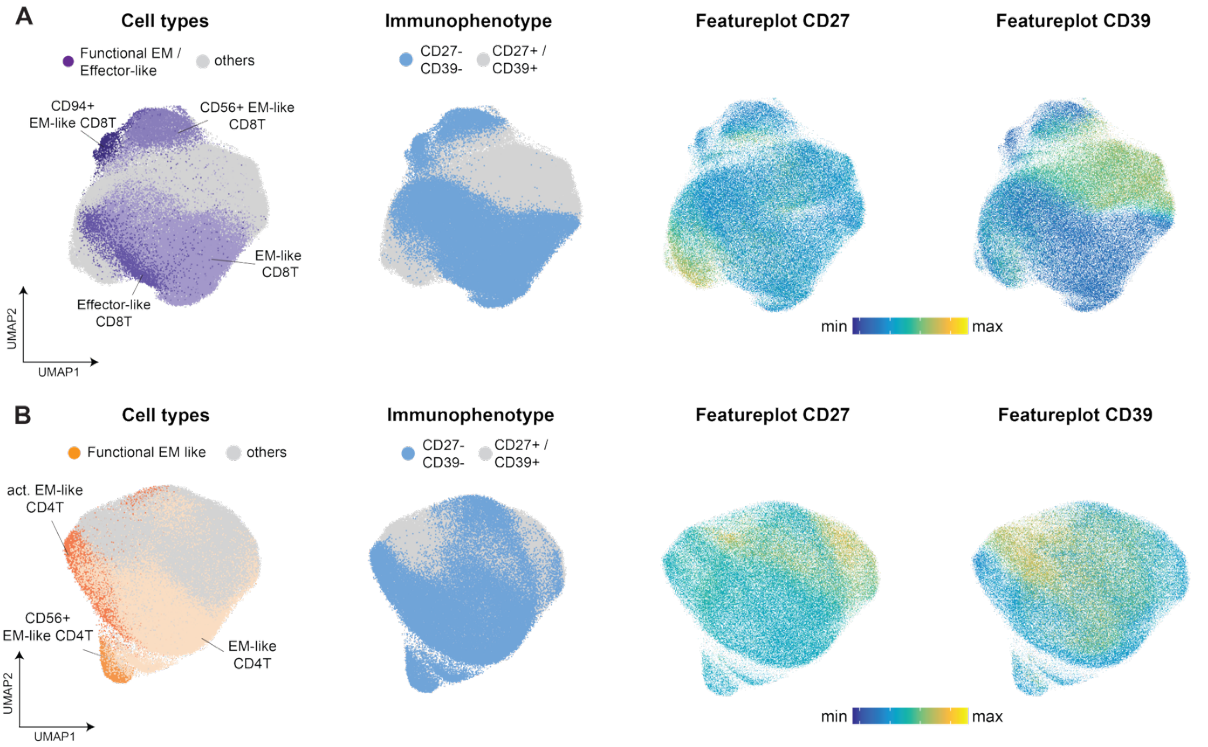

Figure R14. Feature plots of CD27 and CD39 in CAR-T cell infusion products A. UMAP visualizations of the CD8 CAR-T cell product, the cell type labels spanned by our biomarker combination, the resulting immunophenotype, and the corresponding feature plots of CD27 and CD39 **B.** UMAP visualizations of the CD4 CAR-T cell product, the cell type labels spanned by our biomarker combination, the resulting immunophenotype, and the corresponding feature plots of CD27 and CD39.

Overall Recommendation:

This manuscript presents a promising analysis of CAR T-cell phenotypes associated with low-dose therapeutic response. However, to fully support the proposed biomarker strategy and its clinical implications, the authors must address cohort heterogeneity, validate findings functionally, and clarify several methodological and

analytical inconsistencies. With these revisions, the study would represent a meaningful advance in personalized cell therapy optimization.

We thank the reviewer for this constructive assessment. We have addressed the points raised regarding cohort heterogeneity, functional validation of the biomarker combination, the validation in an independent patient cohort and clarification of methodological and analytical aspects. In our view, these revisions have substantially strengthened the manuscript, and we appreciate the reviewer's detailed feedback, which has improved the manuscript's overall clarity and quality.

Reviewer #2 (Remarks to the Author):

In this study, Yousefian et al. present a detailed phenotypic analysis of infusion products from a clinical trial evaluating a third-generation CD19-directed CAR T cell therapy with both CD28 and 4-1BB signaling domains. The focus on low dose responders is clinically relevant, especially in light of manufacturing limitations and the growing need to understand determinants of efficacy. Identifying intrinsic features of the product that correlate with treatment response in this setting is compelling. The integration of full-spectrum flow cytometry, immune profiling, and single-cell proteogenomics provides valuable multidimensional insights. The authors propose that, although the relative frequencies of T cell subsets differ between low and high dose responders, the absolute numbers of functional CAR T cell subsets are comparable and may underlie clinical responses across dosing levels and show that myeloid polarization in pre aphaeresis samples may have a critical impact on CART phenotypes
While the study is ambitious and presents important findings, several critical issues must be addressed to strengthen the robustness, clarity, and impact of the conclusions:

We thank the reviewer for this thoughtful overall assessment of our study and for recognizing the clinical relevance of focusing on low-dose patients. We agree that several aspects of the work require clarification and additional analyses to strengthen the robustness and impact of our conclusions. In response, we have expanded our statistical analyses, added functional validation experiments, validated our biomarker combination in an independent patient cohort and provided additional analyses particularly focusing on in vivo CAR-T cell expansion, tumor burden and long-term outcomes. Below, we address each of the reviewer's specific points in detail.

Major Points:
1. Several groups are testing CAR T cell products in shorter cultures or enhanced with cytokines resulting in a more potent product on a per cell basis. These studies have used much lower cell numbers, for example PMID: 37249512, PMID: 40334157. In light of the demonstration that much lower cell numbers in these

studies and shorter culture times producing a more potent product on a cell for cell basis, comment on the implications for the results presented in this study.

We thank the reviewer for drawing attention to recent work on next-generation CAR-T cell products manufactured in very short culture times and/or with cytokine enhancement. Studies such as those evaluating YTB323 and huCART19-IL18 illustrate that, under optimized conditions, markedly lower CAR-T cell doses can achieve substantial clinical activity and that product quality on a per-cell basis is a key determinant of efficacy. This concept is fully aligned with our central observation that phenotypic features of the CAR-T cell product, in particular the CD27-CD39- subset, are closely linked to clinical efficacy in the low-dose setting.
At the same time, we note that ultra-short manufacturing protocols face practical constraints. In our experience, and as reflected by current commercial practice, manufacturers (e.g. Novartis) have reverted to longer, more conventional culture durations for approved products, at least in part because very short processes may not consistently yield sufficient cell numbers to meet end-product dose specifications. Thus, while rapid manufacturing is attractive from a biological and logistical perspective, it can be challenging to implement broadly in routine practice.

In this context, we believe that our biomarker combination is particularly useful as a complementary tool for future short-manufacturing protocols. By providing a phenotypic readout of therapeutically active CAR-T cells, the CD27-CD39- biomarker may help to identify products that, despite lower total cell numbers, still contain an adequate pool of functionally potent CAR-T cells and might therefore be suitable for clinical use at reduced doses. We have now included this aspect into the discussion of the revised manuscript.

2. Does the low cell number in products reflect inefficiency in cell collection, recent lymphotoxic or lymphostatic chemotherapy, or poor expansion quality T cells, or some identifiable combination?

We thank the reviewer for this important question and want to clarify this point. In our phase I/II dose-escalation study, the low cell numbers in the infused products were protocol-defined (following the safety requirement of the local and federal competent authorities) and not the result of manufacturing failure in individual patients. In contrast, for all patients, yield of sufficient nucleated cells by leukapheresis was mandatory. The trial was specifically designed to explore a range of CAR-T cell doses, including levels that are comparable to those typically seen in out-of-specification (OOS) products where the target cell number specified in the final product release criteria cannot be reached. Thus, the “low-dose” cohort intentionally captures a clinically relevant dose range that, in routine practice, would often be considered too low for release, even though the products meet all other quality criteria.

Insufficient CAR-T cell yield during manufacturing is one important reason why products may become OOS in real-world settings, and this can indeed be influenced by factors such as low T cell numbers at apheresis (e.g. after lymphotoxic or lymphostatic chemotherapy) or suboptimal expansion in culture. However, we want to emphasize that low cell number is not the only cause of OOS in general. Other common reasons include failing predefined quality control parameters, such as inadequate viability, microbial contamination, or vector-related issues (for example, excessive vector copy numbers). By design, such products were not infused in our study, so our low-dose cohort consist of fully release-qualified CAR-T cell products that differ primarily in dose rather than in overall manufacturing quality.

In conclusion, the low cell numbers in our study primarily reflect a deliberate dose-escalation strategy that models the clinically relevant dose range of otherwise suitable but numerically limited products. This design enables us to specifically investigate whether qualitative features of the CAR-T cell product, such as our CD27-CD39-biomarker, can help explain why some patients derive durable benefit even when infused with cell numbers that would typically be considered too low.

3. The manuscript does not report the percentage of CAR+ T cells or fold-expansion data for the infusion products, is there any correlation of CAR frequency and absolute CAR numbers with treatment response? It would be very helpful to include these metrics to enhance the interpretation.

Thank you for raising this important point regarding CAR-T cell frequency, fold expansion, and their relationship to treatment response. In response, we now explicitly report the percentage of CAR+ cells in the infusion product and the in vivo peak expansion of CAR-T cells (Figure R15). In addition, and as context for these dosing metrics, we quantified the total T cell count in the leukapheresis products (Figure R15A), extending our previous observation from Figure 3C of the main manuscript that low-dose responders are enriched for CD8 T cells in PBMCs at the time of apheresis. Low-dose non-responders had the lowest absolute T cell numbers in their leukapheresis samples, whereas low-dose responders and high-dose patients showed partially higher starting T cell counts. The frequency of CAR+ cells in the infusion product, as measured by flow cytometry, was comparable within the low-dose patients (Figure R15B). Interestingly, high-dose responders displayed comparatively higher CAR+ cell frequencies in their infusion products. Next, we investigated *in vivo* CAR-T cell expansion. To avoid bias from later events such as death or, in rare cases, a second CAR-T cell infusion, we defined peak expansion as the maximum CAR-T cell level within the first 28 days post-infusion. Within this 28-day window, we did not observe a difference in peak expansion between low-dose responders and low-dose non-responders (Figure R15C,D), suggesting that early *in vivo* expansion alone does not explain clinical outcome in the low-dose group. By contrast, high-dose responders showed markedly higher peak CAR-T cell expansion (Figure R15C,D). This is

consistent with their higher infused CAR-T cell doses, as we observed a correlation between the number of infused CAR-T cells and peak expansion (Figure R15E).

To assess the relationship with long-term outcome, we correlated both the number of infused CAR-T cells and the CAR-T cell peak expansion values with progression-free survival (PFS) using Spearman correlation (Figure R15F). Both parameters showed significant associations with PFS, indicating that the absolute dose of infused CAR-T cells and *in vivo* expansion contribute to long-term clinical benefit. Notably, these relationships did not differ substantially between low-dose responders and non-responders, which points to additional biological or phenotypic factors beyond dose and expansion that are likely influencing response in the low-dose cohort. To address the question of phenotypic differences between products with higher or lower expansion, we stratified patients according to their fold-change expansion and then performed principal component analysis using the cell-type frequencies of CD8 and CD4 CAR-T cell subsets in the infusion product (Figure R15G,H). We did not observe a clear separation between products from patients with high versus low expansion in this space, suggesting that broad compositional features of the CAR-T cell product are not strongly predictive of *in vivo* expansion capacity.

Taken together, these analyses indicate that low-dose non-responders start with fewer T cells at apheresis and that both infused CAR-T cell dose and *in vivo* expansion are associated with long-term outcome. However, within the low-dose cohort, differences in expansion are modest, supporting the notion that more specific qualitative features of CAR-T cells such as those captured by our CD27-CD39- biomarker may be key determinants of clinical response. These results have now been implemented in Supplementary Figure 1.

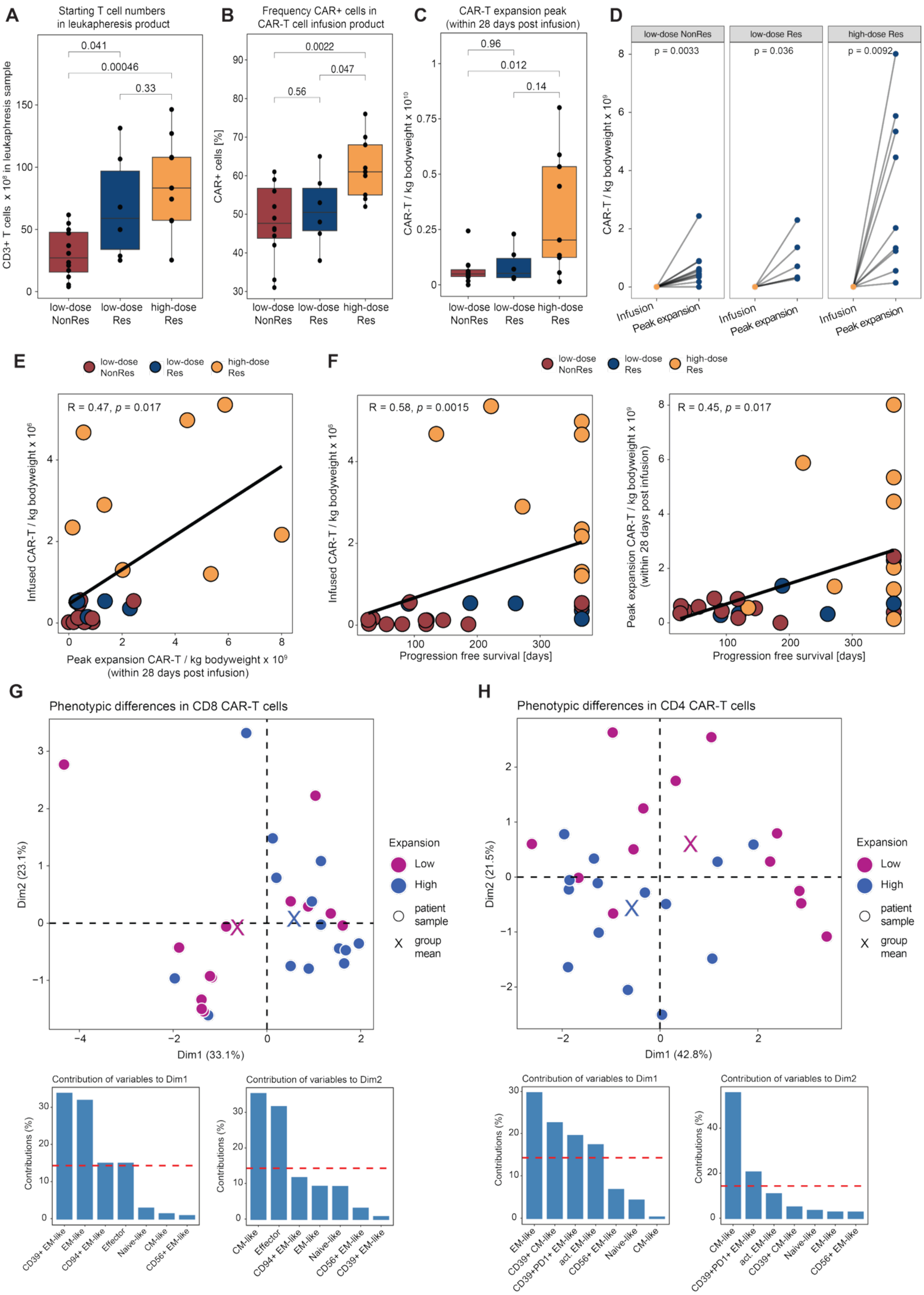

Figure R15. Limited expansion differences in low-dose patients highlight the role of qualitative CAR-T cell features. A. Absolute number of CD3+ T cells in the leukapheresis samples. P values were determined with a two-sided Wilcoxon rank-sum test. n = 28. **B.** Frequency of CAR+ cells in the infusion product measured by flow cytometry. P values were

determined with a two-sided Welch's t-test. n = 28. **C.** Absolute number of CAR-T cells per kg body weight at peak expansion within 28 days post-infusion, quantified by quantitative real-time PCR (qPCR) for the CAR transgene. P values were determined with a two-sided Wilcoxon rank-sum test. n = 28. **D.** Paired comparison of the absolute number of CAR-T cells per kg body weight at infusion versus peak expansion. P values were determined with a paired, two-sided Wilcoxon rank-sum test. n = 27. **E.** Spearman correlation between the absolute number of infused CAR-T cells per kg body weight and the absolute number of CAR-T cells per kg body weight at peak expansion. **F.** Spearman correlation between progression-free survival (days) and the absolute number of infused CAR-T cells per kg body weight (left) and the absolute number of CAR-T cells per kg body weight at peak expansion (right). **G.** Principal component analysis of the CAR-T cell infusion product based on CD8 CAR-T cell subset frequencies. Samples are colored according to their fold-change expansion. Variable contributions to PC1 and PC2 are shown below. **H.** Principal component analysis of the CAR-T cell infusion product based on CD4+ CAR-T cell subset frequencies. Samples are colored according to their fold-change expansion. Variable contributions to PC1 and PC2 are shown below.

4. The responder and non-responder groups at low doses are small (n = 6 for low-dose responders) and include diverse diseases (B-ALL, MCL, FL), while other disease types (DLBCL, CLL) are present in the low-dose non-responder and high-dose responder groups. These biological and clinical differences across groups (such as Tumor Microenvironment context, disease type or prior treatments) represent major confounding factors. Although the sample size is limited, what would restricted analysis in a single disease subtype (ie B-ALL) look like in reproducing or enhancing the reported observation. Conversely, authors should clearly acknowledge and discuss this limitation in the manuscript.

We thank the reviewer for raising this important point. We fully agree that the inclusion of patients with different B-cell malignancies can introduce biological heterogeneity that may influence both CAR T-cell phenotype and clinical outcome. To better understand whether, and to what extent, disease subtype contributes to the observed phenotypic signatures and identified biomarkers, we performed additional analyses (Figure R16). First, we examined whether disease subtypes differ in cellular composition at the time of leukapheresis (Figure R16A). We observed largely similar cellular compositions across subtypes, with no clear clustering by disease in a principal component analysis based on the respective cell type frequencies. We then assessed baseline CD8 and CD4 T cell exhaustion markers per disease subtype (Figure R16B,C), revealing only minor differences with none of them displaying a significant enrichment in any specific disease group at leukapheresis.

To more systematically determine which variables and potential confounders are explained by disease subtype and might therefore affect biomarker signatures, we performed a global η^2 (eta squared) analysis (Figure R16D). Specifically, we tested each numeric variable and potential confounder using a one-way ANOVA with disease subtype as the independent factor and calculated η^2 as an effect size, quantifying the

proportion of variance in each variable attributable to disease subtype. Across all tested potential confounding variables, we observed only moderate η^2 values, indicating that disease subtype explains only a limited fraction of the variability in these features. Because these baseline characteristics did not show strong heterogeneity across disease subtypes, we subsequently evaluated whether T cell/CAR-T cell exhaustion after manufacturing, as reflected by CD39 expression, was influenced by disease subtype (Figure R16E). In both the CD8 and CD4 CAR-T cell compartments, we did not observe a discernible effect of disease subtype on CD39 expression levels in the infusion product. Finally, we assessed biomarker performance stratified by disease subtype (Figure R16G). Importantly, in all disease groups the CD27-CD39- CAR-T cell phenotype was enriched in responders compared to non-responders. However, due to the reduced sample size in each group, statistical significance was only reached in some disease groups.

Together with the positive validation results in an independent cohort (see Figure R17 below), these analyses suggest that while disease-specific biology may contribute to overall outcome differences in a minor fashion, our CD27-CD39- biomarker combination is not primarily driven by disease subtype and appears to capture a cross-disease CAR-T cell feature associated with response at low CAR-T cell doses. These results have now been implemented as Supplementary Figure 7.

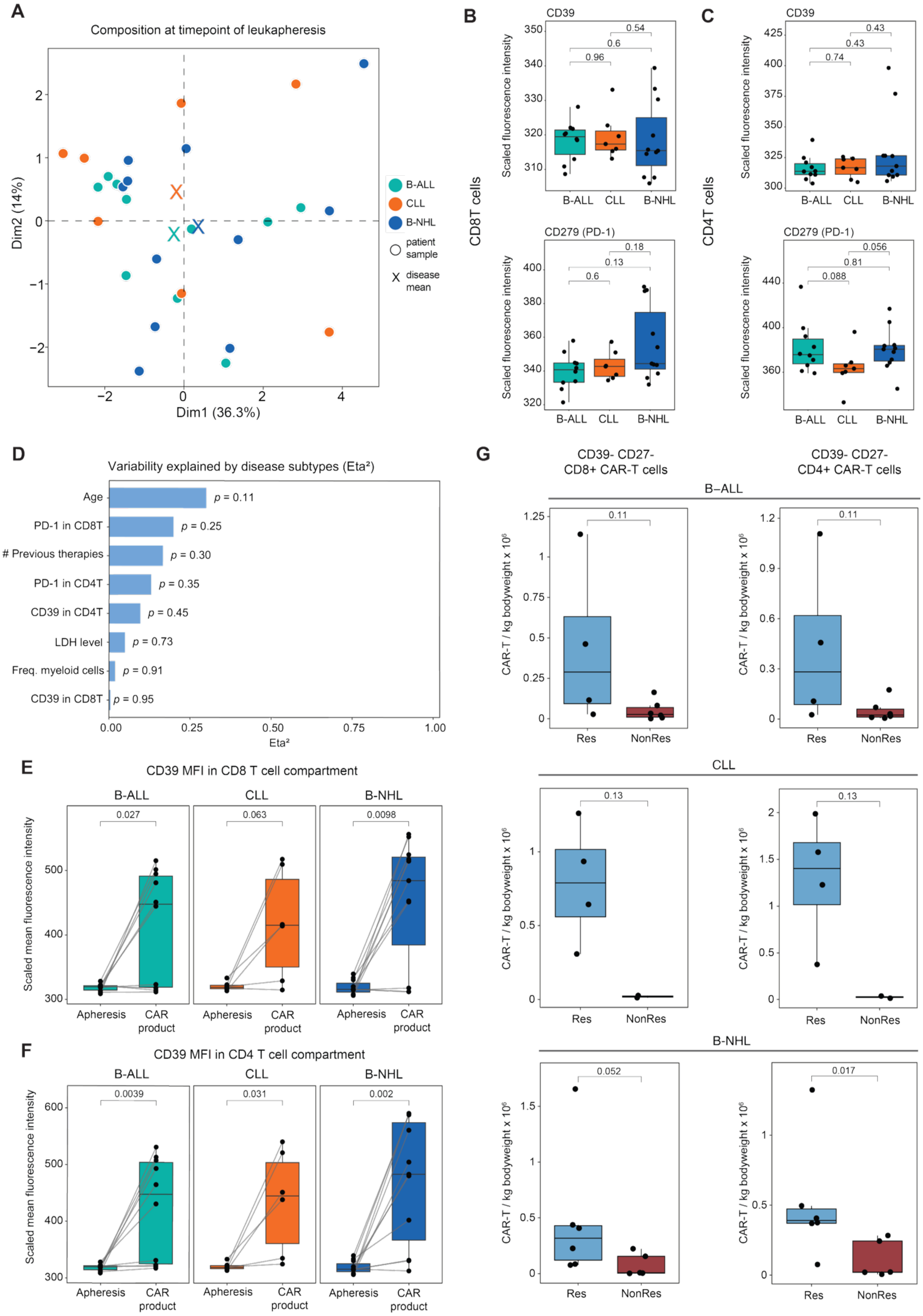

Figure R16. Cohort heterogeneity and individual disease subtype analyses. A. Composition analysis using principal component analysis (PCA) of PBMCs at timepoint of leukapheresis, stratified by disease subtype. **B.** Scaled fluorescence intensity of CD39 and

CD279 (PD-1) in CD8 T cells at apheresis, stratified by disease subtype. P values were determined with a two-sided Wilcoxon rank-sum test. n = 27. **C.** Scaled fluorescence intensity of CD39 and CD279 (PD-1) in CD4 T cells at apheresis, stratified by disease subtype. P values were determined with a two-sided Wilcoxon rank-sum test. n = 27. **D.** Global η^2 (eta-squared) analysis of numeric variables by disease subtype using a one-way ANOVA test. P values are adjusted using Benjamini-Hochberg. **E.** Paired CD39 fluorescence intensity in CD8 T cells at apheresis versus CAR-T cell product across disease subtypes. P values were determined with a paired two-sided Wilcoxon rank-sum test. n = 27. **F.** Paired CD39 fluorescence intensity in CD4 T cells at apheresis versus CAR-T cell product across disease subtypes. P values were determined with a paired two-sided Wilcoxon rank-sum test. n = 27. **G.** Absolute numbers of CD8+CD27-CD39- and CD4+CD27-CD39- biomarker populations stratified by disease subtype. P values were determined with a two-sided Wilcoxon rank-sum test. B-ALL n = 10; CLL n = 6; B-NHL n = 11.

5. The findings are not validated in an independent dataset. Given the small sample sizes and high-dimensional analyses, a validation cohort would greatly improve confidence in the reported findings.

We thank the reviewer for this important comment and fully agree that validation in an independent cohort is essential to increase confidence in the robustness and generalizability of the proposed biomarker. To assess the robustness and generalizability of our proposed biomarker combination beyond our initial cohort and CAR design, we next evaluated its association with clinical response in an independent patient cohort spanning multiple diseases (B-cell non-Hodgkin's lymphoma [B-NHL] and multiple myeloma [MM]), CAR constructs (second-generation CARs with either CD28 or 4-1BB co-stimulatory domains), and targets (anti-CD19, anti-BCMA) (Figure R17). Patients in this validation cohort were treated with four different commercial CAR-T cell products: Breyanzi (anti-CD19, Bristol Myers Squibb), Yescarta (anti-CD19, Kite/Gilead Sciences), Kymriah (anti-CD19, Novartis), and Carvykti (anti-BCMA, Janssen Biotech/Legend Biotech).
Because these were commercial infusion products, we were legally not permitted to perform direct analyses on the CAR-T cell products themselves. We therefore measured CD27 and CD39 expression of CAR-T cells in the first peripheral blood sample obtained after infusion (between day 1 and day 10 for each patient) and used this early time point as a proxy readout of the CAR-T cell product, CAR-T cell activity and biomarker expression *in vivo*. In total, we analyzed samples from 17 MM and 25 B-NHL patients (Figure R17A). In addition, to validate our previously observed myeloid phenotype in pre-manufacturing PBMCs, we assessed CD55 and CD69 expression on PBMCs collected at the time of leukapheresis. As expected for commercially approved anti-CD19 CAR-T cell therapies, the infused CAR-T cell doses were higher than in our phase I/II dose-escalation study. Importantly, however, all MM patients received the same CAR-T cell dose according to the end-product specifications (Figure R17B). In this setting, we found that the biomarker combination measured in CAR-T cells in the first post-infusion PBMC sample could stratify patients into responders and non-responders when using the absolute number of CD27-CD39-

CAR-T cells (Figure R17C). Given the relatively similar infused doses within product groups, the relative frequency of CD27-CD39- CAR-T cells could also discriminate between responders and non-responders (Figure R17D), indicating that within a comparable dose range, both absolute counts and relative frequencies of the biomarker population carry predictive information. When comparing our proposed biomarker to previously reported signatures for anti-CD19 CAR-T cells, our CD27-CD39- combination outperformed these alternatives in patients treated with CD19-directed CAR-T cells in terms of both sensitivity and specificity for clinical response (Figure R17E). Finally, we validated our biomarkers associated with myeloid cells in the premanufacturing blood, including CD55 and CD69 (Figure R17F). Fully recapitulating our initial findings, CD55 expression on non-classical monocytes was higher in non-responding patients, whereas CD69 expression was predominantly enriched on conventional dendritic cells (cDCs) and classical monocytes from responders at the timepoint of leukapheresis (before CAR-T cell manufacturing).

Taken together, these validation data support that the CD27-CD39- biomarker combination is reproducibly associated with clinical response across different diseases, and CAR constructs, suggesting that it captures a core biological property of highly potent and therapeutically effective CAR-T cells rather than a cohort- or product-specific effect. We acknowledge, however, that these validation analyses were performed on CAR-T cells in early post-infusion peripheral blood samples rather than on the infusion products themselves, which represents an important limitation that will need to be addressed in future studies. Moreover, we could validate biomarkers within the myeloid compartment at the time before CAR-T cell generation. These results have now been implemented as Figure 7 of the main manuscript.

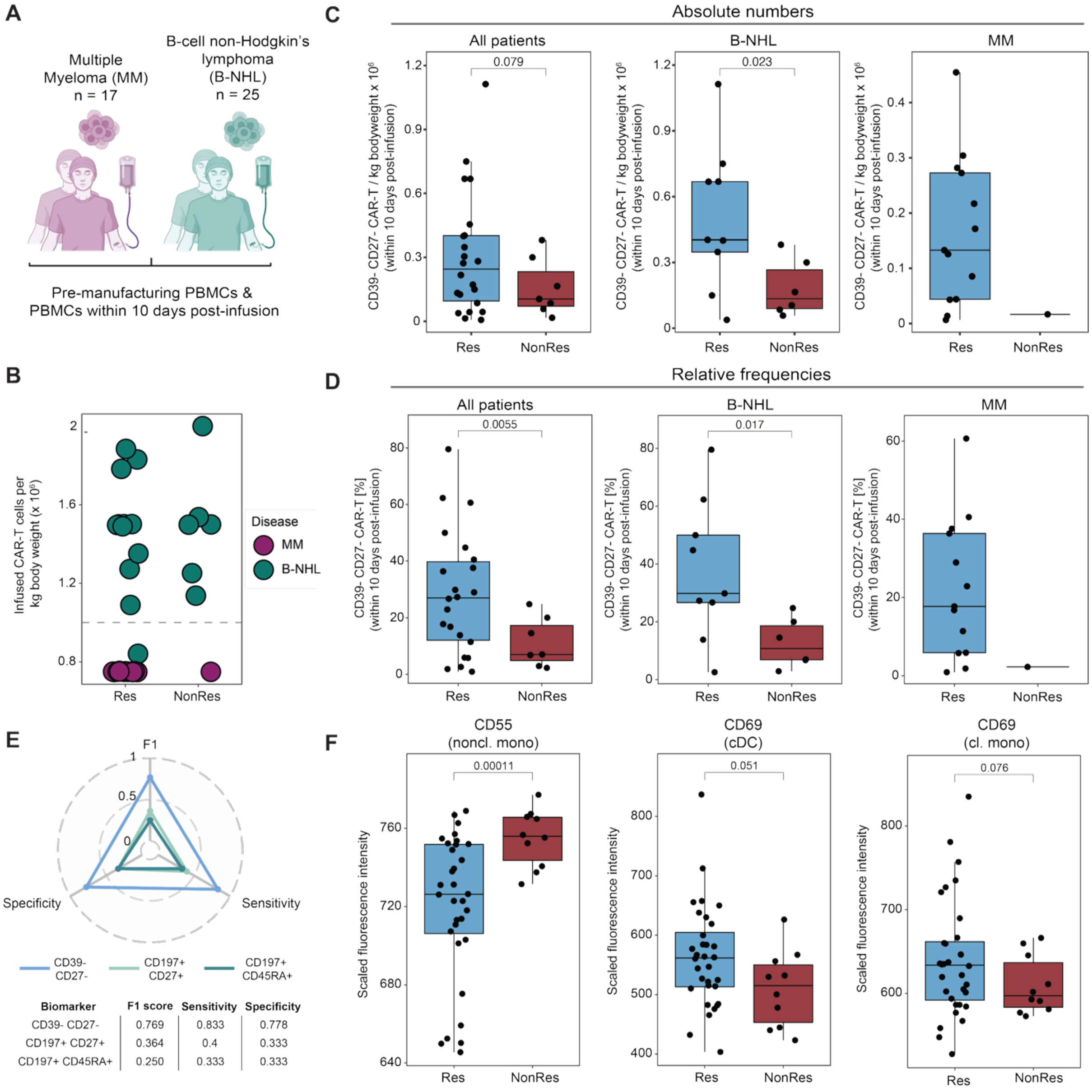

Figure R17. Validation of the CD27-CD39- biomarker across an independent patient cohort. A. Overview of patients in the validation cohort (n = 42). PBMCs at the time of apheresis and CAR-T cells at a single post-infusion time point between day 1 and day 10 were analysed. **B.** Infused number of CAR-T cells per kg body weight. The grey dashed line indicates the threshold used to define low-dose patients in the original cohort. **C.** Absolute number of CD27-CD39- CAR-T cells in the first post-infusion PBMC sample across all patients (left, n = 29), B-NHL (middle, n = 15), and MM (right, n = 14). P values were determined with a two-sided Welch's t-test. **D.** Relative frequency of CD27-CD39- CAR-T cells in the first post-infusion PBMC sample across all patients (left, n = 29), B-NHL (middle, n = 15), and MM (right, n = 14). P values were determined with a two-sided Welch's t-test. **E.** Comparative evaluation of different biomarker signatures using F1-score, specificity, and sensitivity. **F.** Mean fluorescence intensity of CD55 and CD69 in non-classical monocytes (noncl. mono), classical dendritic cells (cDC), and classical monocytes (cl. mono). P values were determined with a two-sided Welch's t-test (n = 42).

6. The authors present an interesting association between M2-like pre-apheresis myeloid polarization and dysfunctional CART phenotypes. To functionally validate the impact of myeloid polarization on CAR T cell quality, the authors should consider complementing cytokine -based assays with co-culture experiments using in vitro generated M1 and M2 macrophages. This would more closely replicate physiologic conditions and enable assessment of downstream CAR-T phenotypes (CD27, CD39 expression, memory/exhaustion states and or cytotoxic potential). In vitro and/or in vivo testing of CAR T cells expanded after these preconditioning conditions would further enhance the translational relevance.

We thank the reviewer for this thoughtful suggestion and fully agree that co-culture experiments with in vitro generated M1/M2 macrophages can more closely approximate physiologic myeloid-T cell interactions than cytokine-based assays alone. In response, we generated M2-polarized macrophages from healthy donor PBMCs and co-cultured these M2 macrophages with autologous PBMCs prior to CAR-T cell manufacturing. Anti-CD19 CAR-T cells were then manufactured from either M2-exposed or untreated PBMCs and subsequently co-cultured with Nalm6 leukemia cells to assess cytotoxic activity. In parallel, we included our previously established cytokine condition (a mix of IL-4, IL-10, IL-13, TGF-β) as an additional control. As shown in Figure R18A, CAR-T cells derived from M2 macrophage pre-treated PBMCs did not show a measurable reduction in killing activity over 44 hours, as assessed by live-cell imaging, compared with CAR-T cells generated from untreated PBMCs. By contrast, reduced killing was observed in the condition where M2-related cytokines were present during the entire culture and CAR-T cell manufacturing time, in line with our original cytokine-based experiments (Figure 6 of the main manuscript, Figure R18B-D, see below).

These data suggest that a sustained presence of regulatory factors may be needed throughout the entire pre-culture and manufacturing process. To investigate this hypothesis, we repeated the cytokine pre-treatment experiments and added a third condition in which the M2-polarizing cytokines were extensively washed out prior to CAR-T cell manufacturing. As shown in Figure R18B-D, cytokine pre-treatment without washout increased CD39 expression and reduced killing activity, whereas cytokine washout restored both CD39 expression and cytotoxic function to levels comparable to the untreated control. These findings suggest that a suppressive myeloid milieu needs to be present throughout the manufacturing process to induce a dysfunctional CAR-T cell phenotype. This is consistent with the observation that in our M2 co-culture experiments, M2 macrophages are not adapted to the manufacturing conditions and tend to die and disappear early, leading to loss of the suppressive milieu early during the manufacturing process.

Taken together, these experiments indicate that an M2-like cytokine environment can transiently impair CAR-T cell function when maintained during manufacturing, and that this effect is reversible upon removal of suppressive signals. Furthermore, our data

supports a model in which a sustained suppressive myeloid milieu, rather than short-term exposure, is required to durably impact CAR-T cell quality. We have now included the washout experiments into the manuscript and adapted the manuscript accordingly.

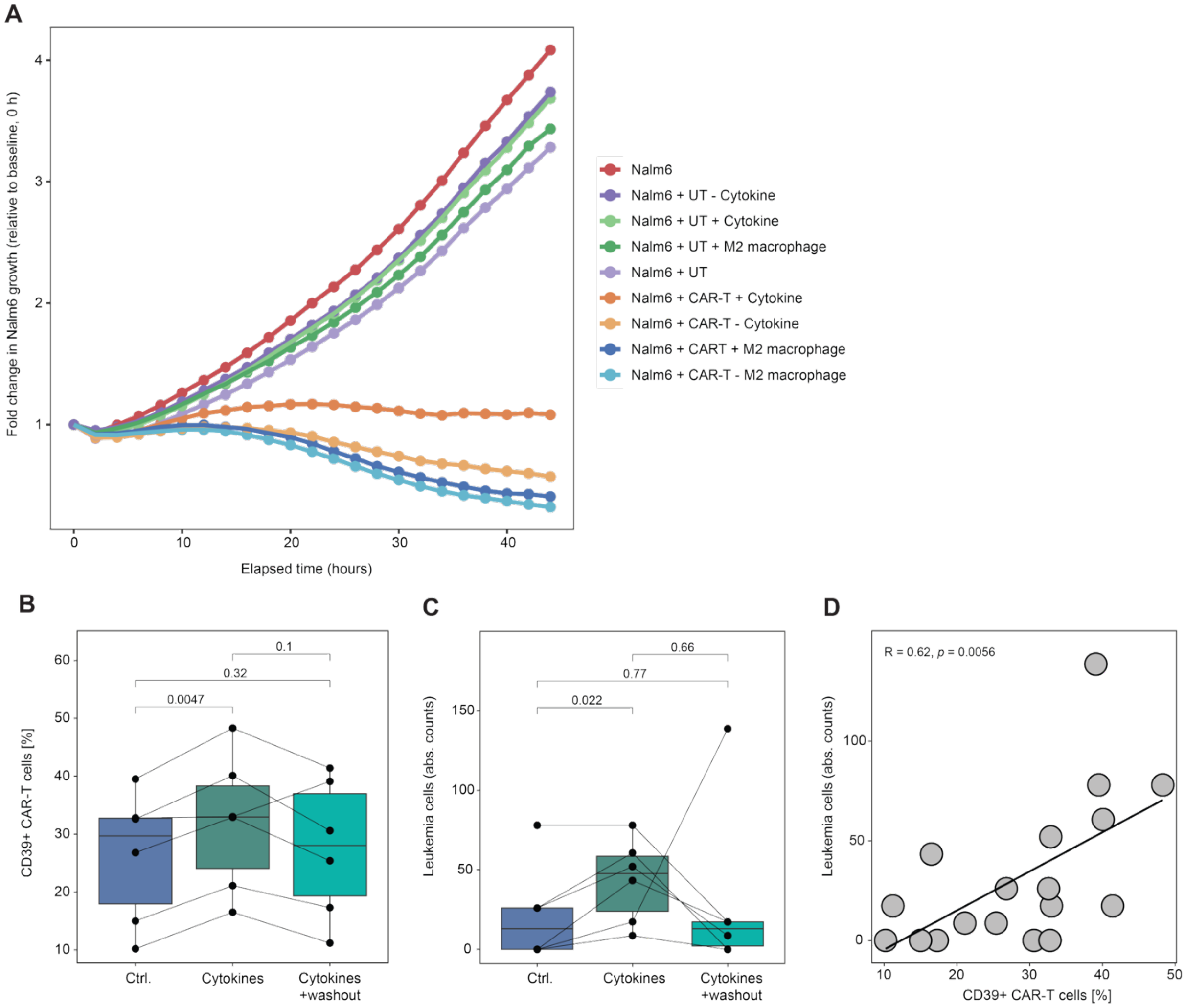

Figure R18. The myeloid milieu can transiently shape CAR-T cell phenotype and functionality. A. Nalm6 leukemia cell growth assessed with live-cell imaging across different conditions. All conditions used an effector-to-target ratio of 1:1. UT = untransduced T cells. **B.** Percentage of CD39+ CAR-T cells in PBMCs following cytokine pre-treatment prior to CAR-T cell manufacturing. n = 6 per condition. **C.** Absolute number of residual Nalm6 leukemia cells after co-culture with CAR-T cells at a 1:1 effector-to-target ratio. n = 6 per condition. **D.** Spearman correlation between the percentage of CD39+ CAR-T cells after cytokine pre-treatment and the absolute number of residual leukemia cells in the co-culture assay. n = 18.

Minor points

The authors state that “manual annotation of clusters was conducted based on marker expression and expert knowledge.” Please specify which markers were used to define key populations.

We thank the reviewer for this suggestion. In response, we have now added heatmaps showing the full marker expression profiles for all manually annotated populations corresponding to Figure 3A of the main manuscript (Figure R19A). In addition, we provide separate heatmaps for the CD4 and CD8 T cell UMAPs from Figure 3D and F of the main manuscript, displaying all T cell markers used for clustering (Figure R19B,C). Because only T cell-related markers were included in the clustering of these UMAPs, we restrict the heatmaps to this marker set to accurately reflect the features driving the cluster definitions. The heatmaps have now been added to Supplementary Figure 6.

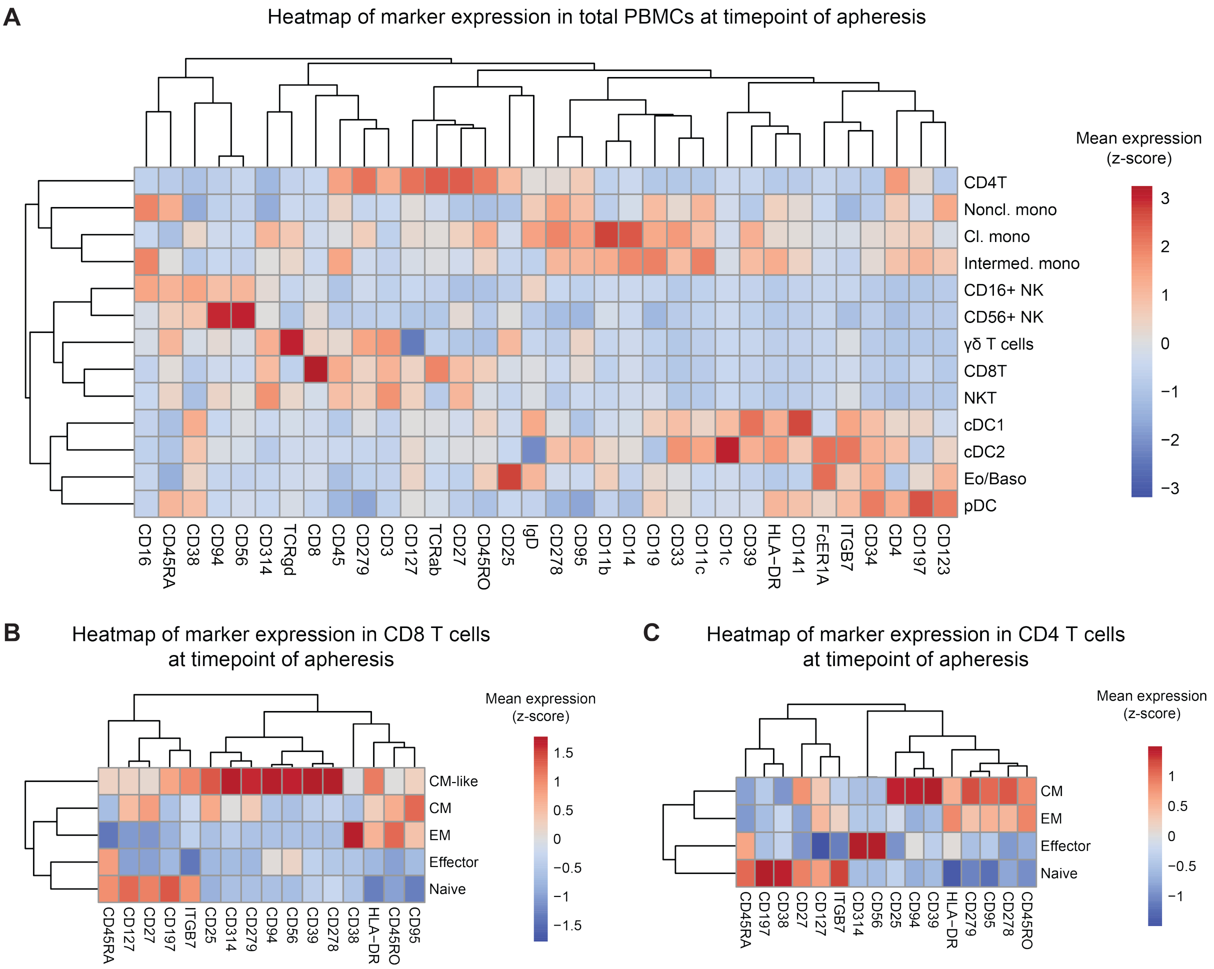

Figure R19. Heatmaps of marker expression in PBMCs at timepoint of leukapheresis A. Heatmap with full marker expression (mean z-scored) in all PBMCs at timepoint of leukapheresis. **B.** Heatmap with mean T cell marker expression (z-scored) in CD8 T cell subsets. **C.** Heatmap with mean T cell marker expression (z-scored) in CD4 T cell subsets.

Line 87: Capitalize "a" in "figure 1a."

Thank you for the comment. We have corrected this now.

The manuscript does not address how the third-generation CAR design (CD28 + 4-1BB signaling) may influence observed T cell phenotypes. This is important, as

costimulatory domains are known to shape expansion kinetics, exhaustion resistance, and memory retention. Please discuss.

We thank the reviewer for raising this important point. We agree that the third-generation CAR design with combined CD28 and 4-1BB co-stimulation can have an influence on CAR-T cell expansion kinetics, differentiation, and exhaustion, and that this context is relevant for interpreting our phenotypic findings.

In our trial, all patients received the same third-generation CD19-directed CAR-T cell product, so we cannot directly disentangle the specific contributions of CD28 versus 4-1BB signaling to the observed CD27-CD39- phenotype within this cohort. However, to assess whether the proposed biomarker is restricted to this particular third-generation design or reflects a more generalizable feature of effective CAR-T cells, we evaluated the CD27-CD39- combination in an independent validation cohort treated with four different commercial second-generation CAR-T cell products: Breyanzi, Yescarta, Kymriah (all anti-CD19, using either CD28 or 4-1BB co-stimulatory domains), and Carvykti (anti-BCMA, 4-1BB) (see response to reviewer point 5 and Figure R17). Because these were commercial products, we could not (for legal reasons) analyze the infusion products directly. Instead, we measured CD27 and CD39 on CAR-T cells in the first peripheral blood sample obtained after infusion (day 1–10) and used this early on-treatment time point as a proxy for product phenotype and early *in vivo* activity. In both disease groups, the absolute number and relative frequency of CD27-CD39- CAR-T cells in this early blood sample discriminated responders from non-responders, and the CD27-CD39- combination outperformed previously reported signatures in terms of sensitivity and specificity (Figure R17C, D, E).

Taken together, these observations suggest that the CD27-CD39- effector phenotype is not uniquely driven by the combined CD28 and 4-1BB architecture of our third-generation construct but rather represents a more general feature of potent CAR-T cells that is detectable across different co-stimulatory domains and targets. At the same time, we acknowledge that our data do not allow a formal comparison of how individual co-stimulatory designs quantitatively shape the frequency or stability of this subset. We have now included this aspect into the discussion section.

Please clarify the statistical approach used for calculating “comparable” absolute numbers of CAR-T subsets between low- and high-dose responders.

We thank the reviewer for this question and are happy to clarify how we derived and compared absolute CAR-T cell numbers. In the first step, we focused on the absolute number of infused CAR-T cells per patient, as this is the relevant dose actually administered. To make these values comparable across patients of different body size, we normalized the dose by body weight and reported the infused dose as the number of CAR-T cells per kilogram of body weight.

To obtain subset-specific absolute counts, we then multiplied this normalized total dose (CAR-T cells/kg) by the relative frequency of each CAR-T cell subset in the infusion product. In this way, for every patient and subset we derived an absolute number of CAR-T cells per kilogram of body weight, which can be directly compared between low- and high-dose responders. For the statistical comparisons, we proceeded in two steps. For each comparison of absolute subset numbers between groups (for example, low-dose responders vs high-dose responders), we first assessed the distribution of the data (normality testing). If the data were approximately normally distributed, we used a two-sided unpaired t-test. If this assumption was not met, we applied a Wilcoxon rank-sum test. When we describe absolute numbers of specific CAR-T cell subsets as “comparable” between low- and high-dose responders, we mean that these tests did not show a statistically significant difference and that the observed effect sizes were small. Given the limited subgroup sizes, we deliberately refrained from fitting complex multivariable models including many covariates, as such models would likely be underpowered and unstable in this setting.

Reviewer #1 (Remarks to the Author)

Overall, the authors have mostly addressed the reviewers' concerns with substantive additions rather than purely rhetorical edits. The revision includes meaningful new analyses to reduce confounding, clearer statistical methodology, added functional validation of the proposed biomarker subset, and an independent external cohort that supports generalizability. A small number of issues remain only partially resolved due to structural constraints (sample size, heterogeneity, and limited access to commercial product material).

We thank the reviewer for their thoughtful overall assessment and for the constructive suggestions throughout the review process. These comments helped us substantially improve both the mechanistic insight and the translational relevance of the study. We also acknowledge that some limitations remain, including sample size, disease heterogeneity and restricted access to commercial infusion products and we have clarified these points in the revised manuscript.

Major concerns and how they were addressed

1) Disease heterogeneity/ confounding by indication

The authors added disease-subtype stratified analyses (composition differences, baseline phenotype comparisons, effect-size screening of covariates, and subtype-stratified biomarker performance). These analyses support that the key signal is not simply an artifact of pooled disease types. This addresses the confounding concern to a large extent, though subgroup Ns necessarily limit statistical decisiveness.

We thank the reviewer for this constructive assessment. We agree that the limited subgroup sizes remain a limitation.

2) Statistical clarity and small-sample handling

They substantially clarified the statistical framework (univariable modeling appropriate for sparse data, explicit handling of separation, and correlation/association matrices to assess confounding). This improves transparency and reduces analytic ambiguity. Power limitations remain inherent but are acknowledged and appropriately handled without overfitting.

We appreciate the reviewer's recognition of the clarified statistical framework. We agree that power limitations remain inherent to the study design.

3) Functional validation of the $CD27^{-}CD39^{-}$ subset

A key improvement is the addition of sorted-subset functional experiments (sequential co-culture challenges, expansion/phenotypic evolution readouts, residual tumor quantification, and cytokine profiling). This directly strengthens the mechanistic plausibility of the proposed biomarker by linking phenotype to functional performance

in vitro. While the model remains simplified, this is a substantive response to the request for functional validation.

We appreciate the reviewer's constructive assessment of the added functional validation experiments.

4) Myeloid/cytokine milieu and reversibility ("washout")
The authors added cytokine exposure plus washout experiments demonstrating that induction of the implicated dysfunction-associated phenotype is reversible/preventable and that functional impairment recovers after washout. This is a strong, targeted response to concerns about causal linkage and kinetic plausibility, with the caveat that it remains a reductionist proxy for in vivo myeloid effects.

We thank the reviewer for this positive and thoughtful feedback on the cytokine exposure and washout experiments.

5) Generalizability across CAR constructs/ products
They added an independent validation cohort spanning multiple commercial products and targets. This materially improves confidence that the signal is not restricted to a single CAR architecture. However, the cohort relies on early post-infusion blood as a proxy phenotype rather than direct infusion-product profiling, which should be viewed as validation of early on-treatment behavior rather than manufacturing phenotype per se.

We thank the reviewer for this important comment and agree with their interpretation. The independent validation cohort strengthens the generalizability of the CD27-CD39- biomarker across different commercial products, CAR targets and disease entities. At the same time, we agree that the lack of direct access to infusion products in this cohort represents an important limitation. Accordingly, we now clarify that these data should be interpreted as validation of early on-treatment CAR-T cell behavior and its association with clinical response, rather than as direct validation of the manufacturing phenotype itself. We have clarified this point in the Discussion section.

Partially addressed/ residual issues
Tumor burden and outcome durability in the low-dose subgroup:
The authors provide survival analyses and use LDH as a burden proxy due to mixed malignancies. This is a reasonable workaround but remains an imperfect substitute for standardized volumetric tumor burden measures and is underpowered in low-dose subgroup analyses.

We thank the reviewer for this comment. We agree that LDH provides only an indirect approximation of tumor burden and cannot replace standardized volumetric tumor burden assessment, particularly in a cohort comprising different B-cell malignancies.

Activation vs dysfunction ambiguity and Treg contamination:
They improved transparency with expanded marker visualization and more cautious cluster naming, but surface-marker panels cannot fully resolve activation-versus-durable dysfunction without orthogonal evidence. Treg concerns are mitigated with proxy gating approaches but remain inferential without intracellular markers.

We thank the reviewer for this helpful comment. We agree that surface marker based phenotyping alone cannot fully distinguish transient activation from durable dysfunction and exhaustion. For this reason, we have revised the annotation of several cell states to make them more descriptive and less definitive.

Reviewer #2 (Remarks to the Author)

The authors have made a substantial and good-faith effort to address initial concerns and have added extensive new analyses and functional experiments that significantly strengthen the manuscript. Overall, the study now represents a high-quality translational contribution with important implications for CAR T-cell biomarker development and manufacturing optimization. However, several central conclusions remain overstated relative to the data and require further refinement. Additional experiments would not be strictly necessary, provided the interpretation and abstract are appropriately revised. However, if the authors wish to further strengthen mechanistic claims, a limited in vivo assessment of product-level potency could be informative.

We thank the reviewer for their thoughtful assessment and constructive guidance. We are pleased that the added analyses and functional experiments are viewed as substantially strengthening the manuscript and its translational relevance for CAR-T cell biomarker development and manufacturing optimization. We also take seriously the concern that some conclusions may have been phrased too strongly relative to the available data. Accordingly, we have revised the relevant sections of the manuscript to avoid overstatement and acknowledge the limitations.

We also appreciate the reviewer’s suggestion that in vivo assessment of product-level potency could further strengthen the mechanistic claims. While we agree that such experiments would be informative, we consider them beyond the scope of the present revision. We have therefore focused on refining the interpretation of our current data.

1. First, the authors have made a commendable effort to validate the proposed CD27-CD39- biomarker in an independent cohort spanning multiple diseases, CAR constructs, targets, and commercially approved products. These analyses support the generalizability of the CD27-CD39- phenotype as a *correlate* of clinical response. However, due to practical limitations, validation was performed using early post-infusion peripheral blood samples rather than manufactured infusion products. As such, these data primarily reflect early in vivo CAR T-cell dynamics rather than intrinsic

properties of the infusion product itself. Consequently, while the biomarker appears predictive of response, the data do not fully support claims that product-intrinsic features determine therapeutic efficacy, particularly in the context of low-dose CAR T-cell therapy. This limitation should be explicitly acknowledged in the manuscript.

We thank the reviewer for raising this important point. We agree that the independent validation cohort supports the broader applicability of the CD27-CD39- biomarker across commercial CAR-T products, targets and disease entities. However, we also recognize that these analyses with early peripheral blood samples do not directly validate the CAR-T cell phenotype in the infusion product. We have therefore revised the manuscript to clarify that these data should be interpreted as supporting the association between the CD27-CD39- phenotype, early on-treatment CAR-T cell behavior and clinical response, rather than as direct validation of the manufacturing phenotype of the infused products. This limitation is now explicitly acknowledged in the Discussion section.

2. The authors performed well-executed functional assays using sorted CAR T-cell subsets, including killing assays, phenotypic stability analyses, and cytokine profiling. These data convincingly demonstrate that CD27-CD39- CAR T cells represent a stable, terminal effector state with high per-cell cytotoxic efficiency. At the same time, the experiments that authors performed also show that CD27+CD39+ CAR T cells exhibit greater proliferative capacity and can give rise to CD27-CD39- progeny, contributing substantially to overall tumor clearance. Thus, while the biomarker identifies a biologically meaningful effector state associated with response, it does not uniquely define a superior or exclusive driver of therapeutic efficacy. Importantly, no experiments directly test the necessity or sufficiency of the CD27-CD39- population at the product level (depletion/add-back or limiting-dose in vivo transfer), nor is an in vivo persistence experiment performed in preclinical models where advantage is proved or demonstrated. Accordingly, the authors should either moderate their conclusions to reflect this limitation or, alternatively, perform in vivo studies to directly assess the relative potency of these subsets. If such in vivo experiments are not undertaken, the authors should explicitly acknowledge that comparative in vivo potency studies would further strengthen the mechanistic interpretation and therefore represent a limitation of the current work.

We thank the reviewer for this very fair point and constructive feedback. We agree that, while our functional assays support the CD27-CD39- phenotype as a stable effector-like state with high per-cell cytotoxic potency, they do not establish this population as the exclusive or superior driver of therapeutic efficacy. We have now acknowledged this in the Discussion and clarified that comparative in vivo potency studies would further strengthen the mechanistic interpretation of this biomarker.

3. The authors added thoughtful mechanistic follow-up experiments using M2 macrophage co-culture and cytokine washout approaches. These data show that

sustained exposure to suppressive cytokines during manufacturing can transiently impair CAR T-cell function and induce CD39 expression, and that these effects are reversible upon removal of suppressive signals. Notably, transient exposure to M2 macrophages alone did not durably impair CAR T-cell quality. These findings are consistent with prior literature on cytokine-mediated T-cell suppression and support a process-dependent and reversible mechanism rather than a fixed, patient-intrinsic determinant. Accordingly, conclusions should be moderated, and the abstract should be revised to accurately reflect this.

We thank the reviewer for the constructive and helpful assessment. We have therefore moderated the corresponding conclusions in the Discussion to more accurately reflect that sustained suppressive cytokine exposure can transiently impair CAR-T cell function.

4. This statement in the response is incorrect: “as reflected by current commercial practice, manufacturers (e.g. Novartis) have reverted to longer, more conventional culture durations for approved products” Kymriah and other CAR T products, as approved, have a culture period that is specified by each regulatory product approved and have not been shortened (and could not without supplemental regulatory filings). Each commercial developer is advancing shorter products in clinical trials with the intent for regulatory submissions. The implication that these shorter culture products will be harder to implement is in this reviewer’s judgement, incorrect.

We thank the reviewer for pointing this out and agree that our previous wording was imprecise. Our statement was primarily intended to refer to very accelerated manufacturing protocols, particularly approaches with culture durations of approximately two days, where achieving sufficient cell numbers to meet current regulatory and end-product specifications may remain challenging. We did not intend to imply that approved commercial products have been shortened and then reverted to longer manufacturing processes. We fully agree that commercial manufacturers are actively working to accelerate CAR-T cell manufacturing and that several shortened manufacturing platforms are being advanced clinically with the aim of future regulatory development.

Reviewer #3 (Remarks to the Author)

I co-reviewed this manuscript with one of the reviewers who provided the listed reports. This is part of the Nature Communications initiative to facilitate training in peer review and to provide appropriate recognition for Early Career Researchers who co-review manuscripts.
